# Nitrile-Functionalized Polysiloxanes with Controlled End Groups for Elastomeric Networks

**DOI:** 10.1021/acspolymersau.5c00080

**Published:** 2025-09-22

**Authors:** Jana Wolf, Patrick M. Danner, Dorina M. Opris

**Affiliations:** † Laboratory for Functional Polymers, 28501 Swiss Federal Laboratories for Materials Science and Technology (Empa), Ueberlandstrasse 129, 8600 Dübendorf, Switzerland; ‡ Department of Materials, ETH Zurich, Vladimir-Prelog-Weg 5, 8093 Zurich, Switzerland

**Keywords:** polar polysiloxane, nitrile polysiloxane, controlled end groups, telechelic polymer, elastomer

## Abstract

High-permittivity polysiloxanes are attractive for applications in dielectric actuators, sensors, energy devices, and electrolytes. A major challenge is the synthesis of polar polysiloxanes with well-defined end groups suitable for controlled cross-linking while minimizing cycle content that can compromise performance. Here, we report the synthesis of polysiloxanes bearing 3-cyanopropyl side groups and aminopropyl or vinyl end groups, with controlled molecular weights and reduced cyclic byproducts. While the reactions in chlorinated solvents predominantly give short chains, the nonchlorinated solvents favor cycle formation. In contrast, hydrolysis–condensation of (3-cyanopropyl)­methyldichlorosilane under solvent-free conditions yields high-molecular-weight polymers (≈14 kg mol^–1^) with only 11% cycles, which are readily removed by toluene extraction. Additional polymer growth is achieved via anionic ring-opening polymerization of isolated cycles, yielding polymers up to 25 kg mol^–1^. Finally, silanol end groups are quantitatively converted into aminopropyl (100%) or vinyl groups (92%), which are useful for cross-linking the polymers to elastic networks with improved structural control.

## Graphical abstract

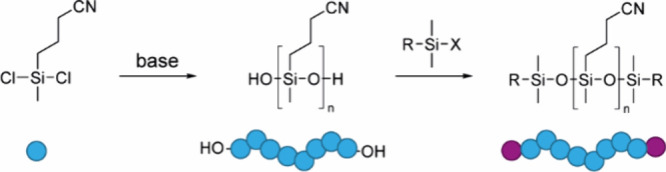

## 1 Introduction

Polysiloxanes modified with polar nitrile groups have been investigated in various applications, including gas chromatography,^1,2^ Li-ion batteries,^3–7^ actuators,^8–10^ and capacitive light-emitting devices,^11^ among others.^12–14^ Despite the potential of nitrile-modified polysiloxanes, there has been little research focused on their synthesis. Several strategies can be employed to achieve these objectives, including the hydrolysis–condensation polymerization of bis­(3-cyanopropyl)­dimethoxysilane or (3-cyanopropyl)­methyldichlorosilane (**1**), ring-opening polymerization under anionic and cationic conditions of 1,3,5,7-tetramethyl-1,3,5,7-tetra­(3-cyanopropyl)­cyclotetrasiloxane (**D**_**4**_ **CN**), and postpolymerization modification of appropriately functionalized polysiloxanes with hydrosilyl or vinyl groups.^15–20^

Modifying a polymer with polar groups increases its glass transition temperature (*T*_g_).^21,22^ However, due to the highly flexible polymer backbone, even polysiloxanes carrying a nitrile group at every repeat unit have a relatively low *T*_g_ of about −50 °C.^23^ Nitrile-modified polysiloxanes are highly viscous liquids and must be cross-linked to achieve suitable elastomers for different applications.^9^

Cross-linked nitrile-modified polysiloxanes can be synthesized by three different approaches: in situ polymerization of **D**_**4**_ **CN** in the presence of a multifunctional cross-linker,^8,24^ and cross-linking via side- ^16,25^ or end groups.^11^ Earlier reports mentioned that with the increase in the content of cyanopropyl groups on polysiloxanes, cross-linking became more difficult to achieve, even when the polymers were modified with vinyl side groups.^25^ Cross-linking via end groups has been less explored, as it requires polymers with defined functional end groups through which cross-linking should occur.^26^

If polysiloxane synthesis is performed without an end-blocker, the product consists of linear chains with silanol end groups and cycles. When bulky and polar substituents, such as cyanopropyl groups, are introduced onto the polysiloxane backbone, increased backbiting is observed during the synthesis.^21,22,27,28^ The resulting polymers are short and contain cycles. Even the synthesis of cycle-free polydimethylsiloxane requires careful selection of the monomer and initiator, as well as optimized reaction conditions.^29^ For efficient cross-linking to elastic materials with low viscoelastic losses, the cycles must be removed, and the cross-linking must be conducted via end groups.^30^

Synthesis of polysiloxanes containing nitrile groups and defined functional end groups is rarely reported. For instance, Suh and co-workers used fuming sulfuric acid to ring-open the **D**_**4**_ **CN** in the presence of an acrylate-functionalized disiloxane as an end-blocker to obtain acrylate-ditelechelic polymers. The molar mass was assessed using ^1^H NMR spectroscopy end group analysis and was found to be below 7000 g mol^–1^.^5^ However, this method does not accurately determine the molar mass of polysiloxanes because cycles and chains cannot be distinguished, and silanol end groups cannot be identified by this method. Additionally, the mechanical properties of the cross-linked materials were not reported. Often, the polysiloxane chains are end-blocked by the trimethylsilyl groups, which cannot be cross-linked.^31^ Jones et al. reported the synthesis of polysiloxanes containing cyanopropyl groups starting from bis­(3-cyanopropyl)­dimethoxysilane and other dimethoxysilane monomers and end-blockers.^25^ While the authors claimed to have vinyl end groups, the mass of the synthesized polymers was rather low.

Despite the early reports on synthesizing poly­(3-cyanopropylmethylsiloxanes) (**P**_**CN**_), achieving high molar masses of polymers with defined end groups free of cycles is still a great challenge. The synthesis of polysiloxanes inevitably results in the formation of low molecular weight cycles due to the inherent equilibrium between cycles and chains.^29^ This equilibrium favors the formation of cycles, particularly when the silicon atom possesses polar side groups.^21,22^ The cyclic compounds lack end groups and thus cannot be chemically cross-linked. If not removed from the polymer before cross-linking, they act as plasticizers, increasing the mechanical losses and aging due to leakage. Therefore, we aimed to develop a reliable synthetic strategy for nitrile-modified polysiloxanes with a defined molar mass and end groups containing a minimal amount of cycles. Additionally, the end groups should be subsequently used for cross-linking to elastic materials with controlled network density.

## 2 Results and Discussion

Scheme 1 gives an overview of the synthetic strategies explored to synthesize poly­(3-cyanopropylmethyl)­siloxane with silanol (**P**_**CN**_ **–OH**), aminopropyl (**P**_**CN**_ **-NH**_**2**_), and vinyl (**P**_**CN**_ **-V**) end groups. These polymers serve as important building blocks for synthesizing nitrile polysiloxane elastomers, which have found numerous applications.

**Scheme 1:**
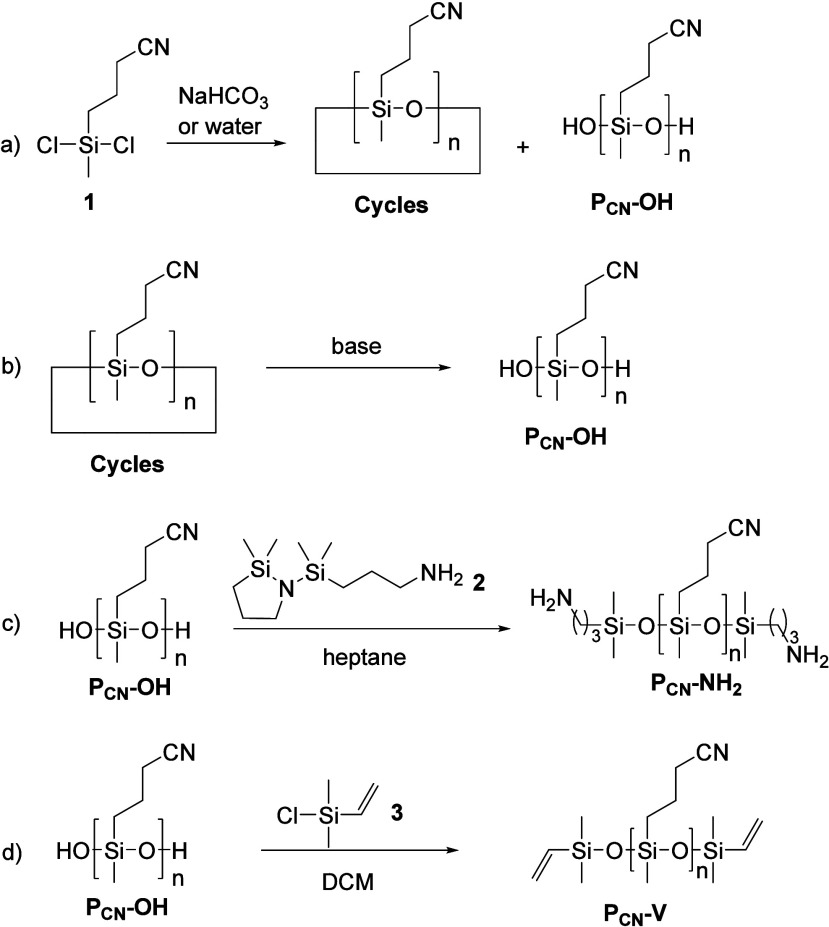
Hydrolysis and Condensation of 3-Cyanopropylmethyldichlorosilane (**1**) Gives a Mixture of Linear Chains Poly­(3-cyanopropylmethylsiloxane) (**P**_**CN**_ **–OH**) and Cycles, Which Can Be Extracted with Toluene (a); the Cycles Are Polymerized by Anionic Ring-Opening Polymerization (AROP) in the Presence of a Base (b); Postpolymerization End Group Functionalization of **P**_**CN**_ **–OH** with 3-[(2,2-Dimethyl-1,2-azasilolidin-1-yl)­(dimethyl)­silyl]­propan-1-amine (**2**) to Give Amino End functionalized Polysiloxane (**P**_**CN**_ ****-**NH**_**2**_) According to Ref 32 (c); and Postpolymerization End Group Functionalization of **P**_**CN**_ **–OH** with Vinyldimethylchlorosilane (3) to Give Vinyl End-functionalized Polysiloxane (**P**_**CN**_ ****-**V**) (d)

### 2.1 Synthesis of Poly­(3-cyanopropylmethylsiloxane) Starting from (3-Cyanopropyl)­methyldichlorosilane (**1**)

We first investigated the hydrolysis–condensation reaction of **1** under different conditions. This step-growth polymerization produces a mixture of cycles and linear chains. Polymer chains of appreciable molecular weight are obtained by optimizing reaction conditions such as the type and amount of solvent, which can substantially impact the ratio of linear and cyclic siloxane products formed. For instance, Brook and co-workers have shown that when the hydrolysis–condensation reaction of dimethyldichlorosilane is conducted in dimethyl sulfoxide, hexamethylcyclotrisiloxane is formed almost quantitatively.^33^

To gain deeper insights into the equilibrium of cycles and chains, we first conducted the hydrolysis–condensation reaction of **1** using acetonitrile, chloroform, dichloromethane, tetrahydrofuran, and toluene as solvents in the presence of sodium bicarbonate without an end-blocker (Scheme 1a). The sodium bicarbonate was suspended in a solvent, and the concentration of **1** was adjusted to 0.8 M for suitable stirring. Generally, dilute reaction conditions favor intramolecular over intermolecular reactions, increasing the amount of cyclic products.

The reaction mixtures were analyzed using gel permeation chromatography (GPC) and ^29^Si NMR (Table 1). GPC analysis alone cannot distinguish whether the low molecular weight product consists of cycles or short oligomers. That is why a second analytical method was needed. Additionally, the siloxane repeat units in both cycles and linear chains have similar chemical environments; therefore, ^1^H and ^13^C NMR spectroscopy do not allow for distinguishing between cycles and linear chains. Therefore, standard ^1^H NMR experiments are not suitable for determining the cycle-to-chain ratio. Additionally, the silanol proton’s acidity prevents its detection in ^1^H NMR recorded in deuterated chloroform due to the fast exchange processes of the hydrogen from the silanol group. This makes the degree of polymerization undeterminable by this method. However, ^29^Si NMR provides distinct signals for siloxane repeat units in cycles and chains, with the signal for siloxane units in linear chains being upfield shifted to −22.5 ppm due to their higher mobility compared to the siloxane units in cycles, which gives a signal at −20.5 ppm (Figure 1). Thus, ^29^Si NMR allows us to determine the cycle-to-chain ratio by integrating the signals of the repeat units. Additionally, the silanol end groups are also detectable in ^29^Si NMR at −14.5 ppm. The molecular weights of the polymers were determined by integrating the signals for the silanol end groups and the chains in the ^29^Si NMR, and the results are presented in Table 1.

**Figure 1:**
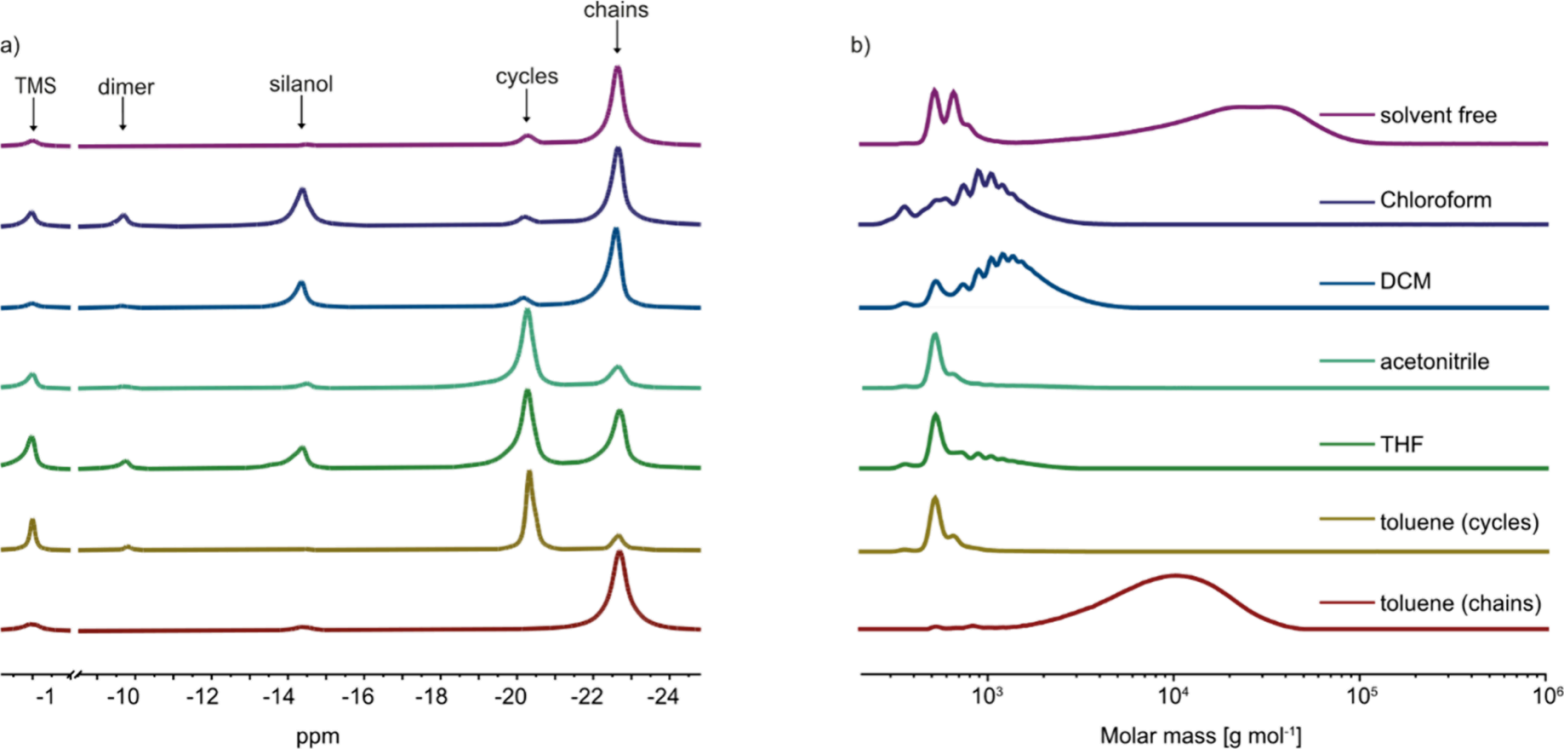
^29^Si NMR spectra using tetramethylsilane (TMS) as a reference (a) and GPC chromatograms in THF and polystyrene standards (b) of the reaction mixture obtained by the hydrolysis–condensation reaction of (3-cyanopropyl)­methyldichlorosilane (**1**) solvent free or in different solvents: chloroform, dichloromethane (DCM), acetonitrile (Me-CN), tetrahydrofuran (THF), and toluene.

**Table 1:** Hydrolysis–Condensation Reaction of 3-Cyanopropylmethyldichlorosilane (**1**) in the Presence of 7 equiv NaHCO_3_ in Different Solvents Obtained with Different Ratios of Cycles and Linear Chains

		^**29**^ **Si NMR**		**GPC**			
	solvent	cycles [%]	*M*_n_ [g mol^–1^]	chains: cycles	*M*_n_ [g mol^–1^]	*M*_w_ [g mol^–1^]	*Đ*
**P**_**CN**_ **–OH** ^**CHCl3**^	chloroform	15	500		780	1000	1.27
**P**_**CN**_ **–OH** ^**DCM**^	DCM	14	760	81:19	1100	1500	1.31
**P**_**CN**_ **–OH** ^**Me‑CN**^	acetonitrile	75	700	30:70	620	770	1.25
**P**_**CN**_ **–OH** ^**THF**^	THF	55	760	54:46	680	840	1.23
**Cycles**	toluene 1st fraction	6	4800	31:69	570	620	1.09
**P**_**CN**_ **–OH** ^**Tol**^	toluene 2nd fraction	80	3900	99:1	5700	11,000	1.90

GPC analysis revealed significant variations in the molecular weight of the reaction mixture obtained using different solvents (Figure 1). For chlorinated solvents, the GPC curves exhibited several low molecular weight product peaks and no high-molecular-weight polymers, while the ^29^Si NMR showed only a small peak for the cycles (14 to 15%) and a big peak for the silanol end groups. Therefore, the low molecular peaks in the GPC were assigned to short oligomer chains with two to 11 repeat units.

The GPC chromatograms of the product obtained with nonchlorinated solvents, such as acetonitrile and tetrahydrofuran (THF), exhibited a dominant peak in the low molecular weight region, which was assigned to **D**_**4**_ **CN**, based on ^29^Si NMR spectra. It should be noted that when the reaction was conducted in acetonitrile, the reaction mixture contained mostly cycles. In contrast, the product of the hydrolysis–condensation reaction in THF produced a mixture of small cycles and short linear chains.

For hydrolysis–condensation in toluene, we observed that 50% of the product remained on the filter paper after filtering the formed salt. Therefore, two different fractions were obtained: one was obtained by evaporating the toluene solvent, and the second was obtained by washing the solid residue with dichloromethane. The first fraction consisted of a rather low molecular weight product, which was assigned to cycles by ^29^Si NMR analysis, while the second fraction consisted of a high molecular weight polymer (Table 1). This indicates that the cycles are more soluble in toluene than the polymer chains. All reactions conducted in a solvent exhibited a signal at −10 ppm in ^29^Si NMR spectra, which was assigned to 1,1,3,3-tetra-3-cyanopropylmethyldisiloxane-1,3-diol. This signal was absent for the reaction conducted without solvent.

Since the hydrolysis–condensation reactions in the presence of a solvent yielded low molar mass cycles and linear chains, we also explored the possibility of conducting the reaction under solvent-free conditions to increase the chain length. Two different reagents were used to convert the dichlorosilane to siloxy, namely sodium bicarbonate and water. It should be noted that **1** was added to the sodium bicarbonate dropwise, and stirring the reaction mixture was difficult with conventional laboratory equipment. On the contrary, adding water dropwise to **1** allowed for easy stirring.

Both ^29^Si NMR and GPC analyses reveal that reaction parameters significantly influence the amount of polymer and cycles formed, as well as the molar mass and polydispersity. ^29^Si NMR spectra of all reaction mixtures show a strong signal for the repeat unit in the polymer chain, while GPC chromatograms indicate that all samples contain high molar mass products (Figure 2). ^29^Si NMR analysis further revealed that 14 to 21% of cycles were present in the product. The cyclic content remained unchanged when the amount of sodium bicarbonate was varied (Table 2). The silanol signal in the ^29^Si NMR spectra was consistently small, indicating the presence of high molecular weight polymers (Figure 2). In contrast, the content of the cycles in the product obtained via hydrolysis–condensation in the presence of water varied depending on the amount of water used. One equivalent of water resulted in 21% cycles and a polymer with a polydispersity index (*Đ*) as high as 13.7. Such a high dispersity is not uncommon for step-growth polymerizations. Additionally, the viscosity increased sharply at the end of the water addition, making stirring difficult. When the reaction was conducted with 2 equiv of water, the amount of cycles decreased, and the polydispersity index was 2.23; however, the molar mass was also reduced.

**Figure 2:**
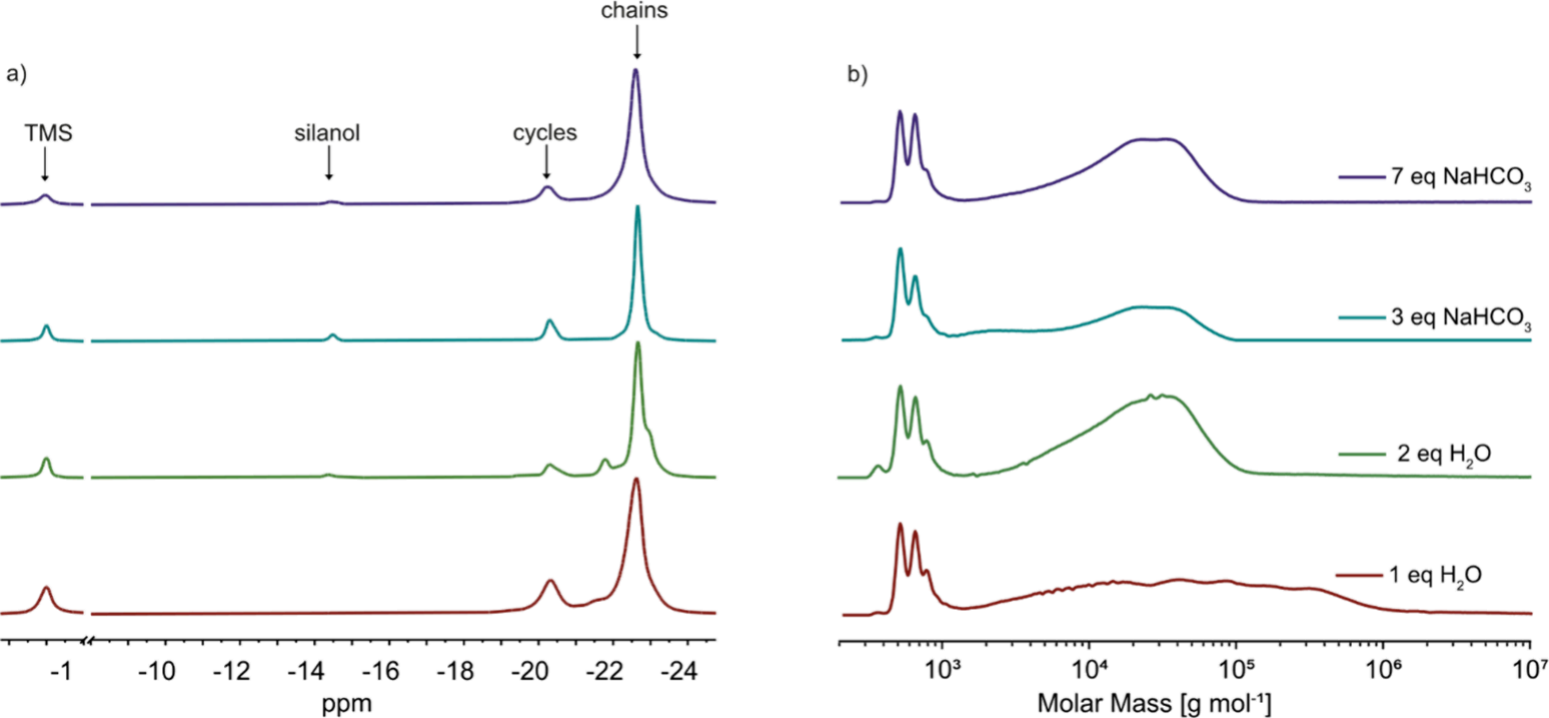
^29^Si NMR spectra (a) and GPC chromatograms (b) of the reaction mixture obtained by the solvent-free hydrolysis–condensation reaction of 3-cyanopropylmethyldichlorosilane (**1**) using different amounts of sodium bicarbonate or water as a base gave a mixture of chains and cycles.

**Table 2:** Characteristics of the Reaction Mixture Obtained by the Solvent-free Synthesis Starting from 3-Cyanopropylmethyldichlorosilane and Using Different Amounts of NaHCO_3_ or Water

			^**29**^ **Si NMR**		**GPC**			
	base	base [equiv]	cycles [%]	*M*_n_ [g mol^–1^]	chains: cycles	*M*_n_ [g mol^–1^]	*M*_w_ [g mol^–1^]	*Đ*
**P**_**CN**_ **–OH**	NaHCO_3_	7	14	11,000	78:22	14,000	27,000	1.85
**P**_**CN**_ **–OH**	NaHCO_3_	3	16	5200	80:20	18,000	33,000	1.87
**P**_**CN**_ **–OH**	H_2_O	2	11	14,000	86:14	15,000	33,000	2.23
**P**_**CN**_ **–OH**	H_2_O	1	21	52,000	83:17	18,000	250,000	13.7

Due to the significant presence of cycles in polymers synthesized via solvent-free hydrolysis–condensation, an effective purification method using toluene extraction was developed and validated by ^29^Si NMR spectroscopy. The polymers initially contained approximately 20% cycles. Because these polar cycles have appreciable molar mass, they cannot be removed by conventional vacuum distillation. However, toluene preferentially dissolves low-molar-mass cycles over linear chains, enabling their extraction from the reaction mixture. The efficiency of this extraction was confirmed by comparing the ^29^Si NMR spectra of the crude polymer, the purified polymer, and the toluene extract (Figure 3). The crude polymer spectrum displays peaks corresponding to both cycles and linear chains. After extraction, the peak at approximately −20 ppm disappears from the purified polymer spectrum and increases in intensity in the toluene phase, indicating successful removal of cycles. The ^29^Si NMR spectrum of the toluene extract shows the presence of both short linear chains and cycles. After three extraction steps, no low-mass cycles were detectable in the polymer’s ^29^Si NMR spectrum, confirming the effectiveness of the purification process.

**Figure 3:**
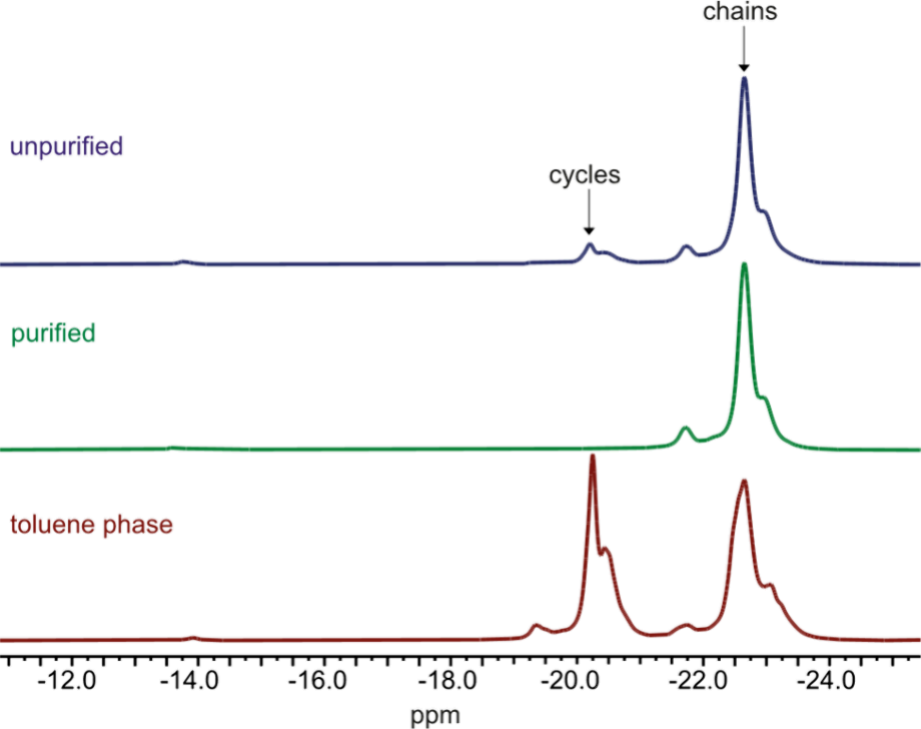
^29^Si NMR spectra of crude polymer (top), purified polymer (middle), and residue of the toluene phase (bottom). By treating the crude polymer with toluene, the cyclic species are removed.

To conclude, the best conditions for synthesizing **P**_**CN**_ **–OH** starting from **1** are by hydrolysis–condensation of cyanopropylmethyldichlorosilane in the presence of 2 equiv of water, followed by the removal of the low-mass cycles by toluene extraction. This reaction was conducted on a large scale, starting with 1 kg of **1** monomer, which will facilitate **P**_**CN**_ **–OH** commercialization and its use in different applications.

### 2.2 Synthesis of Poly­(3-cyanopropylmethylsiloxane) Starting from 3-Cyanopropylmethyltetracyclosiloxane

Next, **D**_**4**_ **CN** obtained from the hydrosilylation of tetramethyltetracyclosiloxane in the presence of allyl cyanide (according to Dietrich et al.^34^) was polymerized using anionic ring opening polymerization (AROP) (Scheme 1b). The polymerization was conducted solvent-free to minimize backbiting.^35^ Considering the initiator, the cation is crucial due to ion pair formation with the active silanolate center, decreasing the rate of polymerization. Bulky cations have delocalized charge, causing the ion pairs to become loose and thereby increasing the silanolate anion reactivity for AROP of PDMS.^36^ Functional groups like nitrile on the silicon atom, which can interact with the cation, increase the polymerization and depolymerization rate.^21,22,37^ First, five different bases were used as initiators, and the polymerization was carried out at 40 °C (Table 3). The products were analyzed using ^29^Si NMR and GPC (Figure 4). The ^29^Si NMR results revealed that all initiators yielded a mixture of cycles and chains, but the ratio of these components differed. Only AROP with tetrabutylphosphonium hydroxide (TBPH) as an initiator gave a product with more linear chains than cycles (89% linear chains). However, the GPC does not show any high-molecular-weight polymers. This may be due to reactive chain ends that are difficult to remove and may undergo depolymerization in THF, the solvent used for GPC analysis. Even quenching the active chain ends does not lead to increased polymer yield. In contrast, it facilitates depolymerization into short chains and cycles (Figures S1 and S2). Because the ^29^Si NMR shows the presence of polymer chains, TBPH was further explored for opening the cycles.

**Figure 4:**
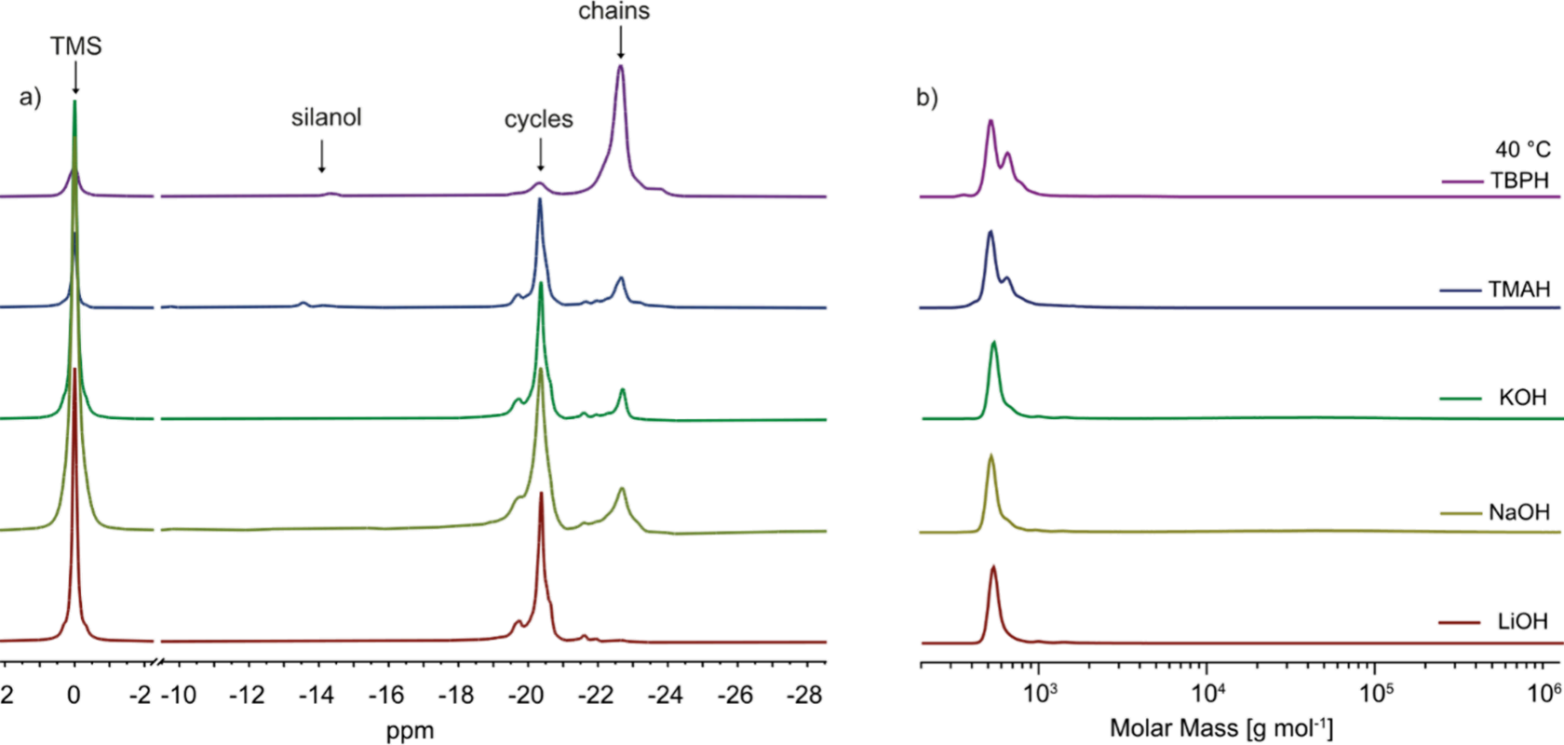
^29^Si NMR spectra (a) and GPC chromatograms (b) of the products obtained by the solvent-free AROP of 3-cyanopropylmethyltetracyclosiloxane (**D**_**4**_ **CN**) with different bases as an initiator gave mixtures with different ratios of chains to cycles.

**Table 3:** AROP of **D**_**4**_ **CN** at 40°C Using Different Initiators and the Characteristics of the Products Obtained as Analyzed by ^29^Si NMR and GPC

			^29^Si NMR		GPC			
	base [mol]	monomer [mol]	cycles [%]	*M*_n_ [g mol^–1^]	chains: cycles	*M*_n_ [g mol^–1^]	*M*_w_ [g mol^–1^]	*Đ*
**P**_**CN**_ **–OH**	TBPH [0.212]	2.12	18	10,000	0:100	640	1000	1.57
**P**_**CN**_ **–OH**	TMAH [0.19]	2.22	67	1400	0:100	600	730	1.23
**P**_**CN**_ **–OH**	KOH [0.20]	2.04	84	21,000	17:83	26,000	57,000	2.21
**P**_**CN**_ **–OH**	NaOH [0.20]	2.12	66	61,000	23:77	31,000	85,000	2.72
**P**_**CN**_ **–OH**	LiOH [0.20]	1.89	97	3400	3:97	550	580	1.05

On the other hand, LiOH and KOH are much less active in opening the cycles in AROP. Using LiOH as an initiator does not open the cycles at all. This is supported by the fact that there is no signal of linear chains in the ^29^Si NMR and no high molecular weight polymer visible in GPC. A similar case is the use of KOH as an initiator. The AROP product with KOH consists of 16% linear chains, as determined by ^29^Si NMR, and 17% high molecular weight polymer, as determined by GPC. These two initiators were found to be poor at opening the cycles.

For the last two bases, tetramethylammonium hydroxide (TMAH) and NaOH, mixtures with more cycles than linear chains were obtained. Signal integration in ^29^Si NMR indicates 33 and 34% linear chains for both initiators. In contrast, only the product obtained from AROP using NaOH as the initiator shows a high-molecular-weight polymer in GPC analysis. Due to these results, we chose NaOH as a second promising initiator.

As a next step, AROP of cycles with TBPH as initiator was done at four different temperatures to determine the reaction conditions that form the highest molecular weight polymer. ^29^Si NMR shows at least 84% chains for all reaction temperatures (Figure 5a). However, only the GPC chromatogram for the AROP at 100 °C shows some high molecular weight polymer (Figure 5b). This may be due to the thermal instability of the TBPH.^38^ Since the TBPH concentration for these experiments was rather high (10 mol %), a molecular weight of only 4600 g mol^–1^ was expected. Reducing the initiator amount should increase the molecular weight of the polymer. We conducted AROP in the presence of 1 and 0.1 mol % at 80 °C. Despite the lower amount of initiator used, ^29^Si NMR shows a higher cycle content than the polymerization with 10 mol % TBPH (Table 4). Moreover, the molecular weight determined by ^29^Si NMR end group analysis decreases with decreasing initiator amount. GPC shows similar results for the cycle-to-chain ratio as ^29^Si NMR. Reducing the amount of TBPH used resulted in more cycles formed. For 0.1 mol % TBPH, GPC revealed 82% low molecular weight product. However, in contrast to ^29^Si NMR, the GPC shows a higher molecular weight when using less TBPH initiator.

**Figure 5:**
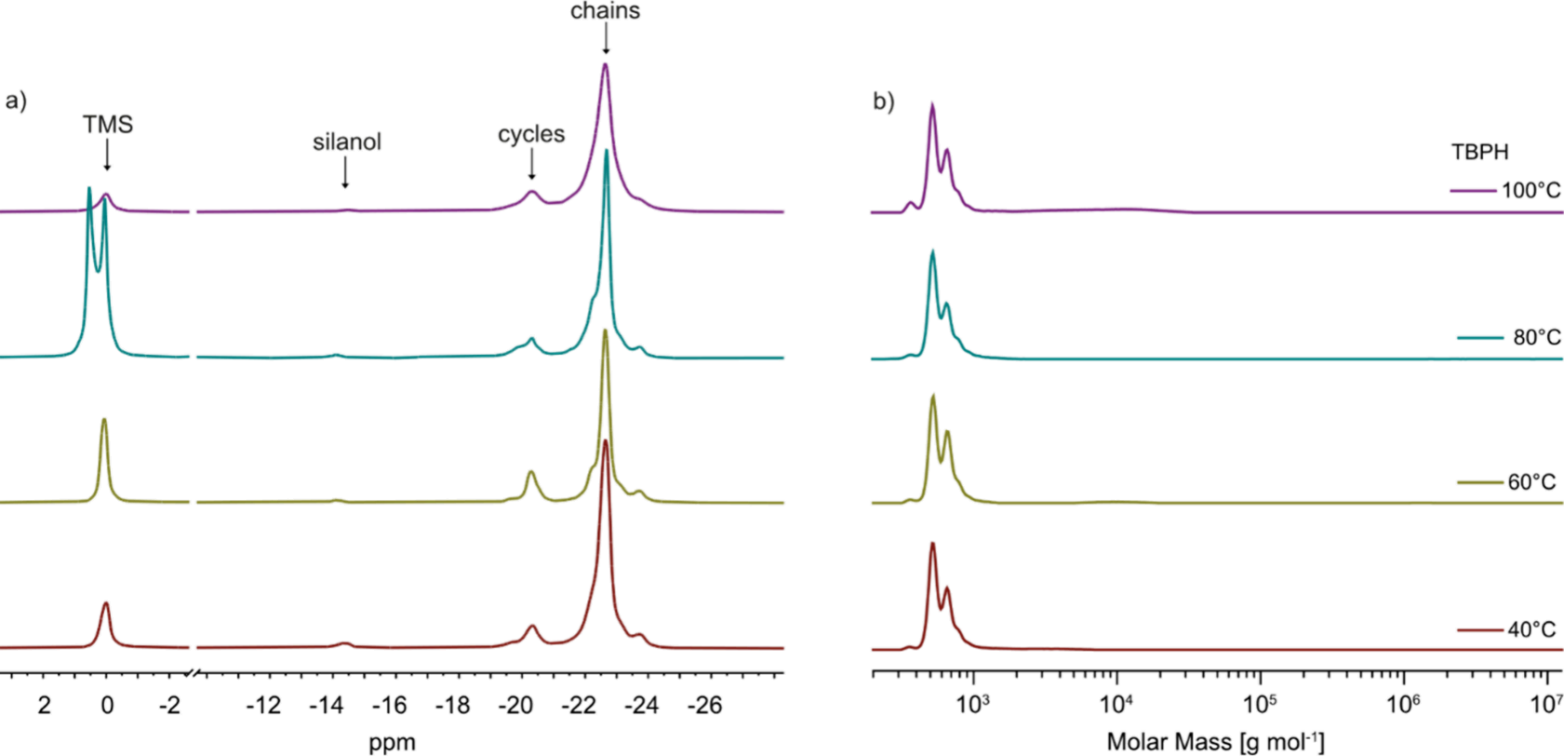
^29^Si NMR spectra (a) and GPC chromatograms (b) of the products obtained by the solvent-free AROP reaction of **D**_**4**_ **CN** with 10 mol % TBPH as the initiator at different temperatures yielded mixtures with varying ratios of chains to cycles. Only at 100 °C was some high-molecular-weight polymers obtained, as can be seen in the GPC chromatograms.

**Table 4:** AROP of **D**_**4**_ **CN** Using TBPH at Different Temperatures and in Different Concentrations^a^

				^**29**^ **Si NMR**		**GPC**			
	*T* [°C]	TBPH [mol]	monomer [mol]	cycles [%]	*M*_n_ [g mol^–1^]	chains: cycles	*M*_n_ [g mol^–1^]	*M*_w_ [g mol^–1^]	*Đ*
**P**_**CN**_ **–OH**	40	0.212	2.12	11	10,000	0:100	640	1000	1.57
**P**_**CN**_ **–OH**	60	0.212	2.12	16	24,000	12:88	9200	13,000	2.10
**P**_**CN**_ **–OH**	80	0.212	2.12	12	44,000	0:100	580	610	1.06
**P**_**CN**_ **–OH**	80	0.021	2.12	26	20,000	55:45	11,000	25,000	2.20
**P**_**CN**_ **–OH**	80	0.002	2.12	79	5600	18:82	32,000	48,000	1.51
**P**_**CN**_ **–OH**	100	0.212	2.12	14	19,000	27:73	8200	13,000	1.56

a The characteristics of the products obtained were analyzed by ^29^Si NMR and GPC.

We can conclude that TBPH easily opens the cyclic species even at temperatures as low as 40 °C. However, high temperatures are needed to polymerize the opened cycles to high molecular weight polymers. Using a smaller amount of initiator results in a higher molecular weight of the polymer and a higher proportion of low molecular weight product.

Additionally, the NaOH initiator was screened to determine the optimal reaction temperature for the AROP of cycles (Table 5). ^29^Si NMR shows an increase in the linear chain content when the temperature increases from 40 to 80 °C (Figure 6a), with a maximum content of linear chains at 80 °C (88%). However, a further increase in the temperature to 100 °C decreases the amount of chains formed. Additionally, the peak signal for the chains in the ^29^Si NMR spectrum broadened. The GPC chromatogram supports the presence of a high molar mass product in all reaction mixtures, except for the one conducted at 40 °C (Figure 6b). A polymer with a high molecular weight was only formed in reasonable amounts above 60 °C. A reaction temperature of 80 °C showed the highest proportion of high molecular weight products (76%). These findings led to the conclusion that the best reaction temperature for NaOH as an initiator for AROP of cycles is 80 °C.

**Figure 6:**
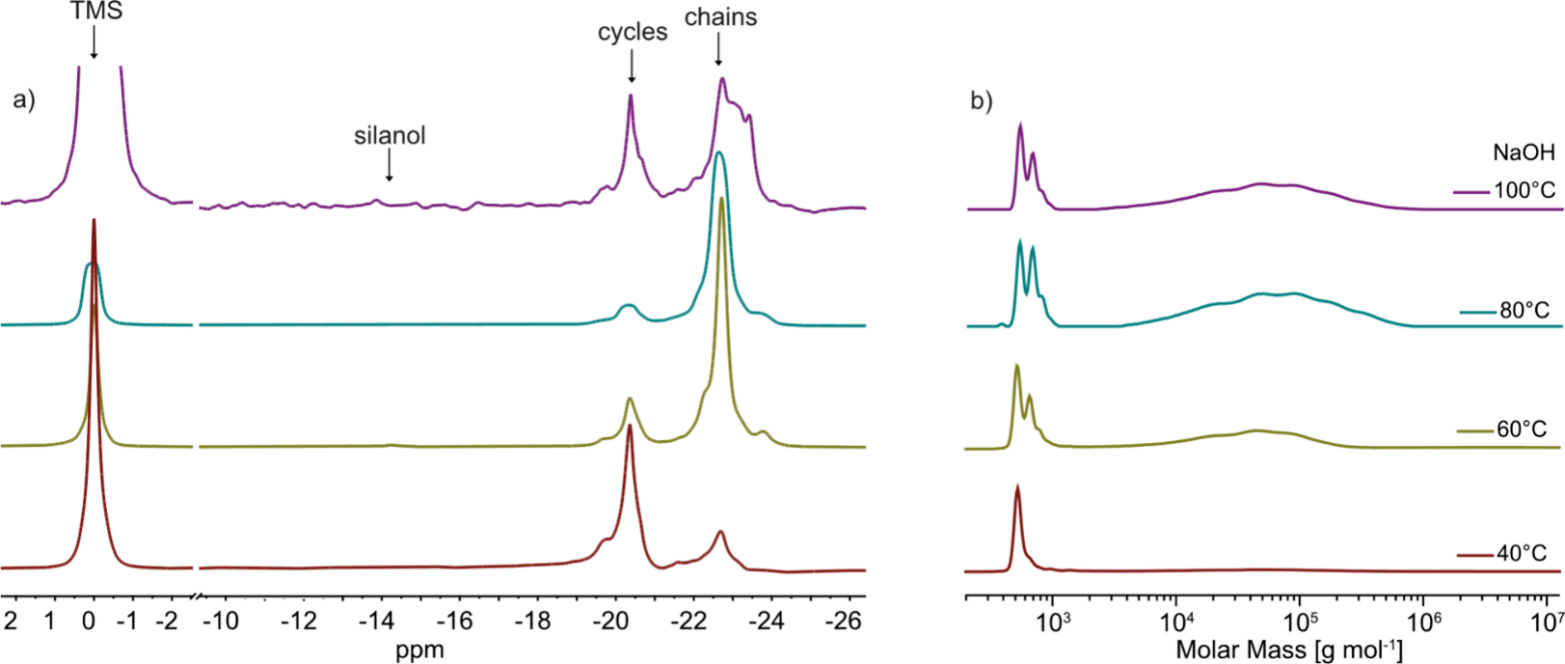
^29^Si NMR spectra (a) and GPC chromatograms (b) of the products obtained by the solvent-free AROP reaction of **D**_**4**_ **CN** with NaOH as the initiator at different temperatures gave mixtures with different ratios of chains to cycles. A high-molecular-weight product was obtained at reaction temperatures above 60 °C, as observed by GPC.

**Table 5:** AROP of **D**_**4**_ **CN** Using NaOH at Different Temperatures and in Different Concentrations^a^

				^**29**^ **Si NMR**		**GPC**			
	*T* [°C]	NaOH [mol]	monomer [mol]	cycles [%]	*M*_n_ [g mol^–1^]	chains: cycles	*M*_n_ [g mol^–1^]	*M*_w_ [g mol^–1^]	*Đ*
**PCN–OH**	40	0.20	2.12	66	61,000	23:77	31,000	85,000	2.72
**PCN–OH**	60	0.21	2.13	18	30,000	57:43	26,000	55,000	2.10
**PCN–OH**	80	0.21	2.13	12		76:24	25,000	102,000	4.04
**PCN–OH**	100	0.20	1.97	31		75:25	27,000	85,000	3.13
**PCN–OH**	100	0.02	1.97	17		75:25	26,000	135	5.16

a The characteristics of the products obtained were analyzed by ^29^Si NMR and GPC.

NaOH and TBPH were found to be the best initiators for AROP of **D**_**4**_ **CN**. TBPH is highly active in opening the **D**_**4**_ **CN** to short oligomeric chains at temperatures as low as 40 °C. However, to get a high molecular weight polymer, temperatures above 80 °C are needed. On the other hand, NaOH can polymerize **D**_**4**_ **CN** at 80 °C and gives 76% high molecular weight product. However, the workup of these reactions is tedious, making this synthetic strategy less attractive than the hydrolysis–condensation reaction.

### 2.3 Postpolymerization End-Functionalization

Regardless of the synthesis used for **P**_**CN**_ **–OH**, the polymer chains have silanol end groups. While such groups can, in principle, be used in a condensation reaction in the presence of a tin catalyst to produce a cross-linked network, these reactions are less effective than with the nonpolar silanol-terminated polydimethylsiloxane. The nitrile groups can coordinate the tin catalyst, rendering it inactive.^39^ Our next task was, therefore, to introduce other functional end groups, such as amine or vinyl, which can be used in more efficient cross-linking reactions.

To introduce amino end groups, the **P**_**CN**_ **–OH** was reacted with **2**, as shown in Scheme 1c.^32,40^ The extent of end-functionalization was determined by ^29^Si NMR (Figure 7). The silanol end group and the aminopropyl end group give distinct signals, which can be integrated and allow for quantification of the end-functionalization degree. The silanol signal at −14.5 ppm vanished, while a new signal for the silicon modified with aminopropyl at 9.1 ppm appeared, confirming a complete consumption of the silanol end groups and, therefore, an efficient end-functionalization. Additionally, we observe four new signals in the ^1^H NMR spectrum (Figure 8), which can be assigned to the protons of the aminopropyl end group. The protons of the amino group are not visible due to the fast exchange process with deuterated chloroform.^41^ Using the integrals of the proton signals of the aminopropyl groups and the ones from the cyanopropyl side chains in the ^1^H NMR gives the same degree of polymerization as observed in the ^29^Si NMR. This confirms that the aminopropyl groups are actually attached to the chain ends and not just forming dimers, which would show up as a lower degree of polymerization in the ^1^H NMR. This is also supported by diffusion NMR (Figure S1) where all the amino propyl end group signals appear. Unfortunately, GPC of this polymer cannot be measured because the aminopropyl end groups interact with the GPC column. In FT-IR (Figure S4), two major band shifts can be observed. At 1670 and 1540 cm^–1^ N–H scissoring bands are visible, the band at 1540 cm^–1^ is coupled with the C–N stretch. The vanishing band of **P**_**CN**_ **–OH** at 1680 cm^–1^ is the bending vibration of absorbed water. The band at 1063 cm^–1^ of **P**_**CN**_ **–OH** decreases to a shoulder in **P**_**CN**_ **-NH**_**2**_ and the band at 1040 cm^–1^ vanishes completely for **P**_**CN**_ **-NH**_**2**_. The band at 1063 cm^–1^ corresponds to the asymmetric Si–O–Si stretch in the chain. The red-shifted band at 1040 cm^–1^ corresponds to the asymmetric Si–O–Si stretch at the end of the chain.^42^ In **P**_**CN**_ **-NH**_**2**_ a new band at 1012 cm^–1^ is observed, corresponding to the asymmetric Si–O–Si stretch of the last repeating unit and the end group.

**Figure 7:**
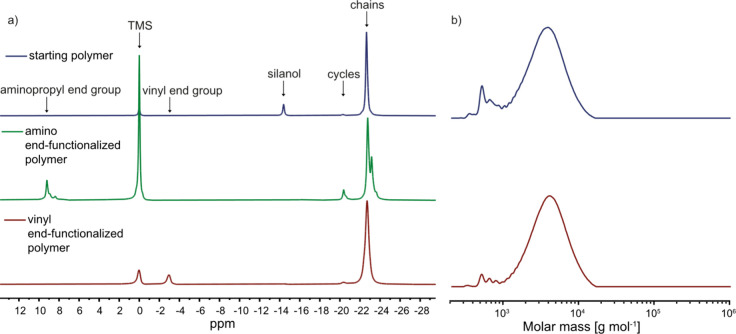
^29^Si NMR spectra of **P**_**CN**_ **–OH** (top), **P**_**CN**_ **-NH**_**2**_ (middle), and **P**_**CN**_ **-V** (bottom) (a). The signal at −14.5 ppm vanished for both end-functionalized polymers, and new signals at 9.5 or −3 ppm, respectively, appeared in the ^29^Si NMR spectrum of **P**_**CN**_ **-NH**_**2**_ and **P**_**CN**_ **-V**. GPC chromatograms of **P**_**CN**_ **–OH** and **P**_**CN**_ **-V** show a slight increase in the molecular weight for **P**_**CN**_ **-V** and no change in the polydispersity index (b). GPC **P**_**CN**_ **-NH**_**2**_ could not be measured due to interaction with the column.

**Figure 8:**
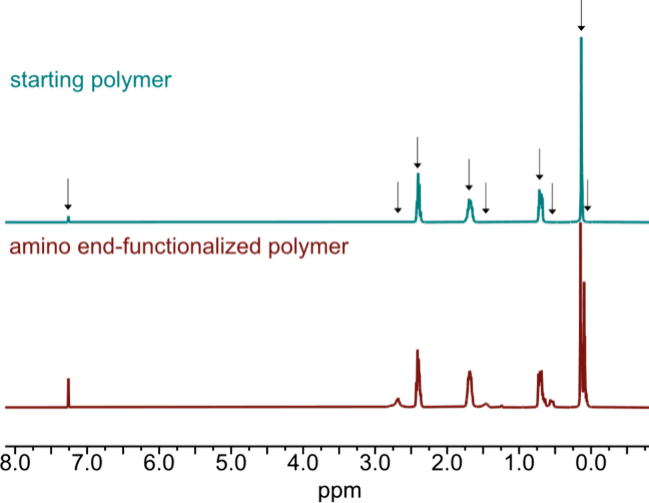
^1^H NMR spectra of **P**_**CN**_ **–OH** (top) and **P**_**CN**_ **-NH**_**2**_ (bottom). Four new signals at 2.7, 1.5, 0.5, and 0.1 ppm appeared for the end-functionalized polymer and correspond to the aminopropyl end group.

Vinyl end groups are also very useful for cross-linking, as a thiol–ene click reaction can be employed. Therefore, **P**_**CN**_ **–OH** with two different chain lengths was reacted with vinyldimethylchlorosilane (**3**) according to Scheme 1d. The ^29^Si NMR spectra and GPC chromatograms of the starting polymer **P**_**CN**_ **–OH** and the vinyl end-functionalized one can be seen in Figure 7. The degree of end-functionalization was determined by ^29^Si NMR. The silanol signal at −14.5 ppm nearly vanished, while a new signal for the vinyl end groups at −3 ppm appeared. Their integration gave an end-functionalization degree of 92% (Figure 7). GPC revealed a slight increase in the hydrodynamic volume for the end-functionalized polymer, likely due to the slight increase in the mass caused by the introduction of two vinyldimethylsiloxy groups. Importantly, this postpolymerization end-functionalization did not result in the cleavage of the polymer chains and did not generate any additional cycles (see Table 6). In FT-IR (Figure S4) the band at 1063 cm^–1^ of **P**_**CN**_ **–OH** decreases to a shoulder in **P**_**CN**_ **-V** and the band at 1040 cm^–1^ vanishes completely for **P**_**CN**_ **-V**. The band at 1063 cm^–1^ corresponds to the asymmetric Si–O–Si stretch in the chain. The red-shifted band at 1040 cm^–1^ corresponds to the asymmetric Si–O–Si stretch at the end of the chain.^42^ In **P**_**CN**_ **-V** a new band at 1012 cm^–1^ is observed, corresponding to the asymmetric Si–O–Si stretch of the last repeating unit and the end group.

**Table 6:** Characterization of the Product Obtained by Postpolymerization End-Functionalization of **P_CN_–OH** Using ^29^Si NMR End Group Analysis and GPC

		^**29**^ **Si NMR**			**GPC**			
**polymer**	*n*_RU’s_	cycles [%]	*M*_n_ [g mol^–1^]	end-functionalization [%]	chains: cycles	*M*_n_ [g mol^–1^]	*M*_w_ [g mol^–1^]	*Đ*
**P**_**CN**_ **-NH**_**2**_	9	8	1300	100	92:8			
**P**_**CN**_ **-V**	18	3	2700	91	96:4	3900	5300	1.36
**P**_**CN**_ **-V**	27	2	5000	92	98:2	7700	12,000	1.58

The successful synthesis of **P**_**CN**_ with amino and vinyl end groups represents a significant milestone in achieving polar networks with a defined network density. Such polymers can be used in highly efficient cross-linking reactions, for instance, amine with epoxy and isocyanate or vinyl with thiol, allowing the synthesis of elastomers with improved mechanical properties.

### 2.4 Cross-Linking to Elastomer

The end groups on **P**_**CN**_ can be used for cross-linking. Vinyl end groups are commonly used in thiol–ene click UV light-initiated reactions, enabling fast and reliable cross-linking.^43,44^ We cross-linked **P**_**CN**_ **-V** with pentaerythritol tetrakis­(3-mercaptopropionate), a tetrafunctional cross-linker in the presence of 2,2-dimethoxy-2-phenylacetophenone (DMPA) photoinitiator and achieved soft and highly stretchable elastomers (Figure 9a,b). Amine groups can react with epoxy groups in the presence of a tin catalyst, such as dibutyltin dilaurate (DBTL). We cross-linked **P**_**CN**_ **-NH**_**2**_ with 2,4,6,8-tetramethyl-2,4,6,8-tetrakis­({3-[(oxiran-2-yl)­methoxy]­propyl})-1,3,5,7,2,4,6,8-tetraoxatetrasilocane in the presence of DBTL and achieved soft and stretchable elastomers (Figure 9c,d).

**Figure 9:**
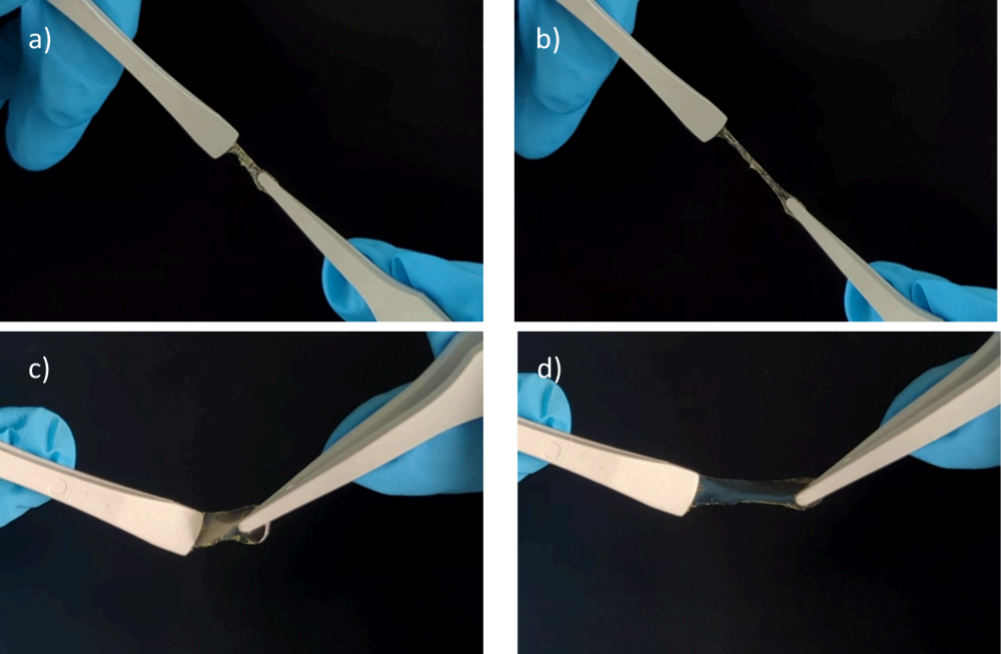
Cross-linked elastomers are soft and elastic. **P**_**CN**_ **-V** cross-linked with tetrafunctional thiol by thiol–ene addition in the presence of DMPA, unstretched (a) and stretched (b). **P**_**CN**_ **-NH**_**2**_ cross-linked with tetrakisepoxy cyclosiloxane in the presence of DBTL, unstretched (c) and stretched (d).

The polymers and the resulting elastomeric materials are characterized by thermogravimetric analysis (TGA, Figure S5) and are thermally stable up to 250 °C. **P**_**CN**_ **-NH**_**2**_ starts to degrade at 300 °C. However, the elastomer **P**_**CN**_ **-NH**_**2**_ **CL** is stable up to 320 °C. **P**_**CN**_ **-V** is stable up to 345 °C and the cross-linked elastomer **P**_**CN**_ **-V CL** is stable up to 365 °C, which is the same temperature as for the starting polymer **P**_**CN**_ **–OH**. Additionally, DSC measurements were conducted on all polymers and the resulting elastomers to determine their transitions temperatures (Figure S6). All polymers exhibited a *T*_g_, which was between −65 and −55 °C. The lowest *T*_g_ of −65 °C was observed for **P**_**CN**_ **-V**, which increased to −60 °C after cross-linking. The polysiloxane with silanol end groups has a *T*_g_ of −58 °C. End-functionalization with aminopropyl groups slightly increases the *T*_g_ to −55 °C, which stays the same for the cross-linked elastomer.

The successful cross-linking of both **P**_**CN**_ **-V** and **P**_**CN**_ **-NH**_**2**_ supports the successful end-functionalization of **P**_**CN**_ **–OH**. It is also a milestone toward polar polysiloxane networks with controlled network density. Future work will focus on the impact of molar mass and dispersity on the mechanical and electromechanical properties of elastomers synthesized using polar polysiloxanes with defined end groups for cross-linking.

## 3 Conclusions

In this study, we developed a synthetic strategy for polysiloxanes modified with 3-cyanopropyl side groups and defined vinyl or aminopropyl end groups. To achieve this, two different strategies were followed. First, the hydrolysis–condensation of cyanopropylmethyldichlorsilane under different conditions was explored. We have found that chlorinated solvents tend to produce short chains with silanol end groups, whereas nonchlorinated solvents favor the formation of cycles. When toluene is used as a solvent, the reaction mixture contains 50% cycles and 50% polymer, whereby the cycles are better soluble in toluene than the polymer. Notably, high molecular weight polymers were obtained only under solvent-free conditions, with the best results obtained when 2 equiv of water were used. Polymers with a molecular weight of 14 kg mol^–1^ and only 11% cycles formed. The cycles can be extracted from the polymer using toluene. The synthesis has been scaled up, starting with 1 kg of cyanopropylmethyldichlorosilane, which will facilitate its commercialization.

The second strategy involved the anionic ring-opening polymerization (AROP) of 3-cyanopropylmethyltetracyclosiloxane using different initiators and conditions. Only AROP with sodium hydroxide at 80 °C yielded a polymer with a molecular weight of 25 kg mol^–1^ and 24% cycles. Both strategies yielded polymers with terminal silanol groups, which were used for subsequent postpolymerization modifications.

Two different functional end groups, aminopropyl and vinyl end groups, were introduced via these silanol moieties to enable subsequent cross-linking, demonstrating the versatility of this synthetic route. Cyanopropyl-modified polysiloxanes with controlled and defined end groups offer a robust platform for the synthesis of solvent-free, processable, high dielectric permittivity elastomers with potential applications in actuators, sensors, energy storage, and light-emitting capacitive devices.

## 4 Experimental Section

Unless otherwise stated, all chemicals were reagent grade and used without further purification. Chlorodimethylvinylsilane and dibutyltindilaurate were purchased from ABCR. Tetrabutylphosphonium hydroxide (40 wt % in H_2_O) and 2,4,6,8-tetramethyl-2,4,6,8-tetrakis­({3-[(oxiran-2-yl)­methoxy]­propyl})-1,3,5,7,2,4,6,8-tetraoxatetrasiloxane were purchased from Fischer Scientific. 3-Cyanopropylmethyldichlorsilane was purchased from Gelest. Lithium hydroxide (anhydrous) and pentaerythritol tetrakis (3-mercaptopropionate) were purchased from Merck. Sodium bicarbonate, acetonitrile, dichloromethane, chloroform, tetrahydrofuran, toluene, sodium hydroxide, potassium hydroxide, and tetramethylammonium hydroxide (25 wt % in H_2_O) were purchased from VWR.

### 4.1 Hydrolysis–Condensation of 3-Cyanopropylmethyldichlorsilane in Different Solvents (Acetonitrile, Chloroform, DCM, THF) Using Sodium Bicarbonate

In a 250 mL round flask equipped with a magnetic stirrer, sodium bicarbonate (36.75 g, 438 × 10^–3^ mol) was suspended in solvent (70 mL). A solution of **1**, (10 mL, 63.1 × 10^–3^ mol) in solvent (20 mL) was added dropwise to the vigorously stirred suspension. The addition was performed slowly due to the formation of CO_2_, which led to the foaming of the reaction mixture. After the addition was complete, a condenser was installed, and the mixture was refluxed overnight (Table 7). The reaction mixture was allowed to cool to room temperature and then filtered. The residue was washed with a large amount of dichloromethane. The organic phase was concentrated using a rotary evaporator and subsequently washed three times with Milli-Q water. The organic phase was concentrated using a rotary evaporator under reduced pressure. Finally, the product was dried overnight in the vacuum oven at 100 °C to obtain a slightly yellow oily product. ^1^H NMR (CDCl_3_, δ, ppm): 2.4 (m, 2H, CH_2_–CN), 1.7 (m, 2H, CH_2_–CH_2_–CN), 0.7 (m, 2H, Si-CH_2_), 0.1 (m, 3H, Si-CH_3_). ^13^C NMR (CDCl_3_, δ, ppm): 119.7 (CN), 20.4 (CH_2_–CN), 19.7 (CH_2_–CH_2_–CN), 16.4 (Si-CH_2_), −0.7 (Si-CH_3_). ^29^Si NMR (TMS, δ, ppm): −9.8 (Si–OH, dimer), −14.5 (Si–OH), −20.3 (−Si­(CH_2_)­(CH_2_)_3_CN–O–, in cycles), −22.6 (−Si­(CH_2_)­(CH_2_)_3_CN–O–, in linear chains).

**Table 7:** Solvent and Reaction Temperature Used for the Hydrolysis–Condensation Reaction of **1** to **P_CN_–OH** with 7 equiv NaHCO_3_ and the Supporting Information for Their Characterization

solvent	*T* [°C]	^1^H NMR	^13^C NMR	^28^Si NMR	GPC
CH_3_CN	85	Figure S7	Figure S8	Figure S9	Figure S10
CHCl_3_	75	Figure S11	Figure S12	Figure S13	Figure S14
DCM	45	Figure S15	Figure S16	Figure S17	Figure S18
THF	80	Figure S19	Figure S20	Figure S21	Figure S22

### 4.2 Hydrolysis–Condensation of 3-Cyanopropylmethyldichlorsilane in Toluene Using Sodium Bicarbonate

In a 1 L three-neck flask equipped with a magnetic stirrer, dropping funnel, dean-stark apparatus, and condenser, sodium bicarbonate (147 g, 1.75 mol) was suspended in toluene (300 mL). A solution of **1** (45.5 mL, 287.31 × 10^–3^ mol) in toluene (75 mL) was added dropwise to the vigorously stirred suspension. The addition was done slowly due to the formation of CO_2_, which caused the foaming of the reaction mixture. After the addition was complete, the mixture was heated to 110 °C. The reaction mixture was stirred at 110 °C, while the water was removed by azeotropic distillation. The reaction was followed by the measurement of the amount of water produced through azeotropic distillation. After 66 h, 9 mL of water was collected in the dean-stark apparatus. The reaction mixture was allowed to cool to room temperature and then filtered. The residue was washed with a large amount of toluene to yield the first fraction. Subsequently, the first fraction was washed with Milli-Q water three times. The organic phase was concentrated using a rotary evaporator under reduced pressure. Finally, the product was dried overnight in a vacuum oven at 100 °C to obtain a slightly yellow, oily final product. ^1^H NMR (CDCl_3_, δ, ppm): 2.4 (m, 2H, CH_2_–CN), 1.7 (m, 2H, CH_2_-CH_2_–CN), 0.7 (m, 2H, Si-CH_2_), 0.1 (m, 3H, Si-CH_3_) (Figure S23). ^13^C NMR (CDCl_3_, δ, ppm): 119.6 (CN), 20.3 (CH_2_–CN), 19.6 (CH_2_–CH_2_–CN), 16.3 (Si-CH_2_), −0.8 (Si-CH_3_) (Figure S24). ^29^Si NMR (TMS, δ, ppm): 17 h reaction time (Figure S25), and 66 h reaction time (Figure S27): −9.8 (Si–OH, dimer), −14.5 (Si–OH), −20.3 (-Si­(CH_2_)­(CH_2_)_3_CN–O–, in cycles), −22.7 (−Si­(CH_2_)­(CH_2_)_3_CN–O–, in linear chains). Molar mass determination by GPC calibrated with PS standard and by ^29^Si NMR after 17 h reaction time (Figure S26) and 66 h reaction time Figure S28).

The remaining residue on the filter paper was washed with a large amount of dichloromethane to give the second fraction. Subsequently, the second fraction was washed with Milli-Q water three times. The organic phase was concentrated using a rotary evaporator under reduced pressure. Finally, the product was dried overnight in a vacuum oven at 100 °C to obtain a slightly yellow, viscous liquid. ^1^H NMR (CDCl_3_, δ, ppm): 2.4 (m, 2H, CH_2_–CN), 1.7 (m, 2H, CH_2_–CH_2_–CN), 0.7 (m, 2H, Si-CH_2_), 0.1 (m, 3H, Si-CH_3_) (Figure S29). ^13^C NMR (CDCl_3_, δ, ppm): 119.7 (CN), 20.5 (CH_2_–CN), 19.8 (CH_2_–CH_2_–CN), 16.9­(Si-CH_2_), −0.2 (Si-CH_3_) (Figure S30). ^29^Si NMR (TMS, δ, ppm): 17 h reaction time (Figure S31), and 66 h reaction time (Figure S33), −14.4 (Si–OH), −22.7 (−Si­(CH_2_)­(CH_2_)_3_CN–O–, in linear chains). Molar mass determination by GPC calibrated with PS standard, 17 h reaction time (Figure S32) and 66 h reaction time (Figure S34).

### 4.3 Solvent-Free Synthesis Using Sodium Bicarbonate

In a 250 mL flask equipped with a magnetic stirrer, sodium bicarbonate was added (Table 6). **1** (10 mL, 63.1 × 10^–3^ mol) was added dropwise to the flask. After the addition was completed, a condenser was installed, and the mixture was heated to 110 °C and stirred overnight. The reaction mixture was allowed to cool to room temperature. The mixture was filtered, and the residue was washed with a large amount of dichloromethane. The organic phase was concentrated at the rotary evaporator to reduce the volume of dichloromethane. Subsequently, the organic phase was washed with Milli-Q water three times. The organic phase was concentrated using a rotary evaporator under reduced pressure. Finally, the product was dried overnight in a vacuum oven at 100 °C to obtain the slightly yellow oily product. ^1^H NMR (CDCl_3_, δ, ppm): 2.4 (m, 2H, CH_2_–CN), 1.7 (m, 2H, CH_2_–CH_2_–CN), 0.7 (m, 2H, Si-CH_2_), 0.2 (m, 3H, Si-CH_3_). ^13^C NMR (CDCl_3_, δ, ppm): 119.7 (CN), 20.5 (CH_2_-CN), 19.8 (CH_2_–CH_2_–CN), 16.9 (Si-CH_2_), −0.2 (Si-CH_3_). ^29^Si NMR (TMS, δ, ppm): −14.5 (Si–OH), −20.3 (−Si­(CH_2_)­(CH_2_)_3_CN–O–, in cycles), −22.6 (−Si­(CH_2_)­(CH_2_)_3_CN–O–, in linear chains).

### 4.4 Solvent-Free Synthesis Using Water

In a 250 mL flask equipped with a magnetic stirrer and a condenser, **1** (10 mL, 63.1 × 10^–3^ mol) was added. Mili-Q water was added dropwise (Table 8). Evolving hydrogen chloride was quenched in gas-washing flasks filled with aqueous sodium hydroxide solution. After the addition was completed, the mixture was heated to 100 °C and stirred overnight. The reaction mixture was allowed to cool to room temperature. The mixture was diluted with dichloromethane. Subsequently, the organic phase was washed with Milli-Q water three times. The organic phase was concentrated using a rotary evaporator under reduced pressure. Finally, the product was dried overnight in a vacuum oven at 100 °C to obtain a slightly yellow oily product. ^1^H NMR (CDCl_3_, δ, ppm): 2.4 (m, 2H, CH_2_–CN), 1.7 (m, 2H, CH_2_–CH_2_–CN), 0.7 (m, 2H, Si-CH_2_), 0.2 (m, 3H, Si-CH_3_). ^13^C NMR (CDCl_3_, δ, ppm): 119.7 (CN), 20.5 (CH_2_–CN), 19.8 (CH_2_-CH_2_–CN), 16.9 (Si-CH_2_), −0.2 (Si-CH_3_). ^29^Si NMR (TMS, δ, ppm): −14.5 (Si–OH), −20.3 (−Si­(CH_2_)­(CH_2_)_3_CN–O–, in cycles), −22.6 (−Si­(CH_2_)­(CH_2_)_3_CN–O–, in linear chains).

**Table 8:** Solvent-Free Hydrolysis–Condensation of (3-Cyanopropyl)­methyldichlorosilane Using Different Amounts of Initiator (I) NaHCO_3_ or H_2_O for Synthesis and the Supporting Information for Their Characterization

initiator	*I* [equiv]	*n* [mol]	*I* [g]	*V* [mL]	^1^H, ^13^C, ^29^Si NMR and GPC
NaHCO_3_	7	438 × 10^–3^	36.75		Figures S35–S38
NaHCO_3_	3	188 × 10^–3^	15.75		Figures S39–S42
H_2_O	2	126 × 10^–3^	2.27	2.27	Figures S43–S46
H_2_O	1	63.1 × 10^–3^	1.14	1.14	Figures S47–S50

### 4.5 General Procedure to Separate Cycles from Chains

The crude polymer was extracted 3 times with toluene. The toluene phase was decanted, and the polymer was dried by rotary evaporator. ^29^Si NMR of reaction mixture Figure S51; of purified polymer Figure S52; and of extractables in toluene Figure S53.

### 4.6 AROP of Cycles with Inorganic Bases as the Initiator

In a 25 mL round-bottomed flask equipped with a magnetic stirrer and a septum, aqueous solutions of base (1 wt %, 0.197 × 10^–3^ mol, Table 9) were added. The flask and the aqueous solution of the base were flame-dried. **D**_**4**_ **CN** (1 g, 1.97 × 10^–3^ mol) prepared by hydrosilylation was added inside the glovebox. The mixture was heated and stirred overnight. After cooling to room temperature, the crude was dissolved in dichloromethane and washed with Mili-Q water until neutral.

**Table 9:** Anionic Ring-Opening Polymerization of **D**_**4**_ **CN** Using Different Initiators and Temperatures and the Supporting Information for Their Characterization

initiator	I [mg]	I, 1 wt % sol [mL]	*T* [°C]	^1^H, ^13^C, ^29^Si NMR, and GPC
LiOH	4.71	0.47	40	Figures S54–S57
NaOH	7.87	0.79	40	Figures S58–S61
NaOH	8.50	0.85	60	Figures S62–S65
NaOH	8.50	0.85	80	Figures S66–S69
NaOH	7.87	0.79	100	Figures S70–S73
KOH	11.04	1.10	40	Figures S74–S77

^1^H NMR (CDCl_3_, δ, ppm): 2.4 (m, 2H, CH_2_–CN), 1.7 (m, 2H, CH_2_–CH_2_–CN), 0.7 (m, 2H, Si-CH_2_), 0.1 (m, 3H, Si-CH_3_). ^13^C NMR (CDCl_3_, δ, ppm): 119.7 (CN), 20.4 (CH_2_–CN), 19.7 (CH_2_–CH_2_–CN), 16.4 (Si-CH_2_), −0.7 (Si-CH_3_). ^29^Si NMR (TMS, δ, ppm): −14.5 (Si–OH), −20.4 (−Si­(CH_2_)­(CH_2_)_3_CN–O–, in cycles), −22.7 (−Si­(CH_2_)­(CH_2_)_3_CN–O–, in linear chains).

### 4.7 AROP of Cycles with TMAH or TBPH as the Initiator

In a 25 mL round-bottomed flask equipped with a magnetic stirrer and a septum, TMAH (25% in H_2_O) or TBPH (40% in H_2_O) (0.197 × 10^–3^ mol, Table 10) was added. The aqueous solution was dried by azeotropic distillation twice with 0.5 mL of dry benzene. **D**_**4**_ **CN** (1 g, 1.97 × 10^–3^ mol) was added inside the glovebox to the base. The mixture was heated and stirred overnight. After cooling to room temperature, the crude was dissolved in dichloromethane and washed with Mili-Q water until neutral. ^1^H NMR (CDCl_3_, δ, ppm): 2.4 (m, 2H, CH_2_–CN), 1.7 (m, 2H, CH_2_–CH_2_–CN), 0.7 (m, 2H, Si-CH_2_), 0.1 (m, 3H, Si-CH_3_). ^13^C NMR (CDCl_3_, δ, ppm): 119.7 (CN), 20.5 (CH_2_–CN), 19.8 (CH_2_–CH_2_–CN), 16.9 (Si-CH_2_), −0.2 (Si-CH_3_). ^29^Si NMR (TMS, δ, ppm): −14.6 (Si–OH), −20.3 (−Si­(CH_2_)­(CH_2_)_3_CN–O–, in cycles), −22.6 (−Si­(CH_2_)­(CH_2_)_3_CN–O–, in linear chains).

**Table 10:** Anionic Ring-Opening Polymerization of **D**_**4**_ **CN** with Different Transient Bases and at Different Temperatures and the Supporting Information for Their Characterization

initiator	*I* [mg]	*I* [mL]	*T* [°C]	^1^H, ^13^C, ^29^Si NMR and GPC
TMAH	17.9	0.07	40	Figures S78–S81
TBPH	58.8	0.15	40	Figures S82–S85
TBPH	58.8	0.15	60	Figures S86–S89
TBPH	58.8	0.15	80	Figures S90–S93
TBPH	5.88	0.015	80	Figures S94–S97
TBPH	0.588	0.0015	80	Figures S98–S101
TBPH	58.8	0.15	100	Figures S102–S105

### 4.8 Aminopropyl End Group Functionalization of **P_CN_–OH**

In a 100 mL Schlenk-flask equipped with a magnetic stirrer and condenser, a mixture of cycles and **P**_**CN**_ **–OH** (1 eq, 3.01 g) was added. The setup was put under an argon atmosphere. The polymer mixture was dissolved in heptane (30 mL). 3-[(2,2-Dimethyl-1,2-azasilolidin-1-yl)­(dimethyl)­silyl]­propan-1-amine (**2**) (5.8 equiv, 0.1 mL) were added to the stirring solution. The reaction mixture was heated to 60 °C and stirred overnight. All volatiles were removed at a rotary evaporator. The crude was dissolved in dichloromethane and washed with Mili-Q water three times. The organic layer was concentrated and dried at a rotary evaporator. ^1^H NMR (CDCl_3_, δ, ppm): 2.7 (m, 4H, CH_2_–NH_2_), 2.4 (m, 20H, CH_2_–CN), 1.7 (m, 16H, CH_2_–CH_2_–CN), 1.5 (m, 4H, CH_2_–CH_2_–NH_2_), 0.7 (m, 15H, Si-CH_2_– CH_2_–CH_2_–CN), 0.5 (m, 5H, Si-CH_2_– CH_2_–CH_2_–NH_2_), 0.2 (m, 39H, Si-CH_3_) (Figure S106). ^13^C NMR (CDCl_3_, δ, ppm): 119.7 (CN), 20.5 (CH_2_–CN, CH_2_–NH_2_), 19.8 (CH_2_–CH_2_–CN, CH_2_–CH_2_–NH_2_), 16.9 (Si-CH_2_–CH_2_–CH_2_–CN), 15.4 (Si-CH_2_–CH_2_–CH_2_–NH_2_), −0.2 (Si-CH_3_ (RU)), −0.2 (Si-CH_3_ (end group)) (Figure S107). ^29^Si NMR (TMS, δ, ppm): 9.2 (Si-CH_2_–CH_2_–CH_2_–NH_2_), −20.4 (−Si­(CH_2_)­(CH_2_)_3_CN–O–, in cycles), −22.8 (−Si­(CH_2_)­(CH_2_)_3_CN–O–, in linear chains) (Figure S108).

### 4.9 Vinyl End Group Functionalization of **P_CN_–OH**

In a 100 mL Schlenk-flask equipped with a magnetic stirrer and septum, silanol-terminated polymer **P**_**CN**_ **–OH** was diluted in dichloromethane (*c* = 0.2 g/mL). Dimethylvinylchlorosilane (**3**) was added by syringe (Table 11). The reaction mixture was stirred overnight at room temperature. It was then washed with Mili-Q water three times. The crude was concentrated and dried in a rotary evaporator. Finally, the product (**P**_**CN**_ **-V**) was dried in a vacuum oven at 100 °C overnight to obtain a slightly yellow oily product. ^1^H NMR (CDCl_3_, δ, ppm): 5.75–6.05 (m, 6 H, vinyl), 2.37 (m, 38H, CH_2_–CN), 1.67 (m, 38H, CH_2_–CH_2_–CN), 0.70 (m, 38H, Si-CH_2_), 0.13 (m, 64H, Si-CH_3_). ^13^C NMR (CDCl_3_, δ, ppm): 138.62 (Si-CH=CH_2_), 132.55 (Si-CH=CH_2_), 119.67 (CN), 20.46 (CH_2_–CN), 19.77 (CH_2_–CH_2_–CN), 16.39 (Si-CH_2_), −0.69 (Si-CH_3_). ^29^Si NMR (TMS, δ, ppm): −2.98 (Si-CH=CH_2_), −9.82 (Si–O–Si dimer), −20.34 (−Si­(CH_2_)­(CH_2_)_3_CN–O–, in cycles), −22.68 (−Si­(CH_2_)­(CH_2_)_3_CN–O–, in linear chains).

**Table 11:** Reagents for Vinyl End-Functionalization and the Supporting Information for Their Characterization

**starting polymer**			**3**		^**1**^ **H,** ^**13**^ **C,** ^**29**^ **Si NMR, and GPC**
	*m* [g]	*n* [mmol]	*V* [mL]	*n* [mmol]	
**P**_**CN**_ **–OH**	2.02	0.89	0.3	2.20	Figures S109–S113
**P**_**CN**_ **–OH**	8.47	1.80	0.61	4.47	Figures S114–S118

### 4.10 Cross-linking **P_CN_-V** into Elastomer Film by Thiol–Ene Reaction

**P**_**CN**_ **-V** (0.2 g) was mixed with 43 μL pentaerythritol tetrakis­(3-mercaptopropionate) (**CL4**)­(20 v% in THF) and 45 μL DMPA (100 mg/mL THF) in a speed mixer (3500 rpm, 5 min). The film was cast on a PET substrate using doctor blading and cross-linked by exposing it to UV light for 10 min. An elastic film formed.

### 4.11 Cross-linking **P_CN_-NH_2_** into Elastomer Film with Epoxy Cross-Linker

**P**_**CN**_ **-NH**_**2**_ (0.2 g) was mixed with 15 μL 2,4,6,8-tetramethyl-2,4,6,8-tetrakis­({3-[(oxiran-2-yl)­methoxy]­propyl})-1,3,5,7,2,4,6,8-tetraoxatetrasilocane in a speed mixer (3500 rpm, 5 min). One μL dibutyltindilaurate (DBTL) was added and mixed in a speed mixer (3500 rpm, 20 s). The film was cat on a glass substrate using doctor blading and cross-linked on the heat plate at 100 °C in 10 min. An elastic film formed.

## Supplementary Material



## Notes

The Supporting Information is available free of charge at https://pubs.acs.org/doi/10.1021/acspolymersau.5c00080 and the raw data is available upon request at 10.5281/zenodo.17131977.

All NMR spectra ^1^H, ^13^C, ^29^Si spectra and the GPC chromatograms of the polymers synthesized using different conditions and reagents (PDF)

## Abbreviations

AROP: anionic ring-opening polymerization
D_4_CN: 1,3,5,7-tetramethyl-1,3,5,7-tetra­(3-cyanopropyl)­cyclotetrasiloxane
DBTL: dibutyltin dilaurate
DCM: dichlormethane
equiv: equivalents
DMPA: 2,2-dimethoxy-2-phenylacetophenone
GPC: gel permeation chromatography
NMR: nuclear magnetic resonance
P_CN_: poly­(3-cyanopropylmethyl)­siloxane
P_CN_-NH_2_: aminopropyl-terminated poly­(3-cyanopropylmethyl)­siloxane
P_CN_–OH: silanol-terminated poly­(3-cyanopropylmethyl)­siloxane
P_CN_-V: vinyl-terminated poly­(3-cyanopropylmethyl)­siloxane
PDMS: polydimethylsiloxane
TBPH: tetrabutylphosphonium hydroxide
*T*_g_: glass transition temperature
THF: tetrahydrofuran
TMAH: tetramethylammonium hydroxide
UV: ultraviolet
