# Supplementary material for: Nitrile-Functionalized Polysiloxanes with Controlled End Groups for Elastomeric Networks
Source: ACS Polym Au. 2025 Sep 22;5(5):620–32. doi: 10.1021/acspolymersau.5c00080 (PMC12511973; doi:10.1021/acspolymersau.5c00080)
Supplement: Supplementary file 1 [file lg5c00080_si_001.pdf]

Nitrile-Functionalized Polysiloxanes with Controlled End Groups for Elastomeric Network

Jana Wolf[1,2], Patrick M. Danner[1,2] and Dorina M. Opris[1,2]*

[1]Laboratory for Functional Polymers, Swiss Federal Laboratories for Materials Science and Technology (Empa), Ueberlandstr. 129, 8600 Dübendorf, Switzerland

[2]Department of Materials, ETH Zurich, Vladimir-Prelog-Weg 5, 8093 Zurich, Switzerland

Supporting information

Characterization Methods

Gel permeation chromatography (GPC) measurements in tetrahydrofuran were performed using an *Agilent* 1260 infinity on two tandem-connected mixed-bed columns (1 × PLgel 5 µm MIXED-C Guard and 2 × PLgel 5 µm MIXED-C Analytical). As a detector, a 390-MDS refractive index detector was used. The flow rate was 1 mL min^{-1} and the temperature was 35 °C. PS standards were used for calibration.

^{1}H-, ^{13}C-, and ^{29}Si-NMR spectra were recorded at 298 K on a *Bruker* Avance 400 MHz NMR spectrometer. Chemical shifts (δ) in the ^{1}H and ^{13}C NMR spectra are given in ppm relative to $CHCl_3$ (δ = 7.26 ppm and δ = 77.16 ppm). The chemical shifts (δ) in the ^{29}Si-NMR spectra are given in ppm relative to TMS (δ = 0 ppm). A 0.1 mol L^{-1} chromium(III) acetylacetonate solution in $CDCl_3$ is used as a paramagnetic relaxation agent in ^{29}Si-NMR measurements.

IR spectra were recorded in the range of 4000–600 cm^{-1} on a Bruker Tensor 27 FT-IR spectrometer equipped with an ATR setup.

AROP of cycles in the presence of LiOH and quenched with diluted acetic acid

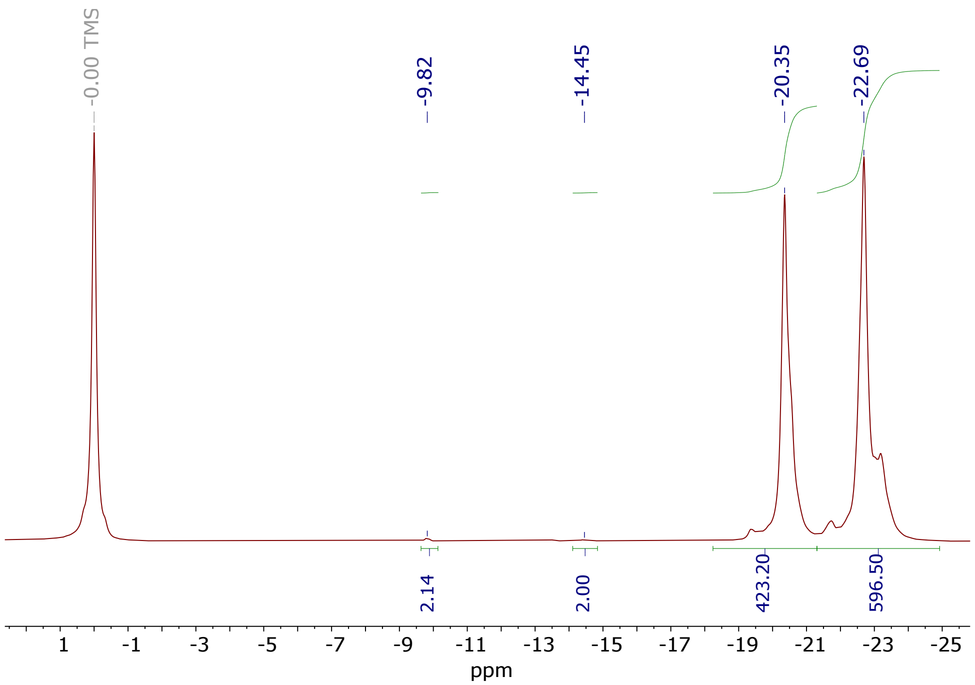

Figure S1. ^{29}NMR spectrum of $\mathbf{P_{CN}}$**-OH** obtained from AROP using LiOH. The reaction was quenched with diluted acetic acid. The spectrum was recorded with $Cr(acac)_3$ as a relaxation agent and TMS as a reference.

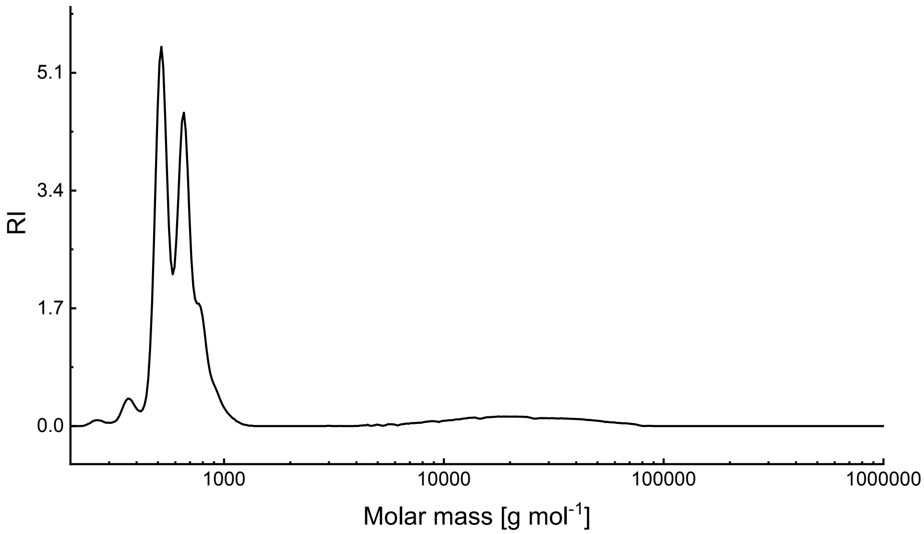

Figure S2. GPC chromatogram of $\mathbf{P_{CN}}$**-OH** obtained from AROP of cycles using LiOH and quenching with diluted acetic acid using PS standard and THF solvent.

Post-polymerization end-functionalization with cyclic silazane

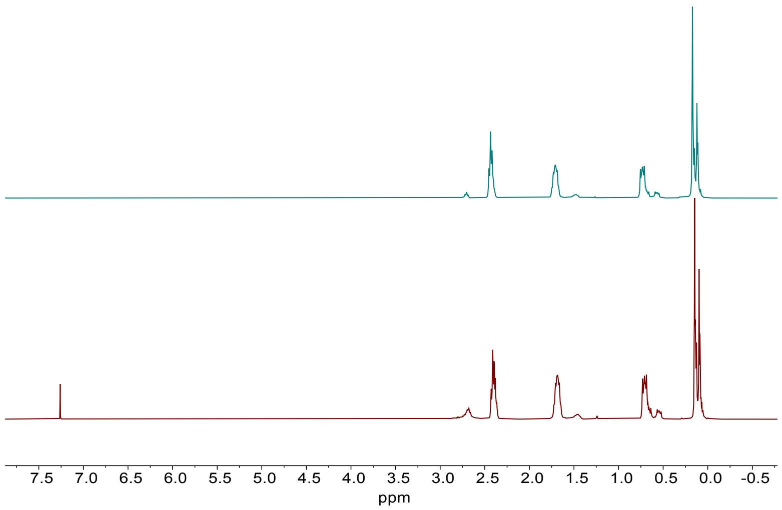

Figure S3. Diffusion-edited NMR of ($\mathbf{P_{CN}}$**-NH$_2$**) obtained from end-functionalization of short chains (top) and ^{1}H NMR of ($\mathbf{P_{CN}}$**-NH$_2$**) stacked (bottom).

Post-polymerization end-functionalization FT-IR

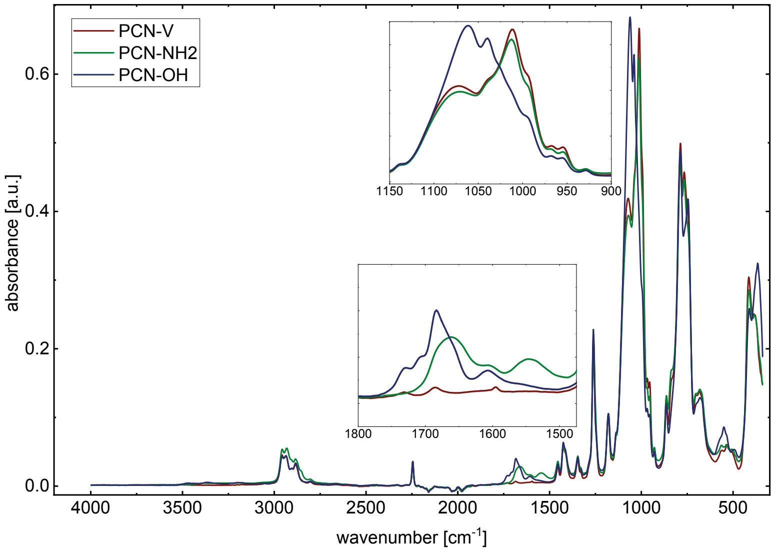

Figure S4. FT-IR of $\mathbf{P_{CN}}$**-V**, $\mathbf{P_{CN}}$**-NH$_2$** and $\mathbf{P_{CN}}$**-OH**. The enlarged wavenumber ranges show the main differences in the absorbance for the 3 polymers. Between 1800 and 1475 cm^{-1} the N-H bendings are visible

for **P_{CN}-NH$_2$** and for **P_{CN}-OH** the bending of absorbed water is visible. Between 1150 and 900 cm^{-1} different peaks for the asymmetric Si-O-Si are visible for the not end-functionalized **P_{CN}-OH** and the end-functionalized polymers (**P_{CN}-NH$_2$** and **P_{CN}-V**).

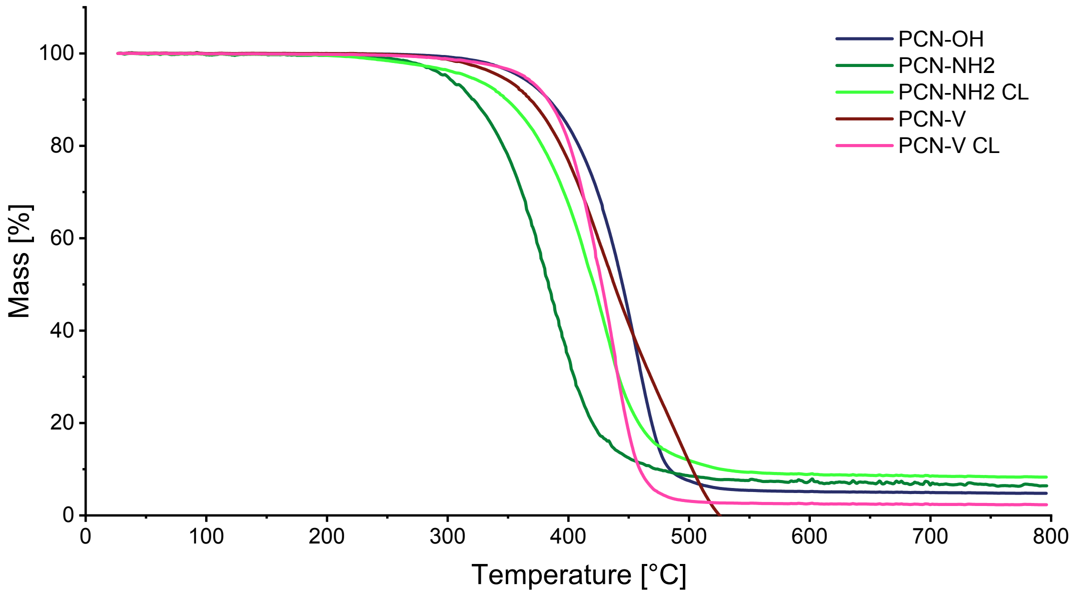

Figure S5. TGA of **P_{CN}-V**, **P_{CN}-NH$_2$** and **P_{CN}-OH**, and the cross-linked elastomers **P_{CN}-NH$_2$ CL** and **P_{CN}-V CL**. All polymers and elastomers are thermally stable up to 250 °C.

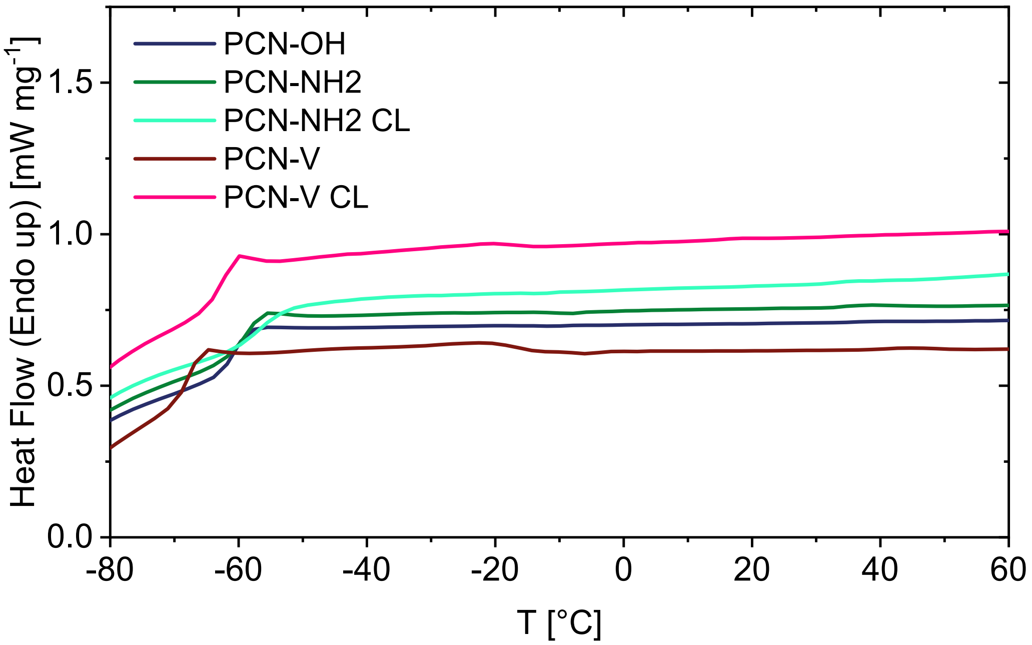

Figure S6. DSC of $\mathbf{P_{CN}}$**-V**, $\mathbf{P_{CN}}$**-NH$_2$** and $\mathbf{P_{CN}}$**-OH**, and the cross-linked elastomers $\mathbf{P_{CN}}$**-NH$_2$ CL** and $\mathbf{P_{CN}}$**-V CL**. All T_g's are observed between -65 and -55°C.

Polymerization using bicarbonate in acetonitrile

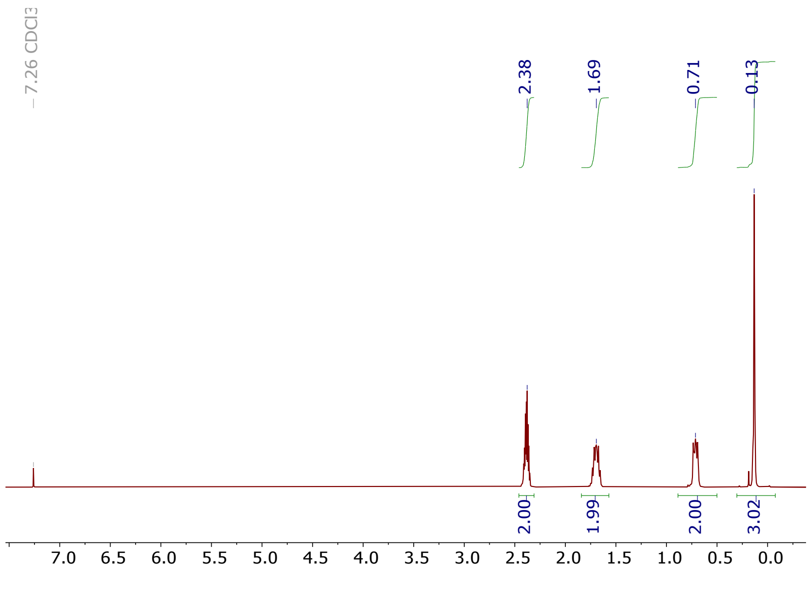

Figure S7. ^{1}H NMR spectrum in $CDCl_3$ of $\mathbf{P_{CN}}$**-OH** obtained from polymerization using bicarbonate in CH_3CN.

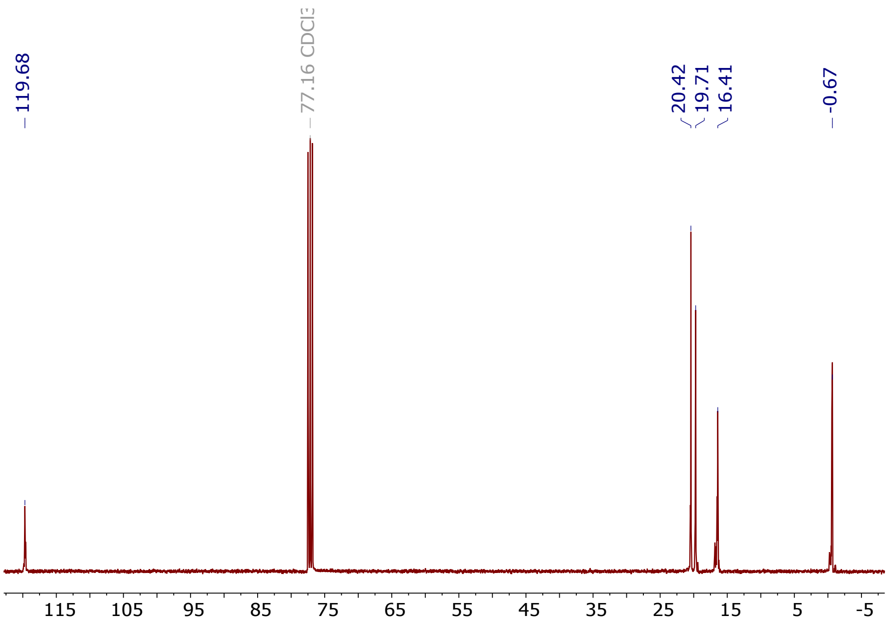

Figure S8. ^{13}C NMR spectrum in $CDCl_3$ of **P_{CN}-OH** obtained from polymerization using bicarbonate in CH_3CN.

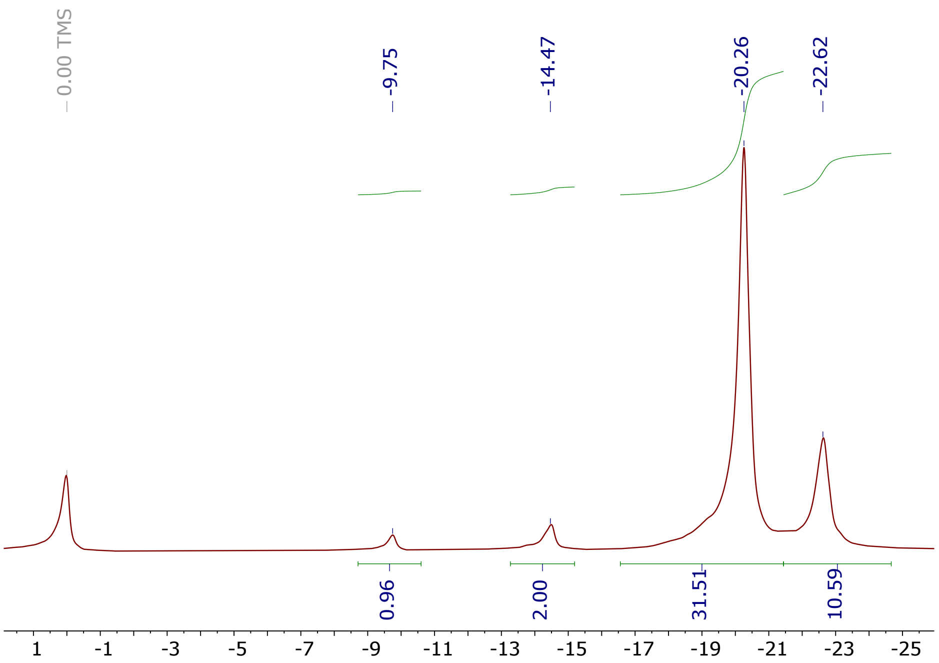

Figure S9. ^{29}NMR spectrum of **P_{CN}-OH** obtained from polymerization using bicarbonate in CH_3CN. The spectrum was recorded with $Cr(acac)_3$ as a relaxation agent and TMS as a reference.

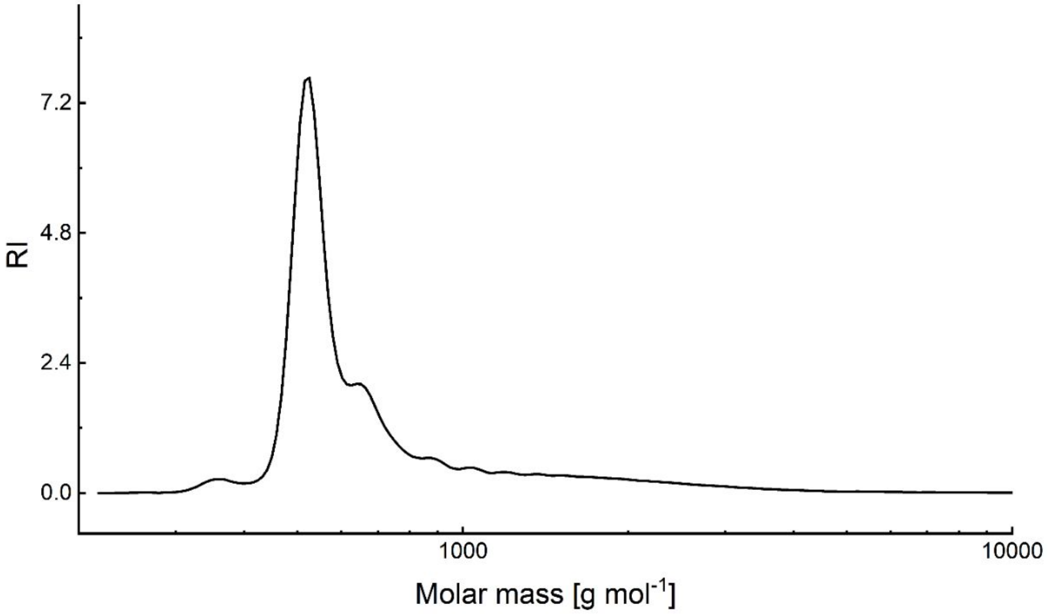

Figure S10. Molar mass determination by GPC calibrated with PS standard of **P_{CN}-OH** obtained from polymerization using bicarbonate in CH_3CN.

Polymerization using bicarbonate in chloroform

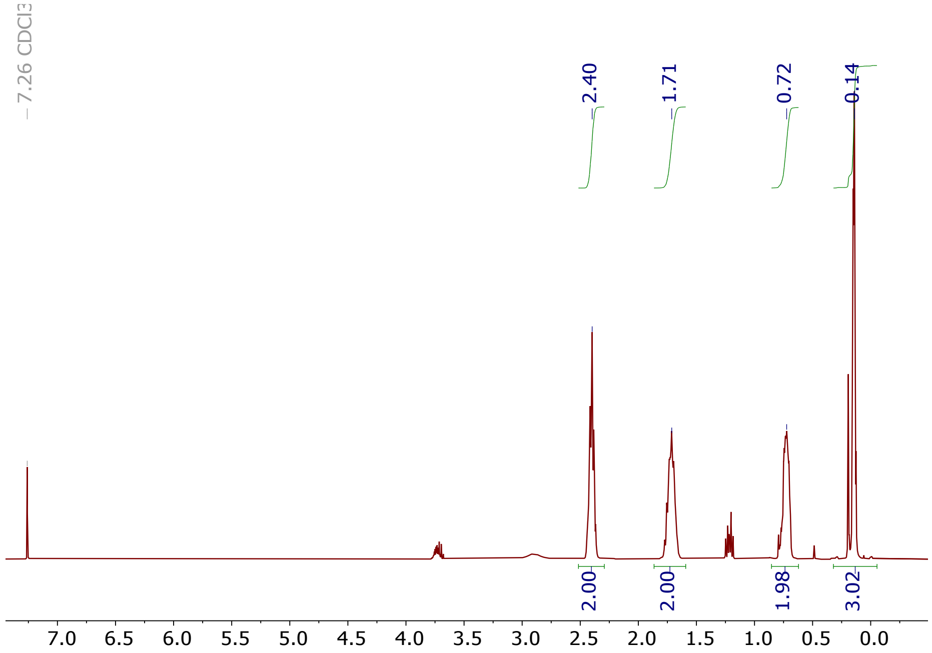

Figure S11. ^{1}H NMR spectrum in $CDCl_3$ of **P_{CN}-OH** obtained from polymerization using bicarbonate in $CHCl_3$.

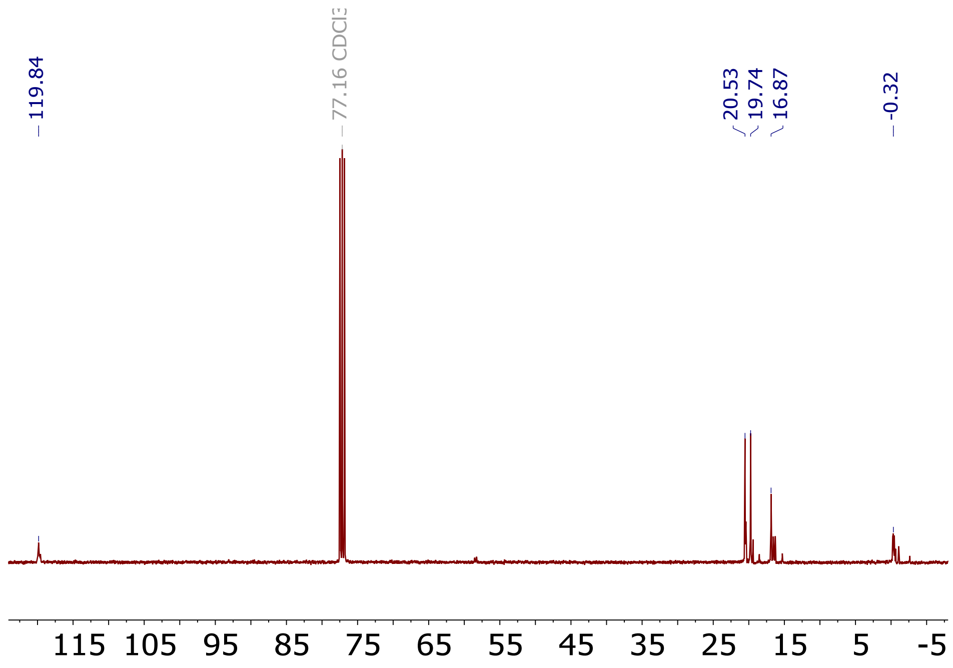

Figure S12. ^{13}C NMR spectrum in $CDCl_3$ of $\mathbf{P_{CN}}$**-OH** obtained from polymerization using bicarbonate in $CHCl_3$.

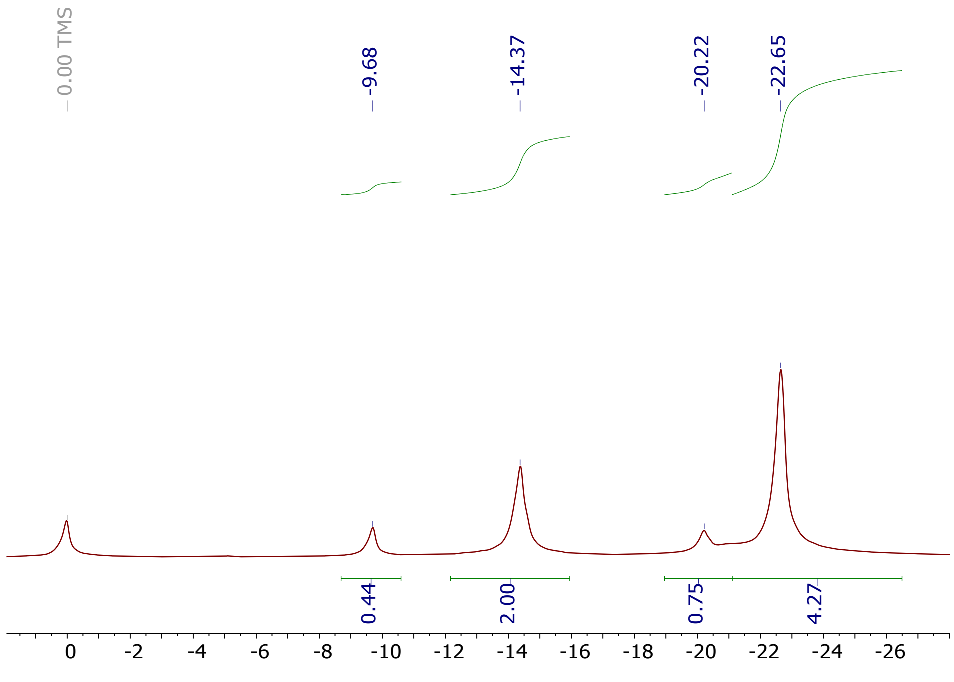

Figure S13. ^{29}Si NMR spectrum of $\mathbf{P_{CN}}$**-OH** obtained from polymerization using bicarbonate in $CHCl_3$. The NMR was recorded using $Cr(acac)_3$ as a relaxation agent and TMS as a reference.

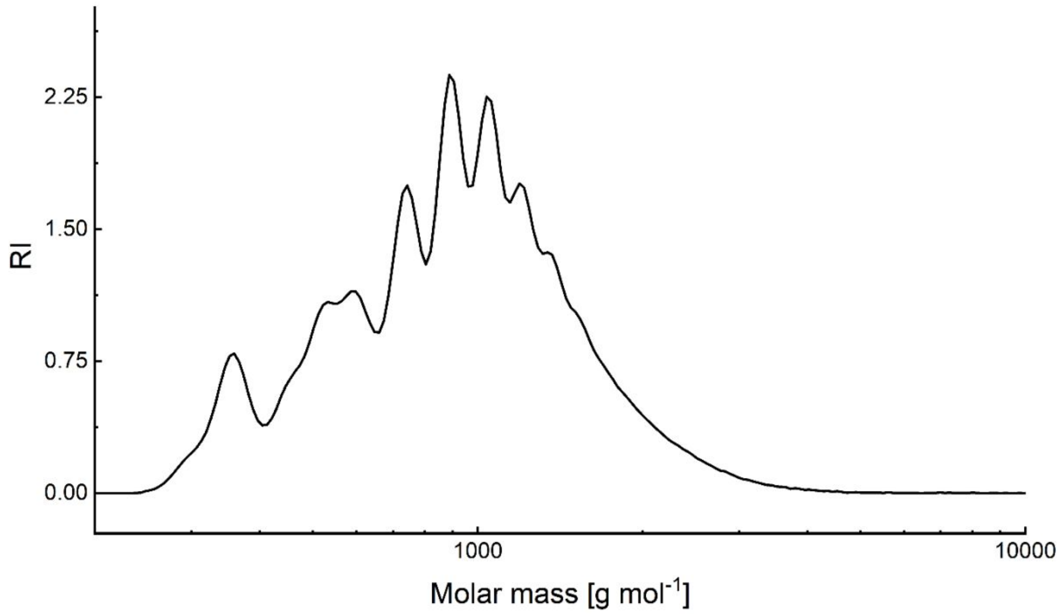

Figure S14. GPC chromatogram of $\mathbf{P_{CN}}$**-OH** obtained from the reaction using bicarbonate in $CHCl_3$ using PS standard and THF solvent.

Polymerization using bicarbonate in dichloromethane:

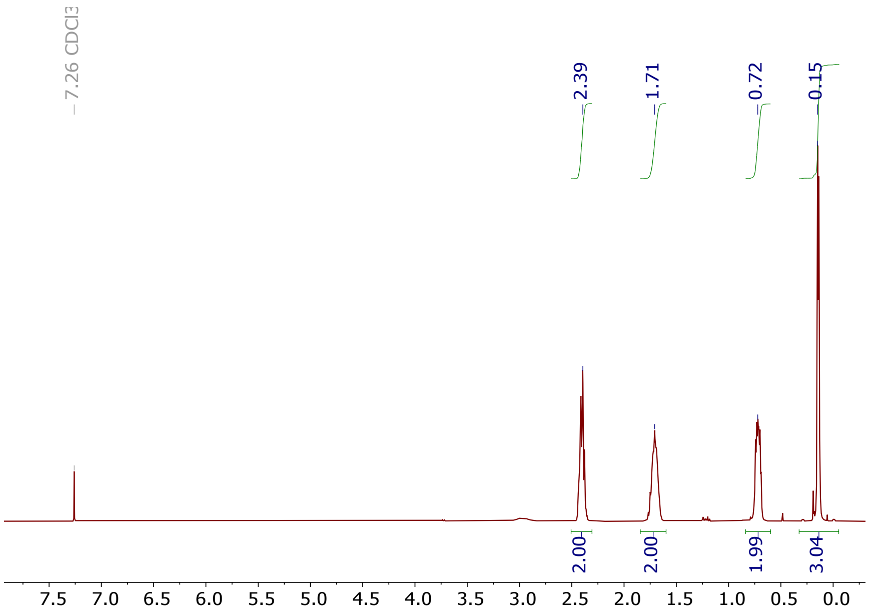

Figure S15. ^1H NMR spectrum in $CDCl_3$ of $\mathbf{P_{CN}}$**-OH** obtained from polymerization using bicarbonate in DCM.

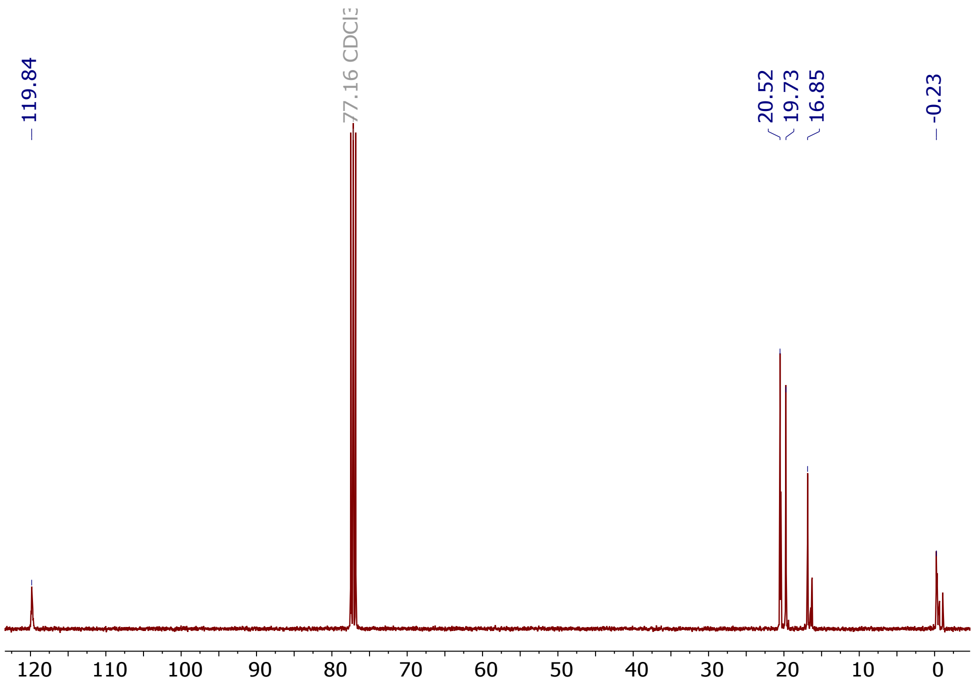

Figure S16. ^{13}C NMR spectrum in $CDCl_3$ of $\mathbf{P_{CN}}$**-OH** obtained from polymerization using bicarbonate in DCM.

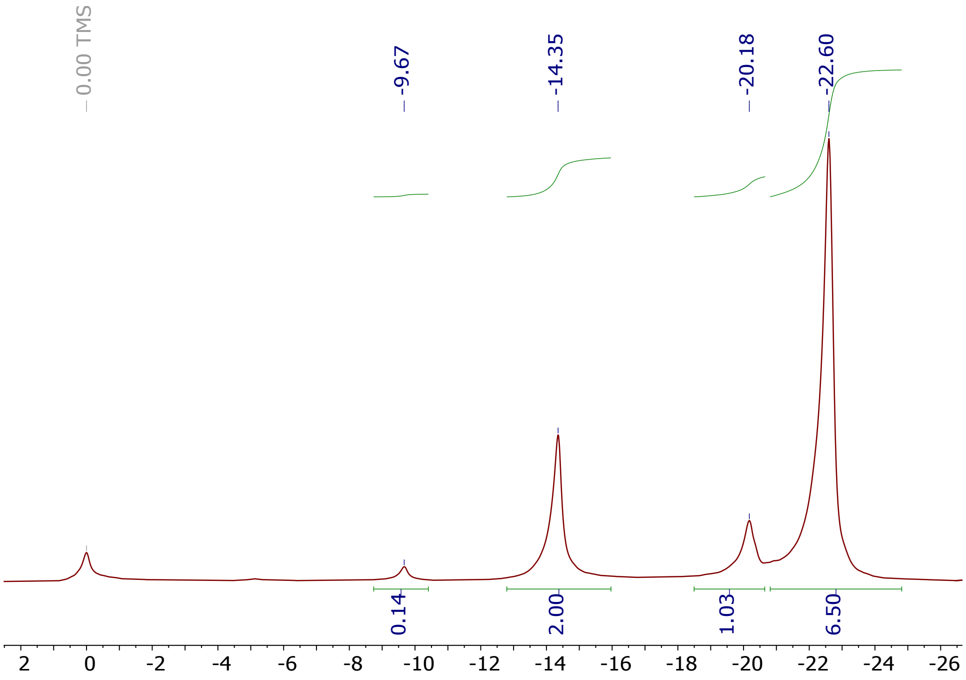

Figure S27. ^{29}Si NMR spectrum of $\mathbf{P_{CN}}$**-OH** obtained from polymerization using bicarbonate in DCM. The spectrum was recorded using $Cr(acac)_3$ as a relaxation agent and TMS as a reference.

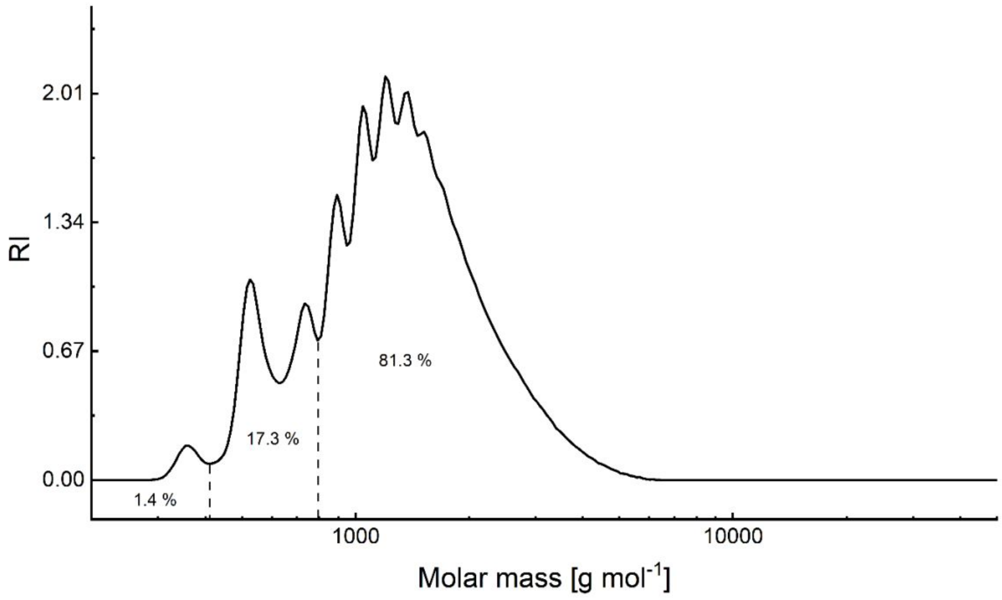

Figure S38. GPC chromatogram of **P_{CN}-OH** obtained from polymerization using bicarbonate in DCM using PS standard and THF as a solvent.

Polymerization using bicarbonate in tetrahydrofuran

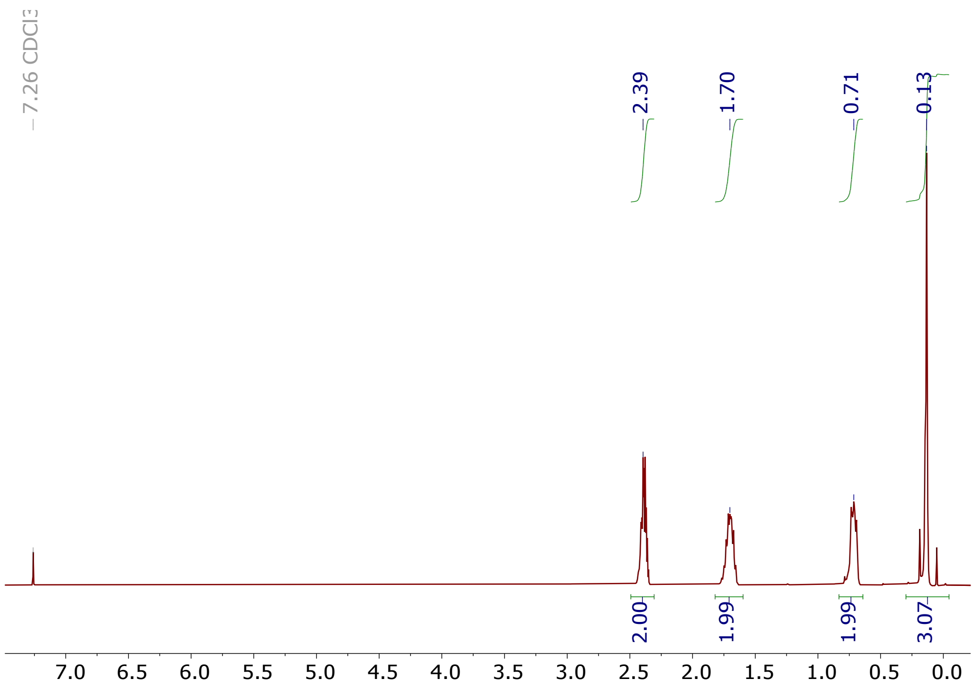

Figure S49. ^{1}H NMR spectrum in $CDCl_3$ of **P_{CN}-OH** obtained from polymerization using bicarbonate in THF.

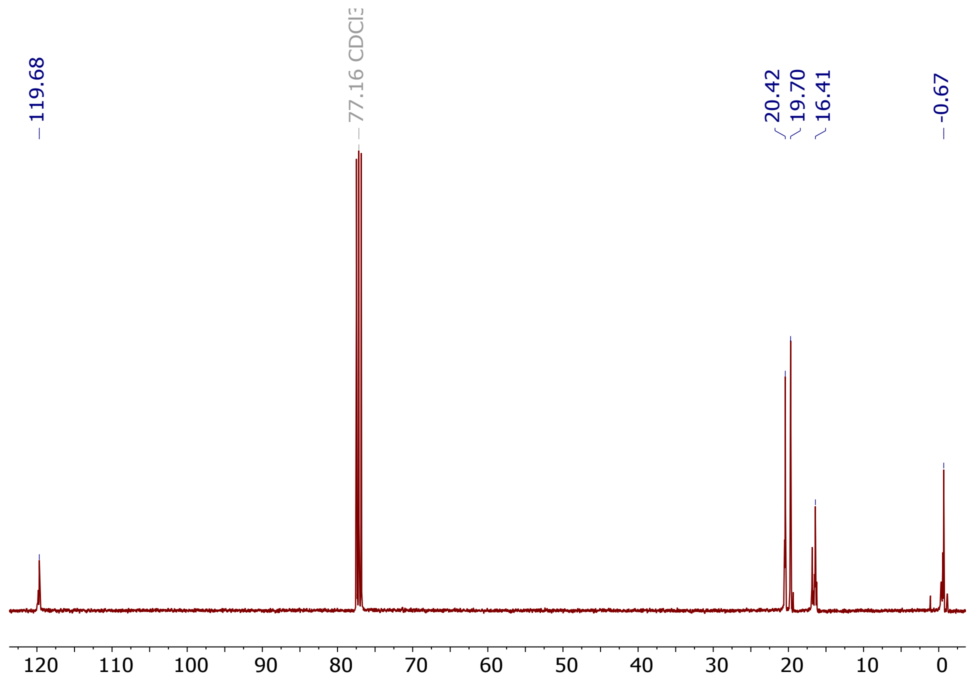

Figure S20. ^{13}C NMR spectrum in $CDCl_3$ of **P_{CN}-OH** obtained from polymerization using bicarbonate in THF.

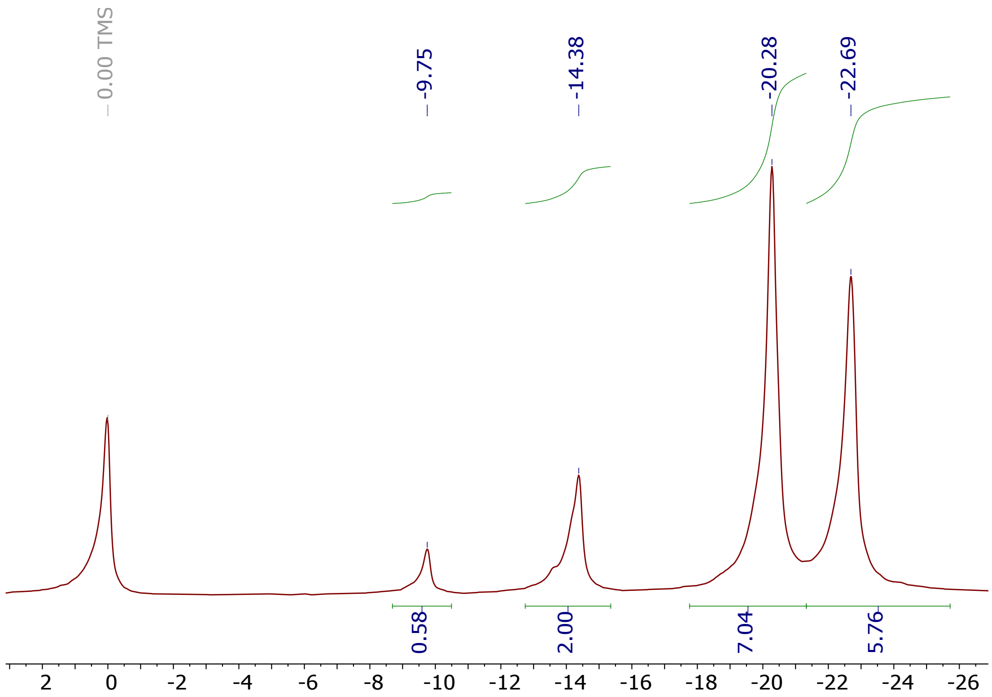

Figure S21. ^{29}Si NMR spectrum of **P_{CN}-OH** obtained from polymerization using bicarbonate in THF. The spectrum was recorded using $Cr(acac)_3$ as a relaxation agent and TMS as a reference.

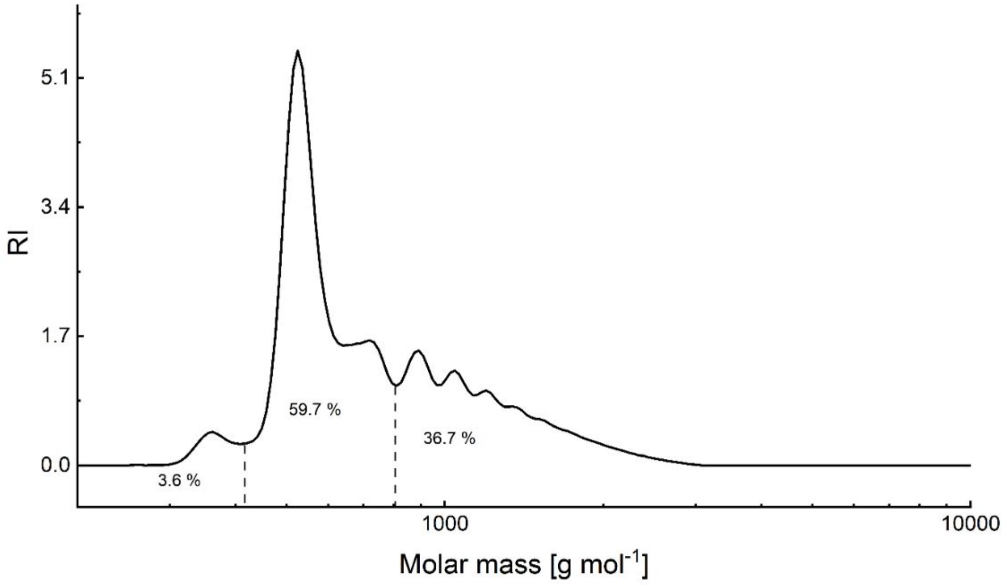

Figure S22. GPC chromatogram of $\mathbf{P_{CN}}$**-OH** obtained from polymerization using bicarbonate in THF. The GPC used tetrahydrofurane as a solvent and was calibrated with PS standard.

Reaction using bicarbonate and toluene and azeotropic distillation

Cycles:

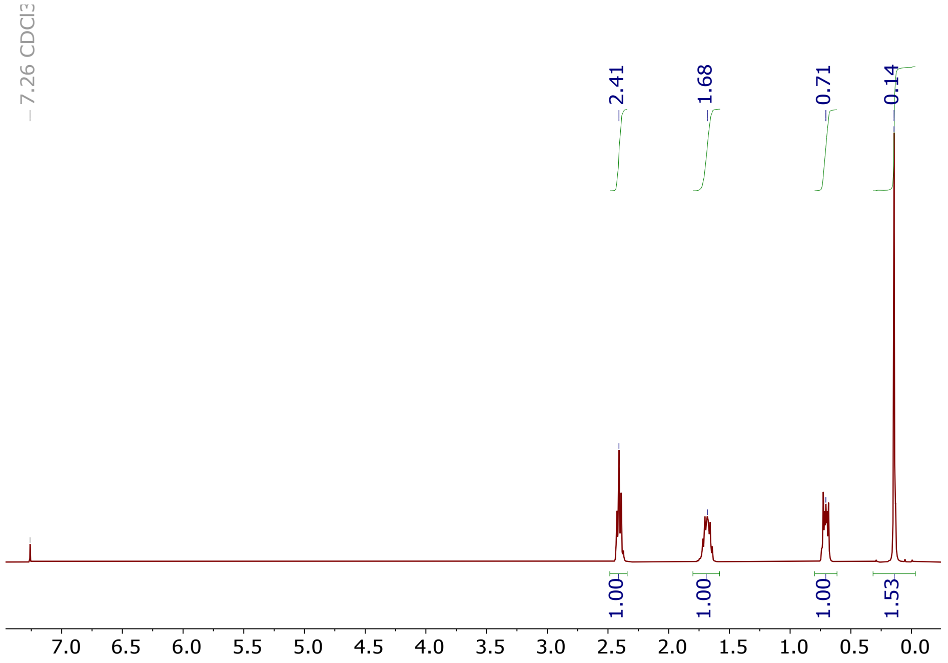

Figure S23. ^{1}H NMR spectrum in $CDCl_3$ of cyclic monomer (**M**) obtained from polymerization using bicarbonate in toluene.

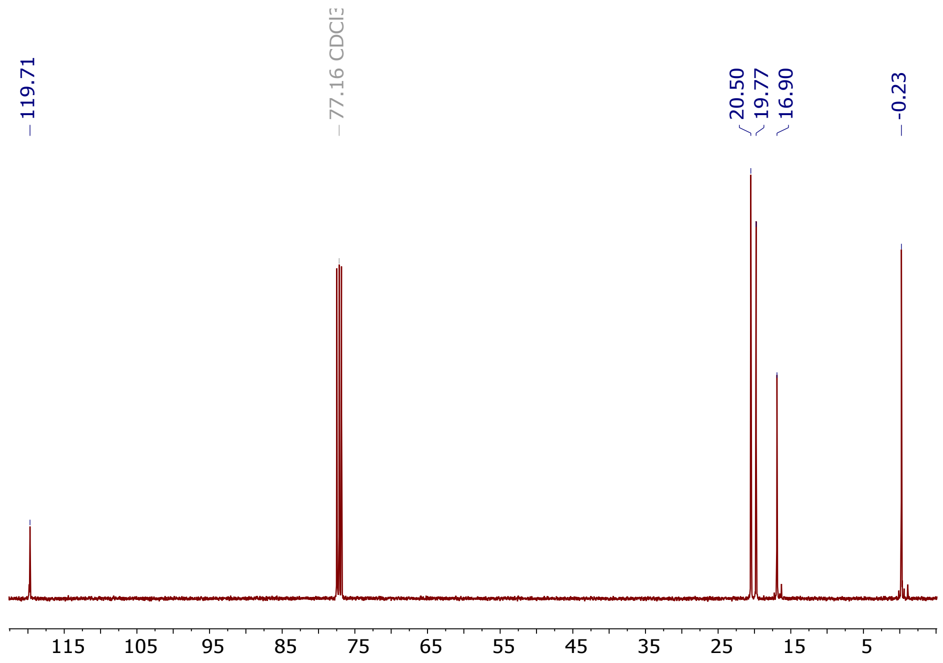

Figure S24. ^{13}C NMR spectrum in $CDCl_3$ of cyclic monomer (**M**) obtained from polymerization using bicarbonate in toluene.

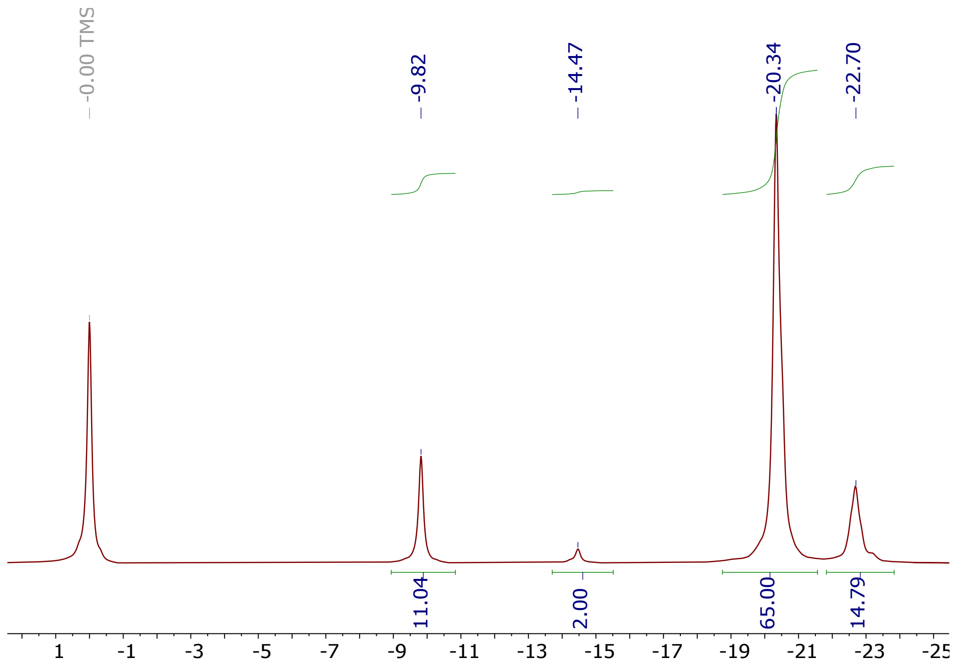

Figure S25. ^{29}Si NMR spectrum of cyclic monomer (**M**) obtained from polymerization using bicarbonate in toluene, 17 h reaction time. The spectrum was recorded using $Cr(acac)_3$ as a relaxation agent and TMS as a reference.

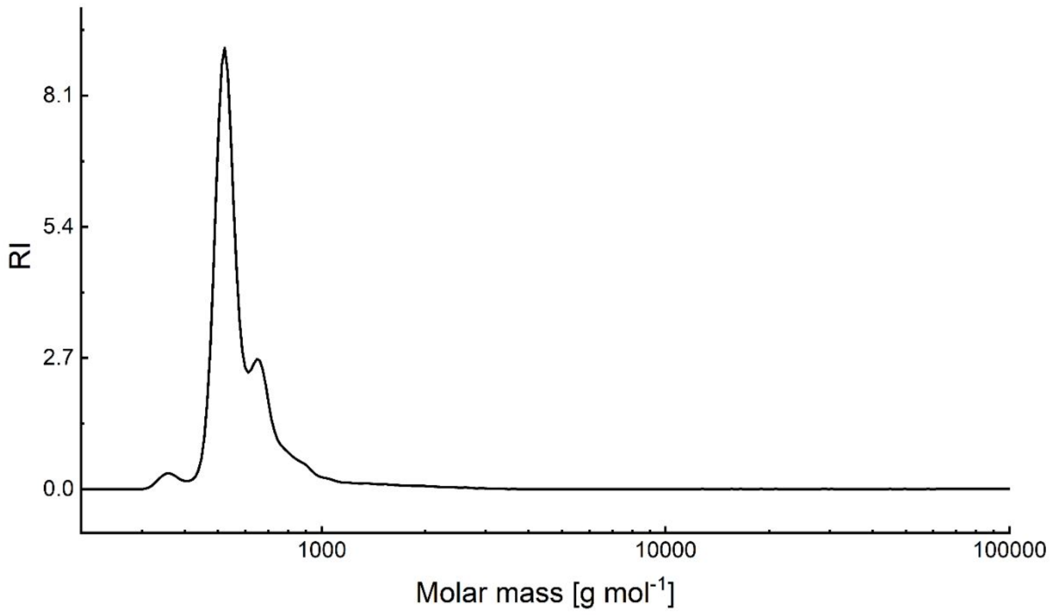

Figure S56. GPC chromatogram in tetrahydrofuran of cyclic monomer (**M**) obtained from polymerization using bicarbonate in toluene, 17 h reaction time. The GPC was calibrated with PS standard.

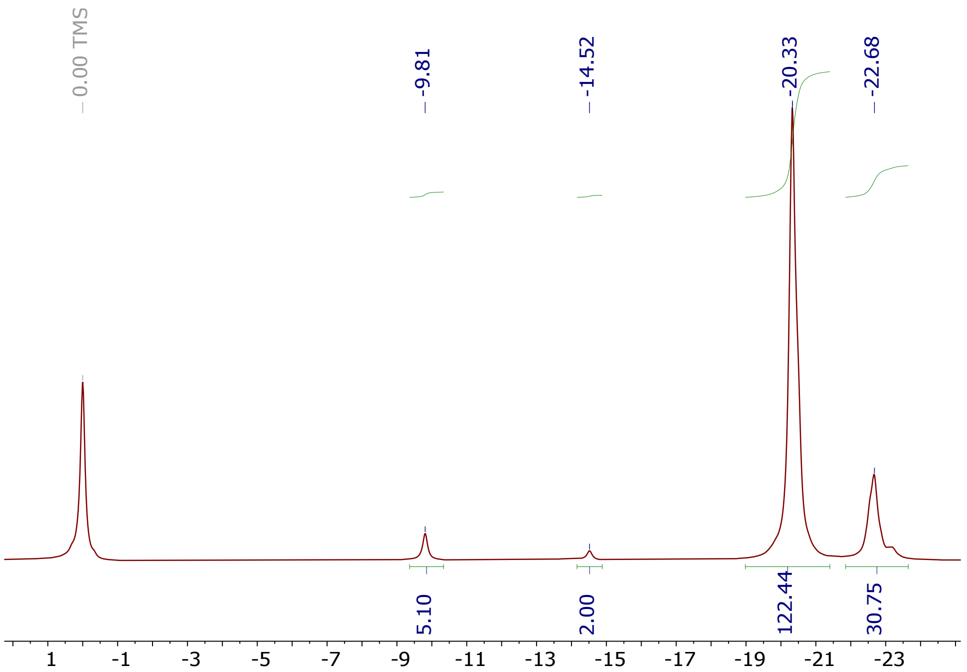

Figure S27. ^{29}Si NMR spectrum of cyclic monomer (**M**) obtained from polymerization using bicarbonate in toluene, 66 h reaction time. The spectrum was recorded using $Cr(acac)_3$ as a relaxation agent and TMS as a reference.

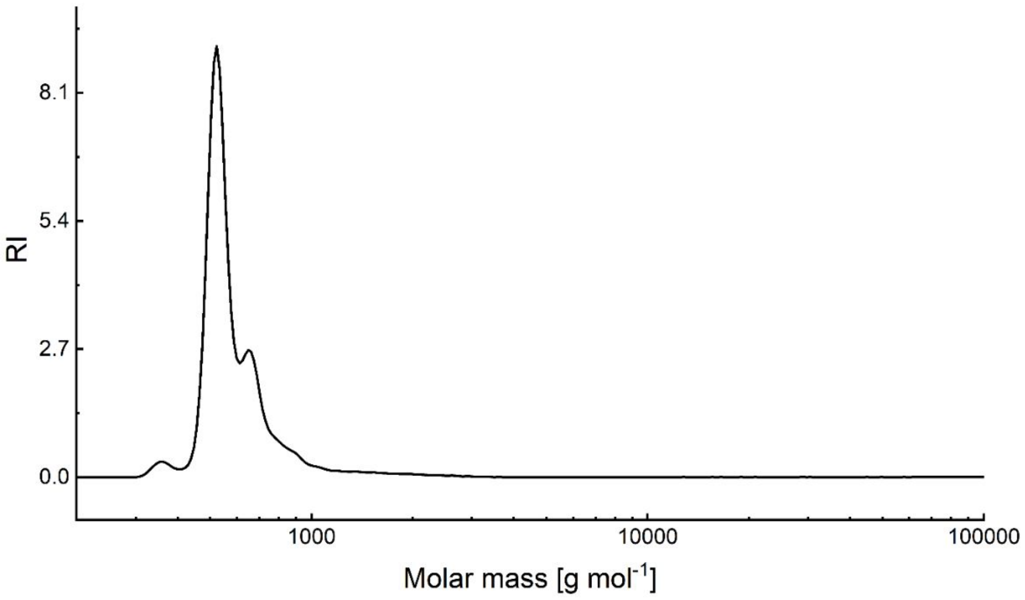

Figure S28. Molar mass determination of cyclic monomer (**M**) obtained from polymerization using bicarbonate in toluene, 66 h reaction time, by GPC calibrated with PS standard.

Chains:

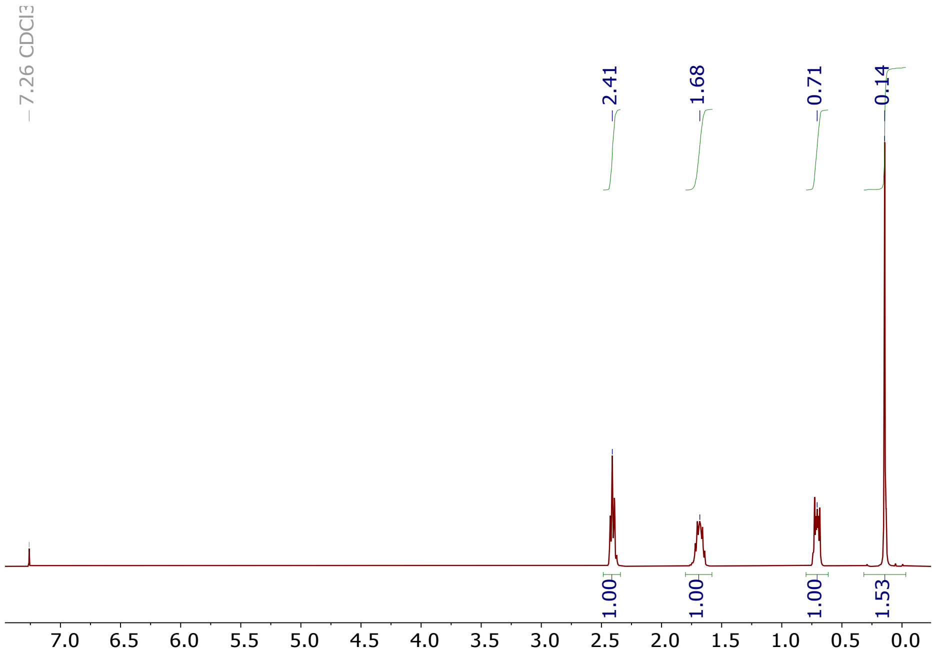

Figure S29. ^{1}H NMR spectrum of polymer ($\mathbf{P_{CN}}$**-OH**) obtained from polymerization using bicarbonate in toluene in $CDCl_3$.

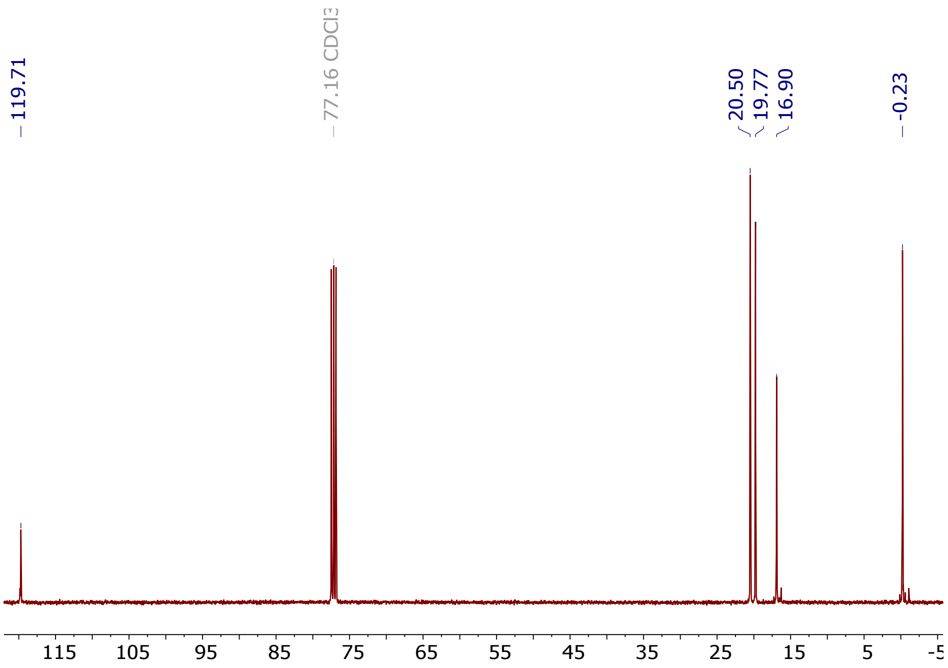

Figure S30. ^{13}C NMR spectrum of polymer (**P_{CN}-OH**) obtained from polymerization using bicarbonate in toluene in $CDCl_3$.

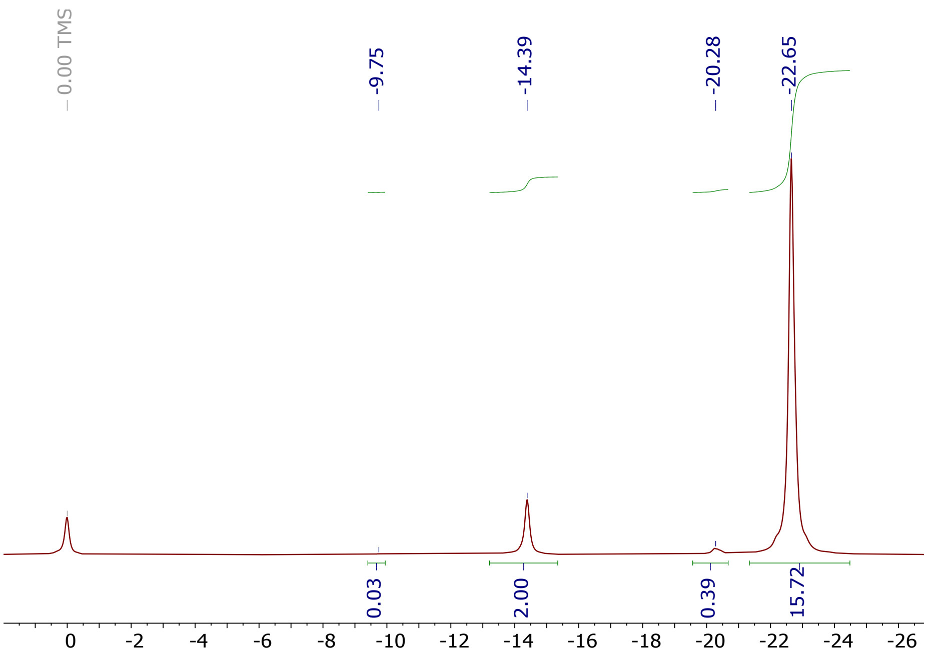

Figure S31. ^{29}Si NMR spectrum of polymer (**P_{CN}-OH**) obtained from polymerization using bicarbonate in toluene, 17 h reaction time. The spectrum was recorded using $Cr(acac)_3$ as a relaxation agent and TMS as a reference.

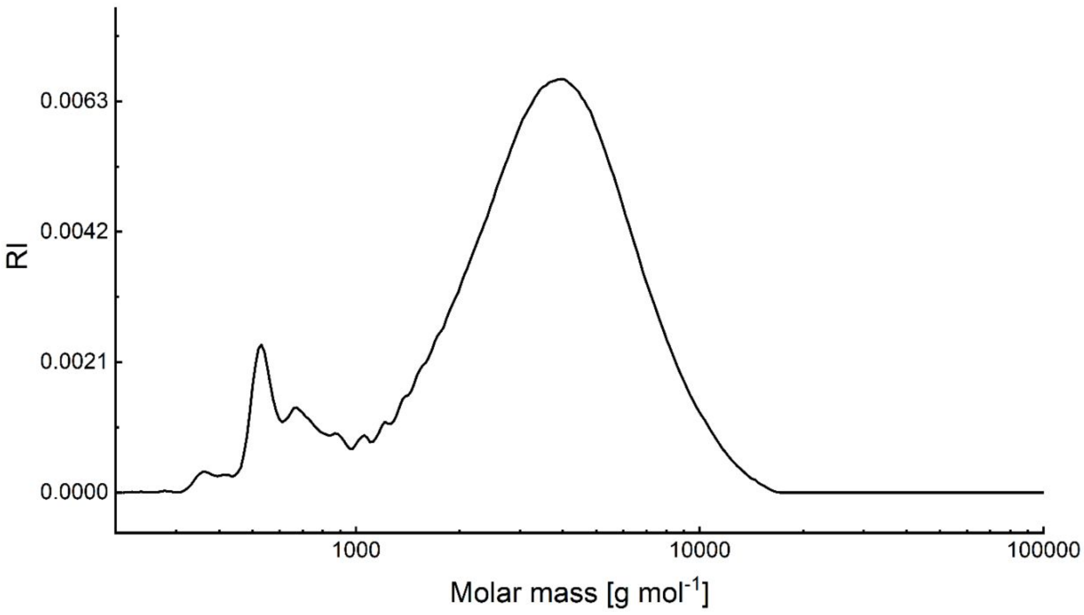

Figure S32. Molar mass determination of polymer (**P_{CN}-OH**) obtained from polymerization using bicarbonate in toluene, 17 h reaction time, by GPC calibrated with PS standard.

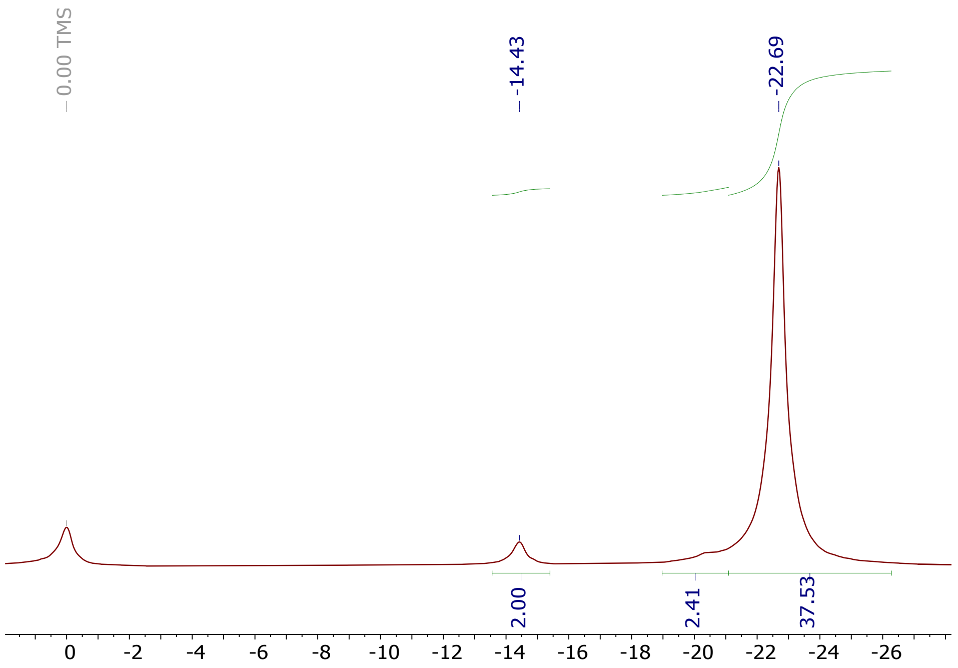

Figure S33. ^{29}Si NMR spectrum of polymer (**P_{CN}-OH**) obtained from polymerization using bicarbonate in toluene, 66 h reaction time. The spectrum was recorded using $Cr(acac)_3$ as a relaxation agent and TMS as a reference.

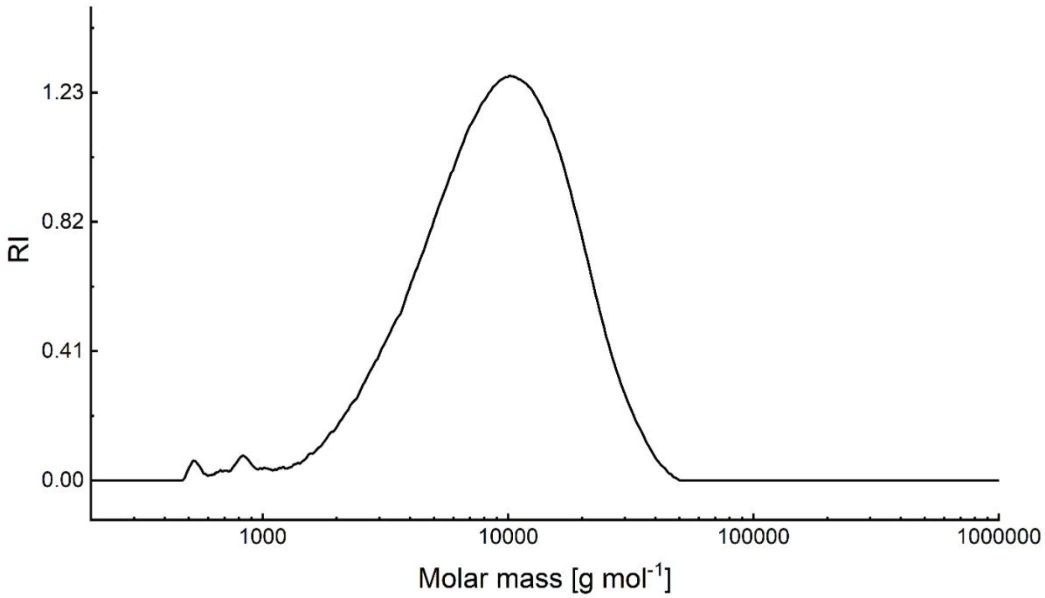

Figure S34. GPC chromatogram of polymer (**P_{CN}-OH**) obtained from polymerization using bicarbonate in toluene, 66 h reaction time. The GPC was calibrated with PS standard and used THF as a solvent.

Solvent free reaction using either bicarbonate or water

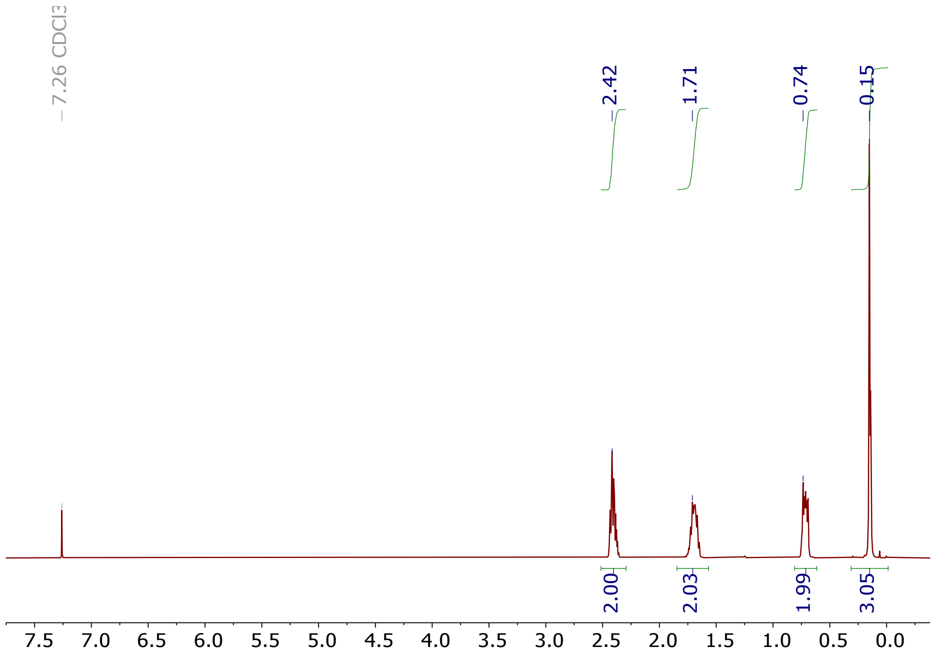

Figure S35. ^{1}H NMR spectrum in $CDCl_3$ of **P_{CN}-OH** obtained from solvent-free polymerization with 7 equivalents $NaHCO_3$.

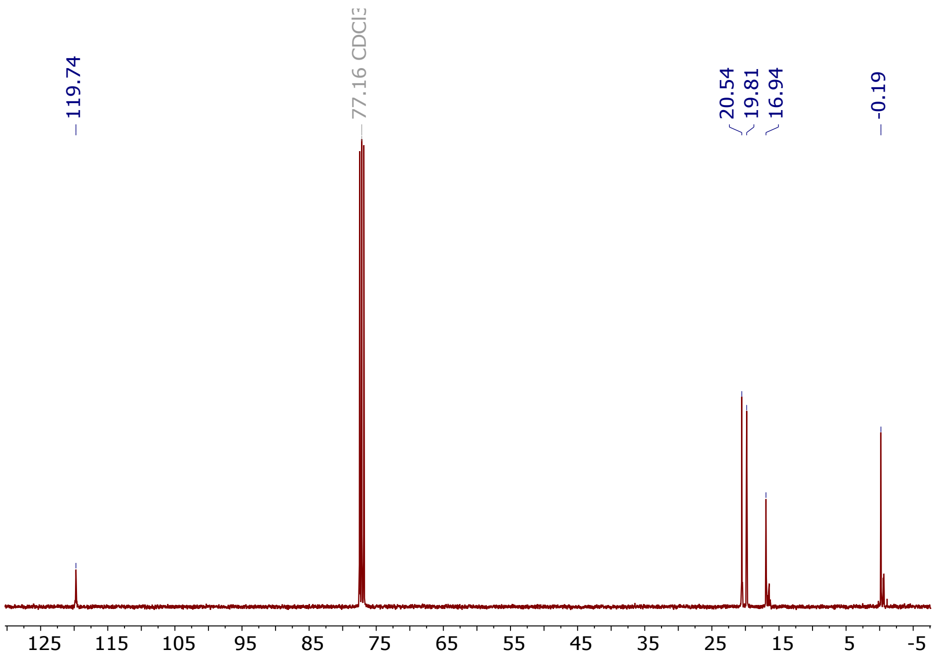

Figure S36. ^{13}C NMR spectrum in $CDCl_3$ of $\mathbf{P_{CN}}$**-OH** obtained from solvent-free polymerization in the presence of 7 equivalents $NaHCO_3$.

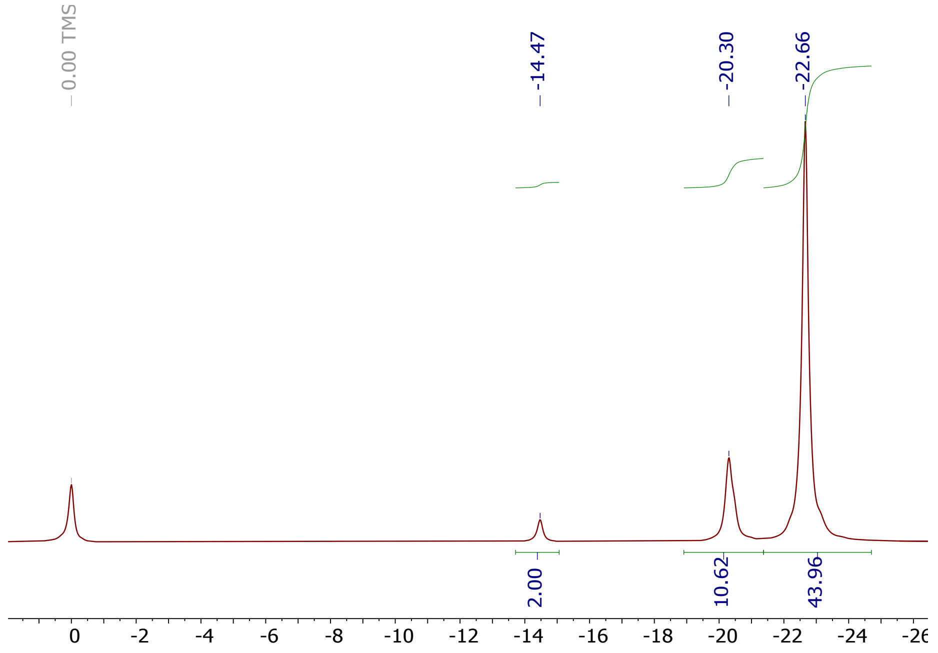

Figure S67. ^{29}Si NMR spectrum of $\mathbf{P_{CN}}$**-OH** obtained from solvent-free polymerization in the presence of 7 equivalents $NaHCO_3$. The spectrum was recorded using $Cr(acac)_3$ as a relaxation agent and TMS as a reference.

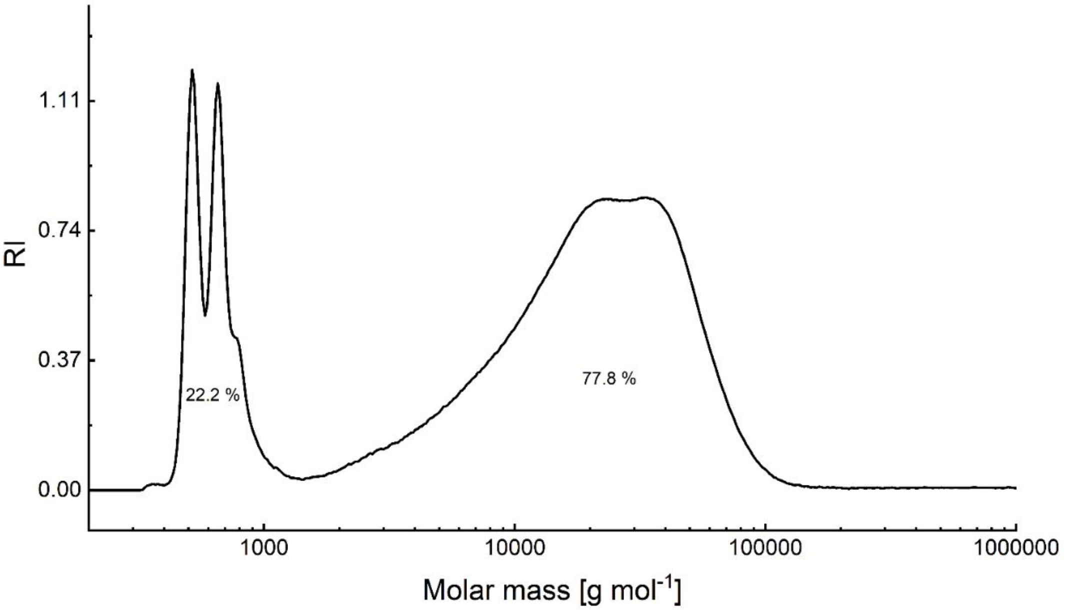

Figure S78. GPC chromatogram of **P_{CN}-OH** obtained from solvent-free polymerization in the presence of 7 equivalents $NaHCO_3$. The GPC was calibrated with PS standards and used THF as a solvent.

Solvent-free reaction using 3 equivalents $NaHCO_3$

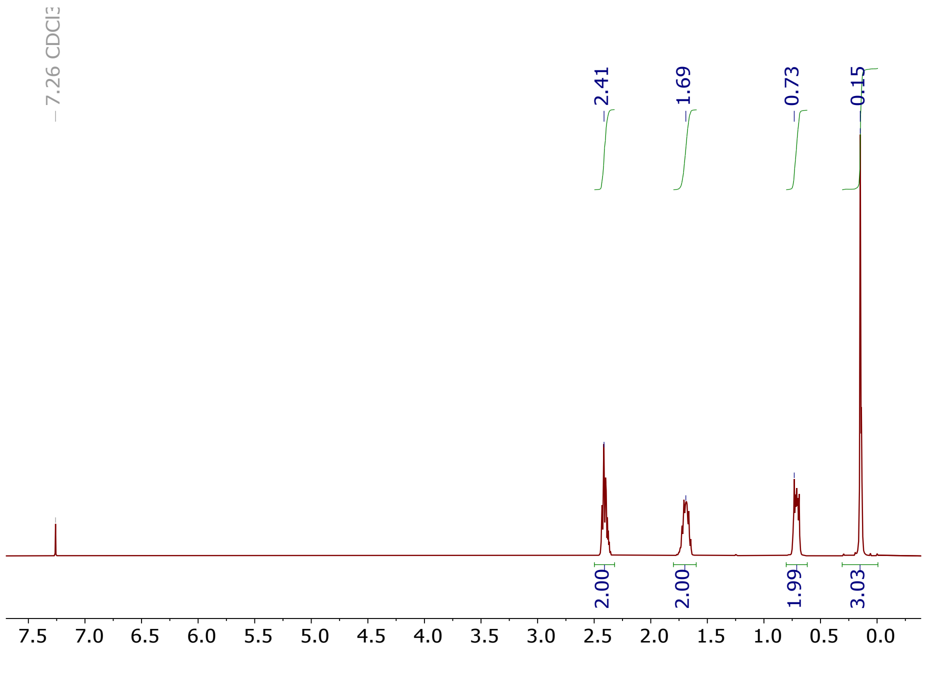

Figure S89. ^{1}H NMR spectrum in $CDCl_3$ of **P_{CN}-OH** obtained from solvent-free polymerization in the presence of 3 equivalents $NaHCO_3$.

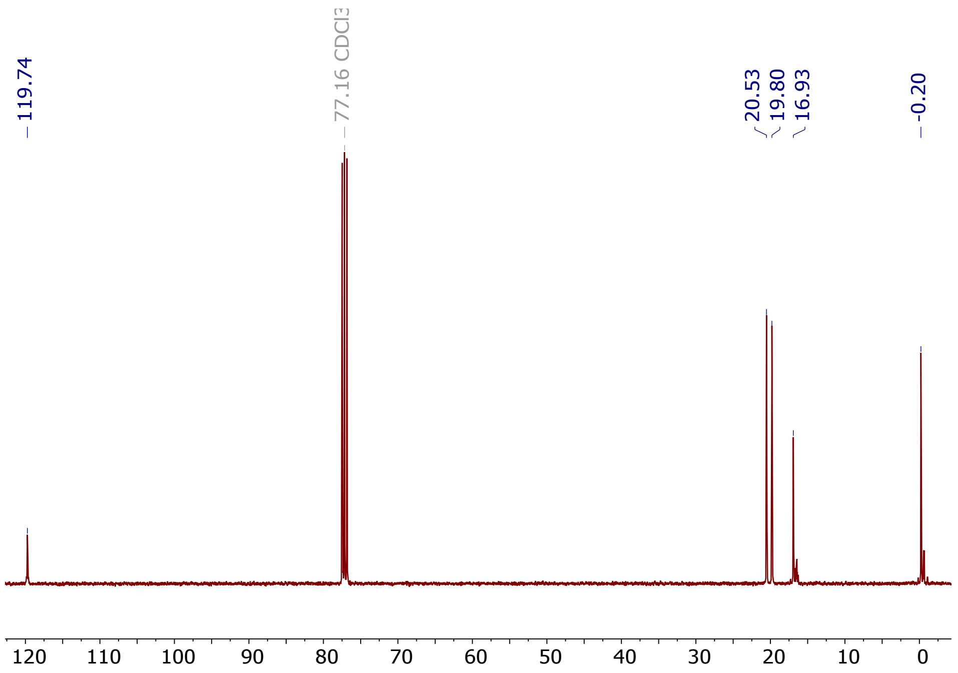

Figure S40. ^{13}C NMR spectrum in $CDCl_3$ of $\mathbf{P_{CN}}$**-OH** obtained from solvent-free polymerization in the presence of 3 equivalents $NaHCO_3$.

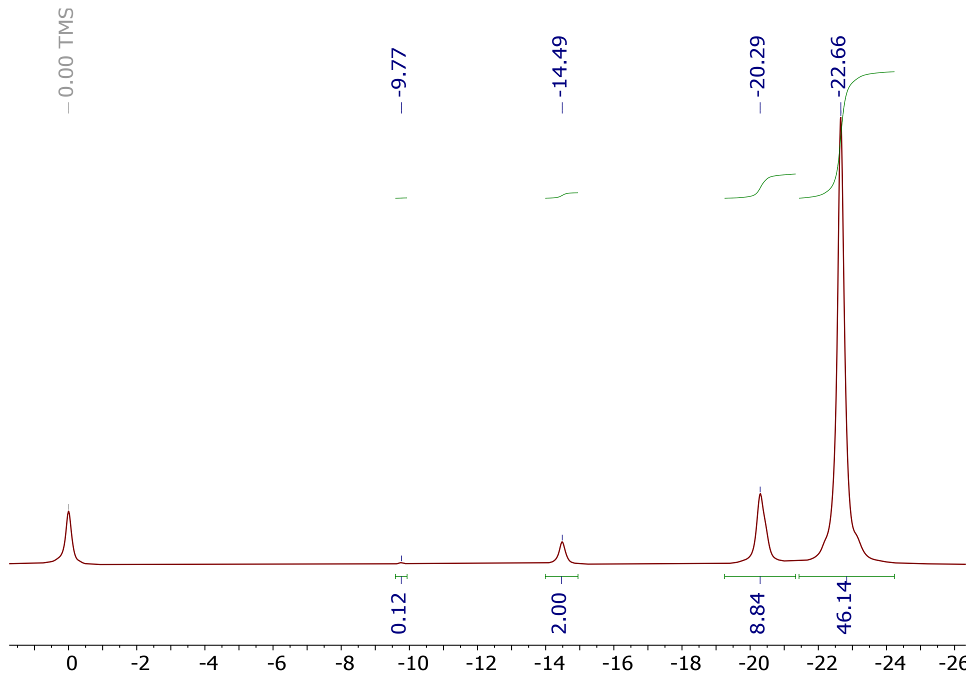

Figure S41. ^{29}Si NMR spectrum of **P$_{CN}$-OH** obtained from solvent-free polymerization in the presence of 3 equivalents $NaHCO_3$. The spectrum was recorded using $Cr(acac)_3$ as a relaxation agent and TMS as a reference.

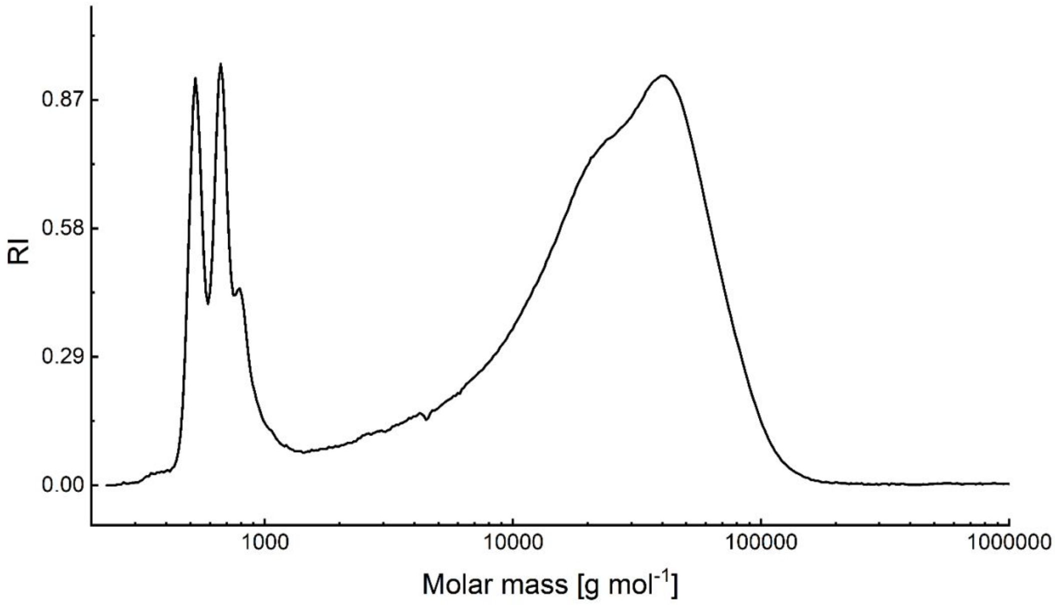

Figure S42. GPC chromatogram of **P$_{CN}$-OH** obtained from solvent-free polymerization in the presence of 3 equivalents $NaHCO_3$. The GPC was calibrated with PS standards using THF as a solvent.

Reaction conducted with 2 equivalents of H_2O:

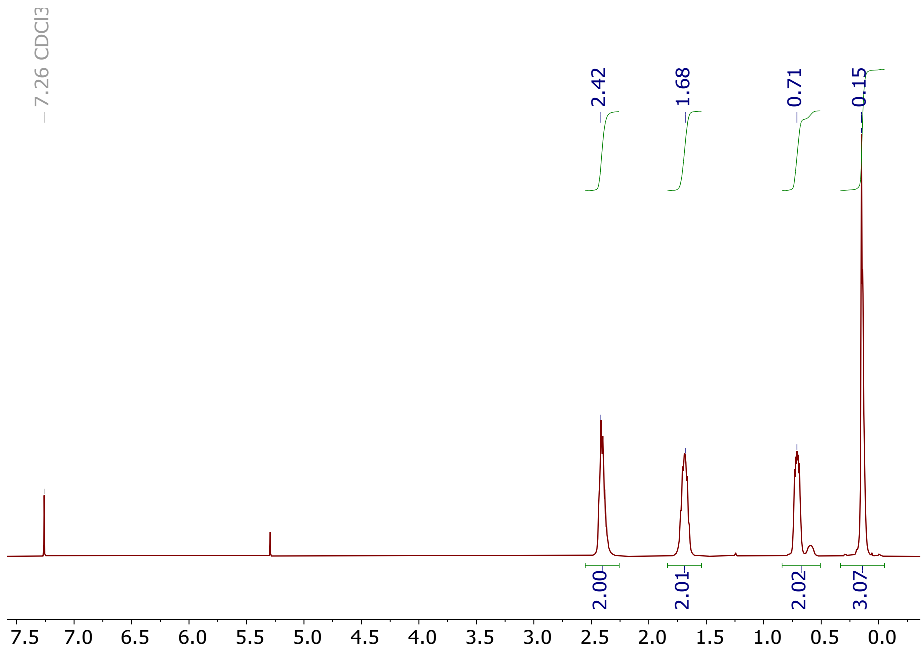

Figure S43. ^{1}H NMR spectrum in $CDCl_3$ of **P_{CN}-OH** obtained from solvent-free polymerization in the presence of 2 equivalents H_2O.

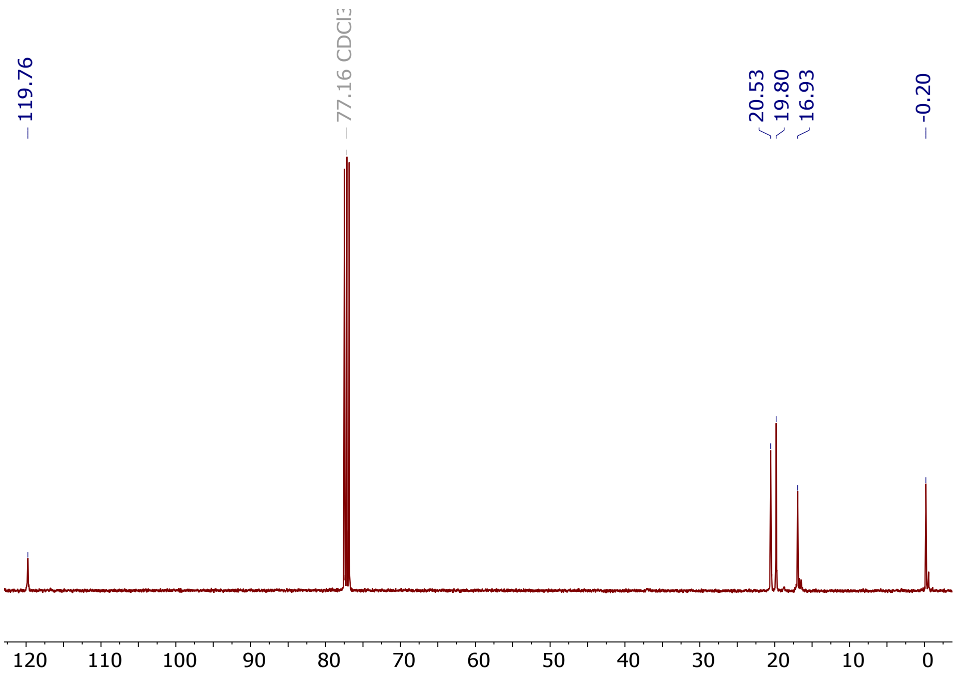

Figure S44. ^{13}C NMR spectrum in $CDCl_3$ of **P_{CN}-OH** obtained from solvent-free polymerization in the presence of 2 equivalents H_2O.

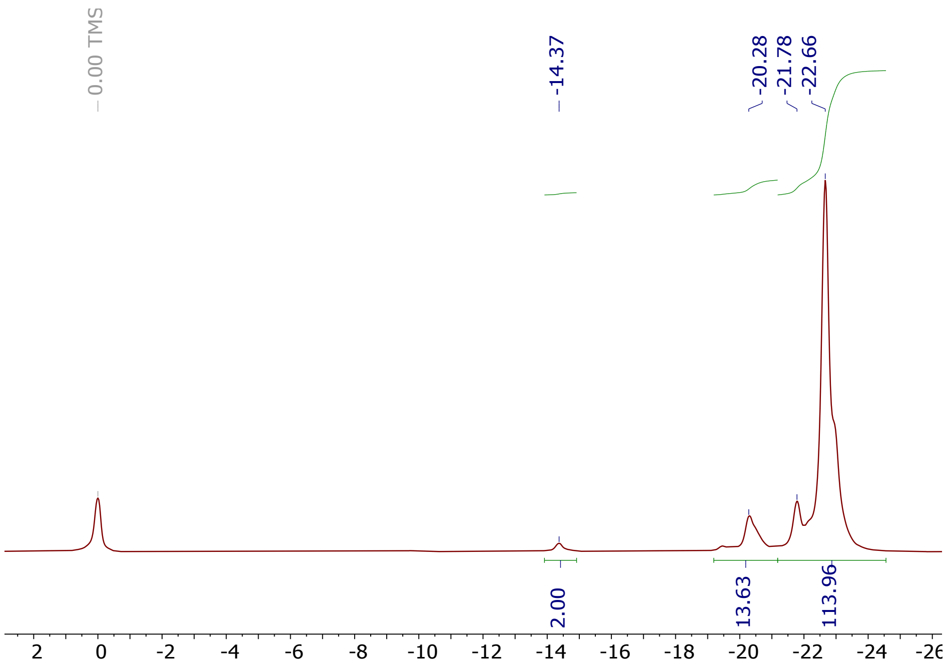

Figure S45. ^{29}Si NMR spectrum of $\mathbf{P_{CN}}$**-OH** obtained from solvent-free polymerization in the presence of 2 equivalents H_2O. The spectrum was recorded using $Cr(acac)_3$ as a relaxation agent and TMS as a reference.

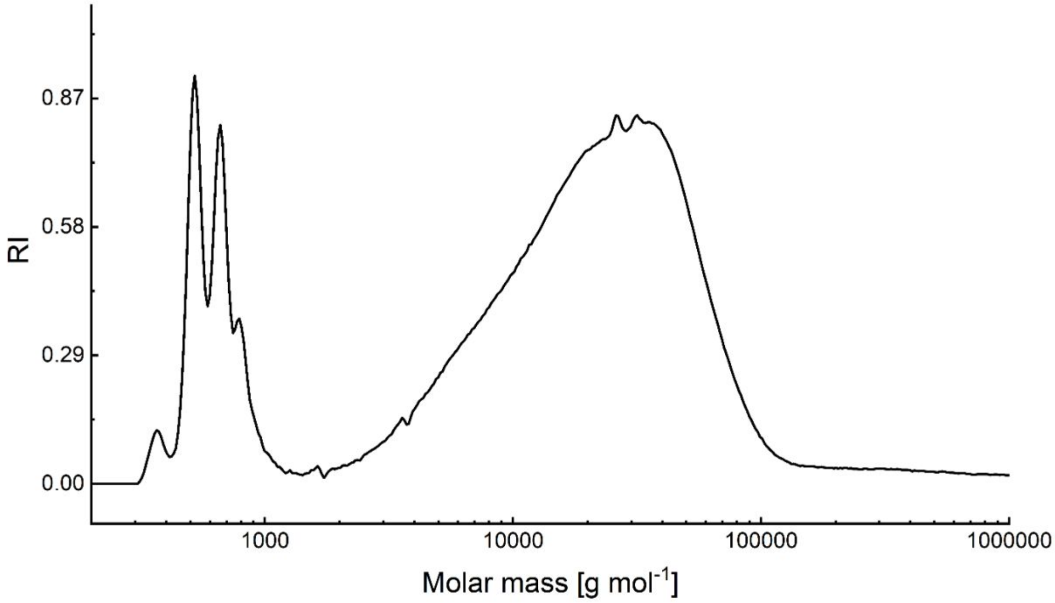

Figure S96. GPC chromatogram of $\mathbf{P_{CN}}$**-OH** obtained from solvent-free polymerization in the presence of 2 equivalents H_2O. The GPC was done in THF and was calibrated with PS standards.

Reaction conducted with 1 equivalent H_2O:

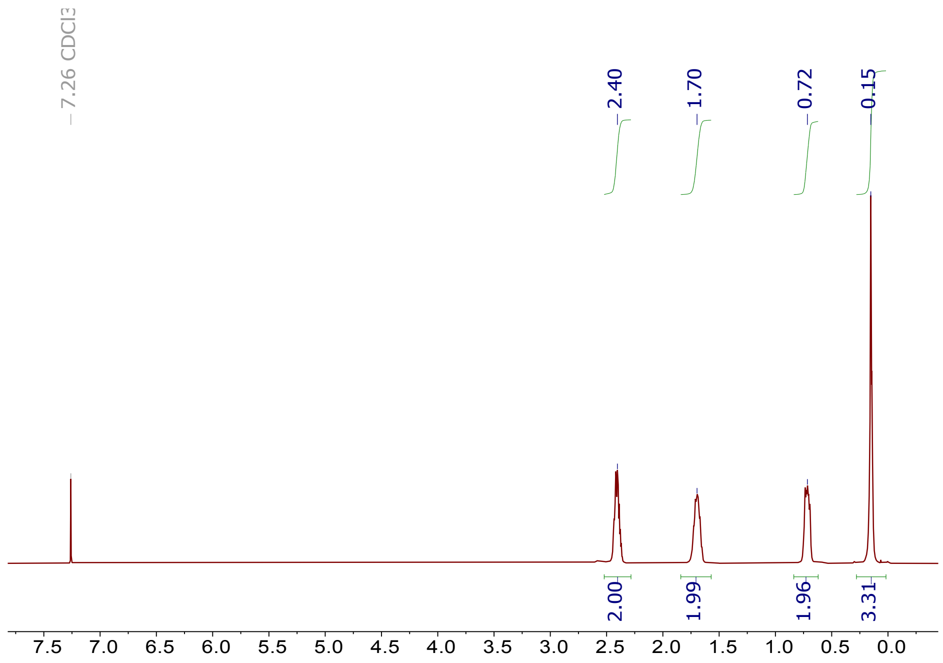

Figure S107. ^{1}H NMR spectrum of **P_{CN}-OH** obtained from solvent-free polymerization in the presence of 1 equivalents H_2O in $CDCl_3$.

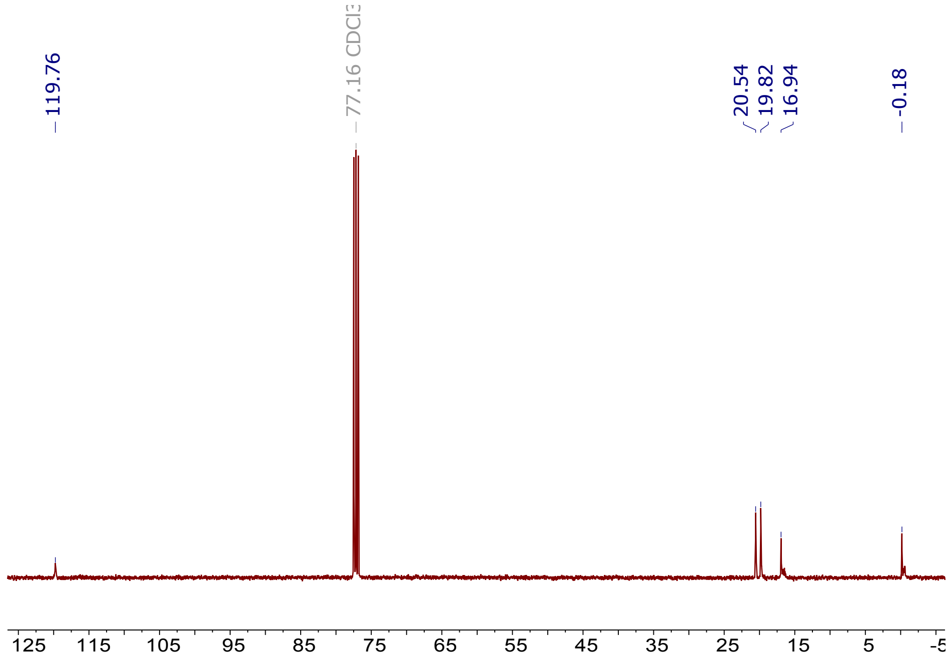

Figure S118. ^{13}C NMR spectrum of **P_{CN}-OH** obtained from solvent-free polymerization in the presence of 1 equivalents H_2O in $CDCl_3$.

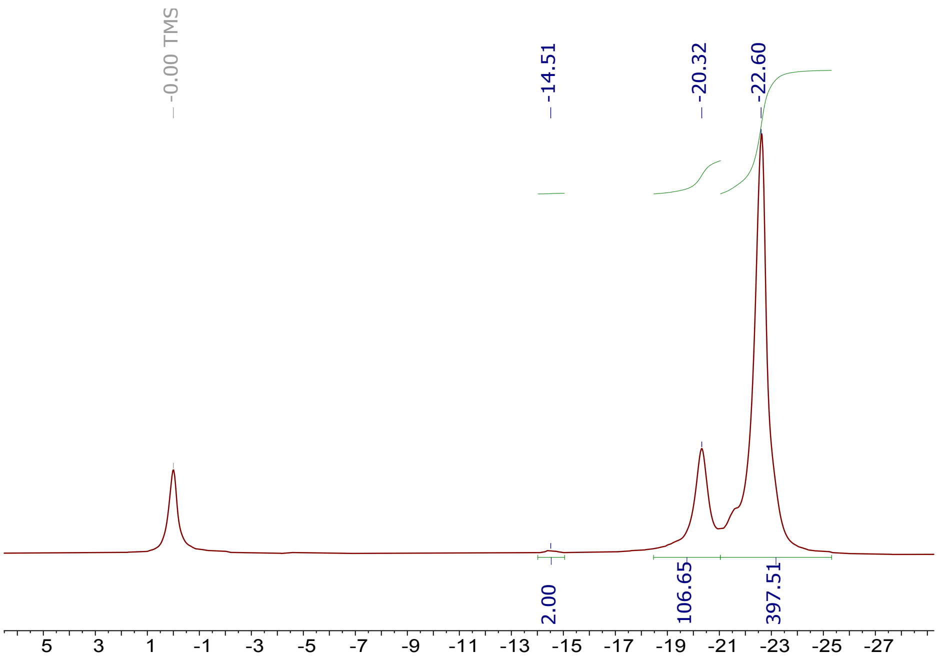

Figure S49. ^{29}Si NMR spectrum of **P$_{CN}$-OH** obtained from solvent-free polymerization in the presence of 1 equivalents H_2O. The spectrum was recorded using Cr(acac)$_3$ as a relaxation agent and TMS as a reference.

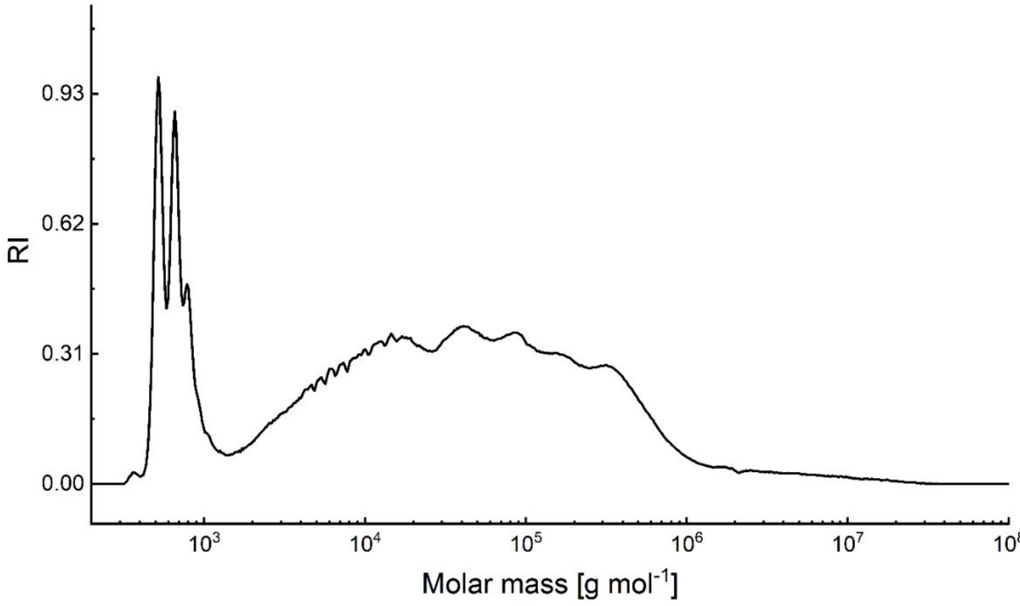

Figure S50. GPC chromatogram of **P$_{CN}$-OH** obtained from solvent-free polymerization in the presence of 1 equivalents H_2O. The GPC was done in THF and was calibrated with PS standards.

Separation of cycles and chains

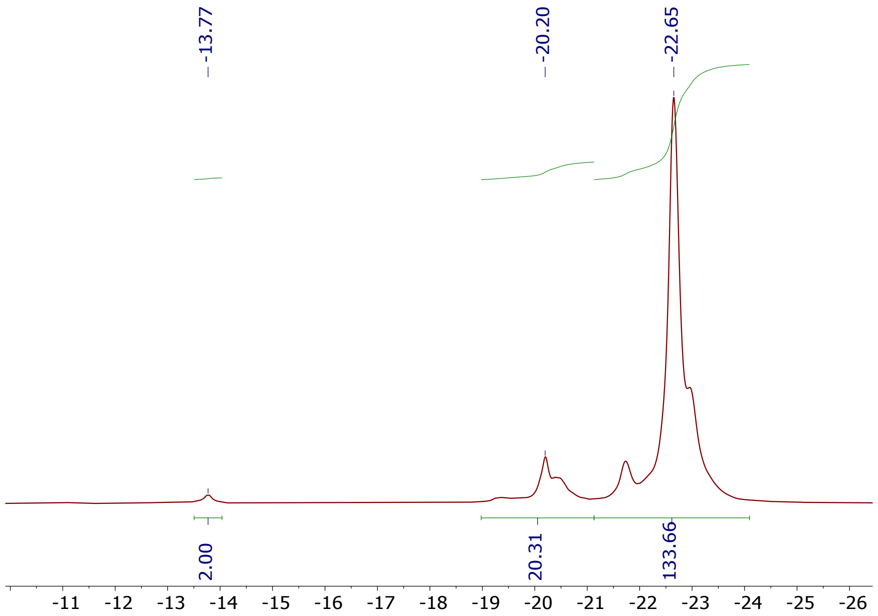

Figure S51. ^{29}Si NMR spectrum of unpurified **P_{CN}-OH** showing a mixture of cycles and chains with $Cr(acac)_3$ as relaxation agent and TMS as reference.

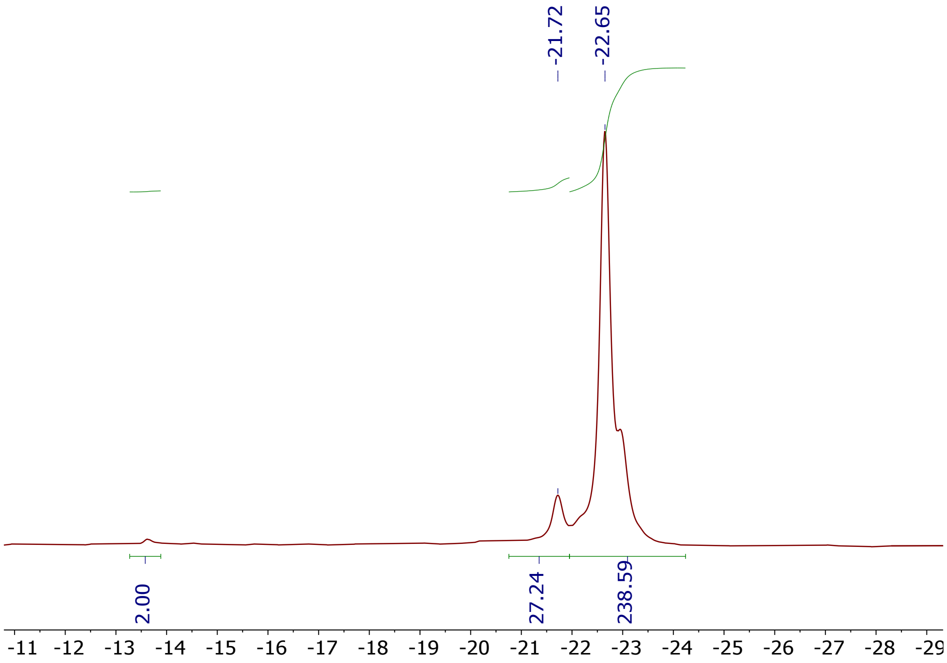

Figure S52. ^{29}Si NMR spectrum of purified **P_{CN}-OH** showing less cycles than chains. The spectrum was recorded using $Cr(acac)_3$ as a relaxation agent and TMS as a reference.

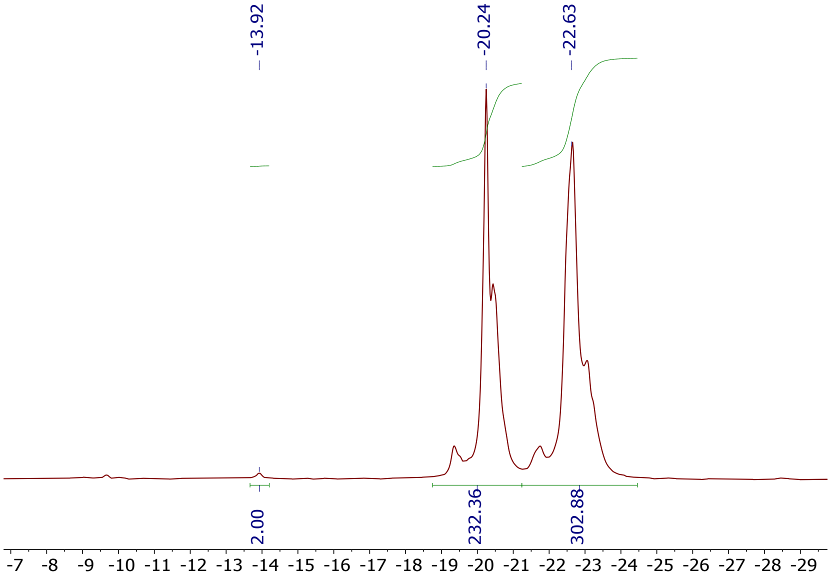

Figure S53. ^{29}Si NMR spectrum of toluene phase of purified $\mathbf{P_{CN}}$**-OH** showing more cycles than chains with $Cr(acac)_3$ as relaxation agent and TMS as reference.

AROP of cycles

LiOH (40 °C)

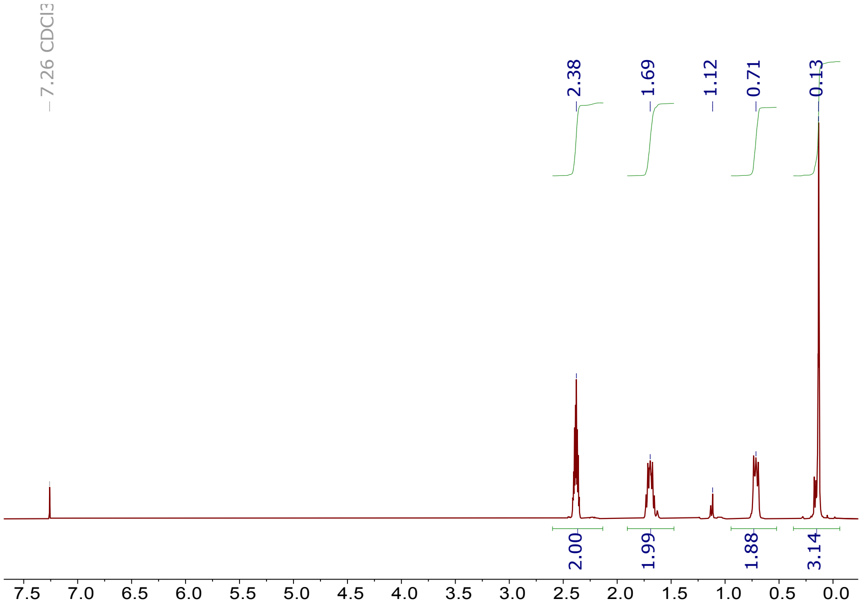

Figure S54. ^{1}H NMR spectrum in $CDCl_3$ of $\mathbf{P_{CN}}$**-OH** obtained from solvent-free AROP at 40 °C with 0.1 equivalents LiOH as initiator.

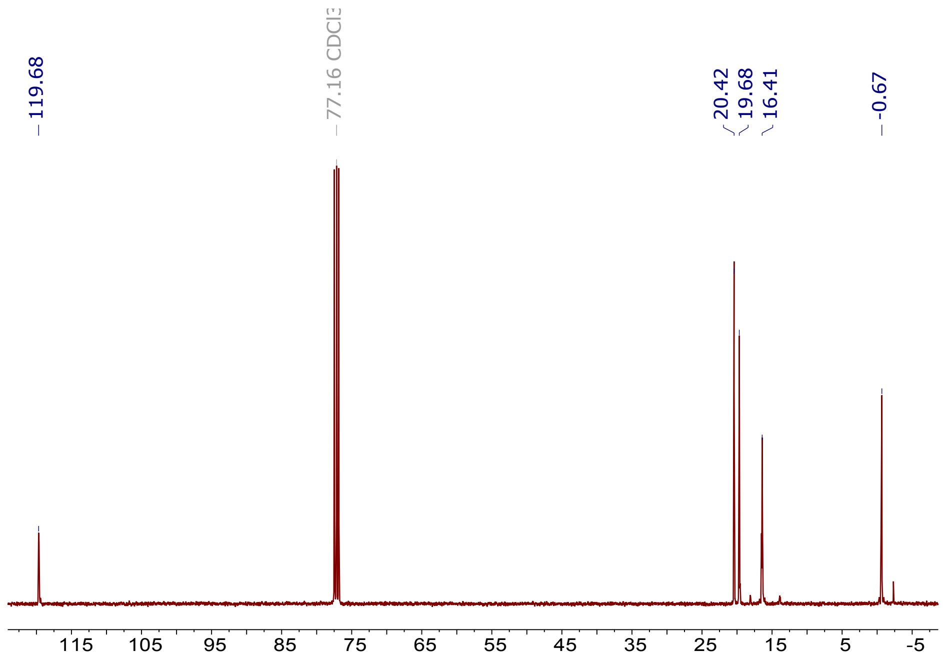

Figure S55. ^{13}C NMR spectrum in $CDCl_3$ of $\mathbf{P_{CN}}$**-OH** obtained from solvent-free AROP at 40 °C with 0.1 equivalents LiOH as initiator.

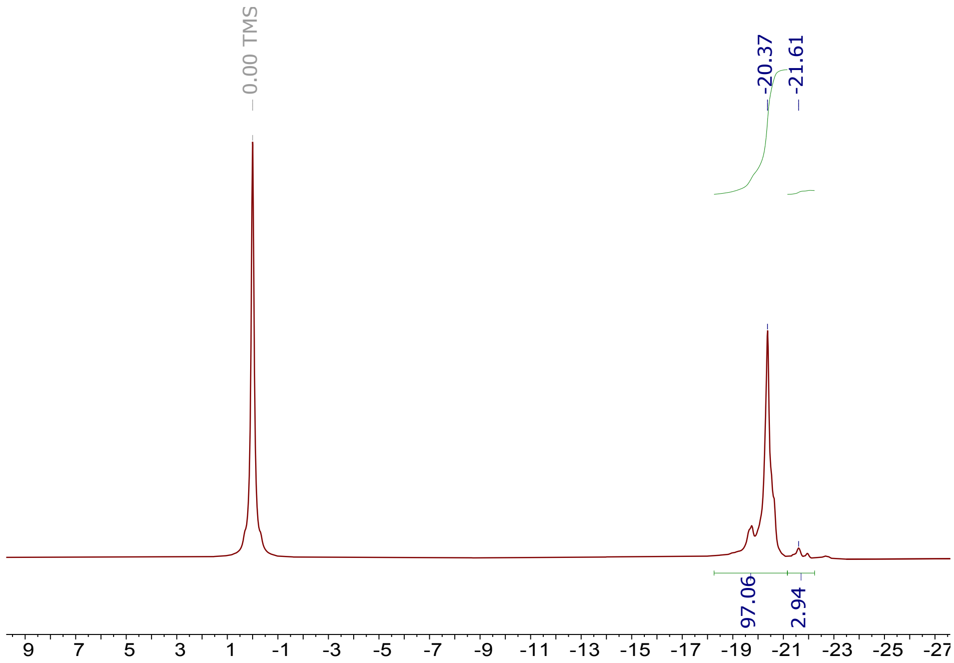

Figure S56. ^{29}Si NMR spectrum of $\mathbf{P_{CN}}$**-OH** obtained from solvent-free AROP at 40 °Cwith 0.1 equivalents LiOH as initiator. The spectrum was recorded using $Cr(acac)_3$ as a relaxation agent and TMS as a reference.

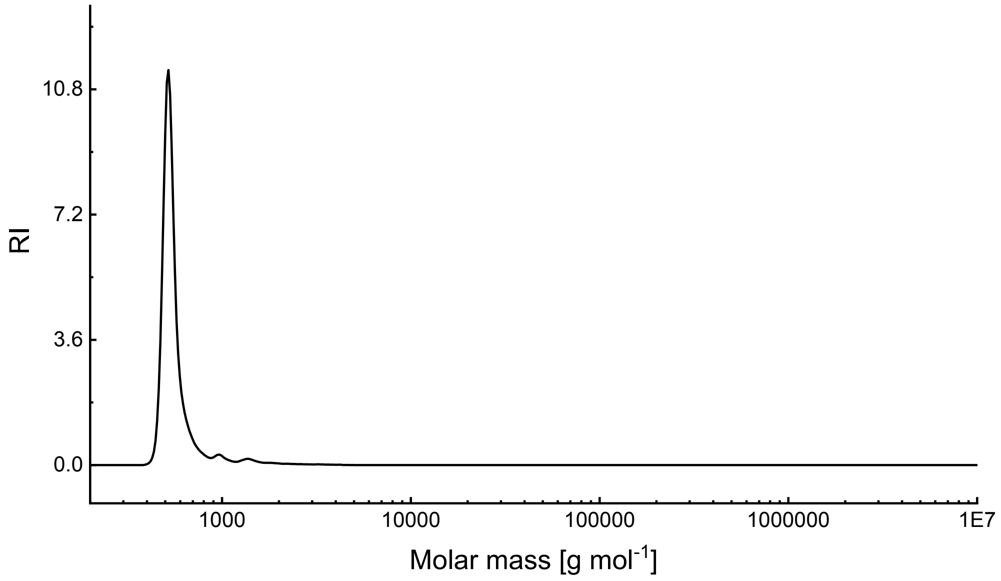

Figure S57. GPC chromatogram of **P_{CN}-OH** obtained from solvent-free AROP at 40 °C with 0.1 equivalents LiOH as initiator. The GPC was done in THF and was calibrated with PS standards.

NaOH (40 °C)

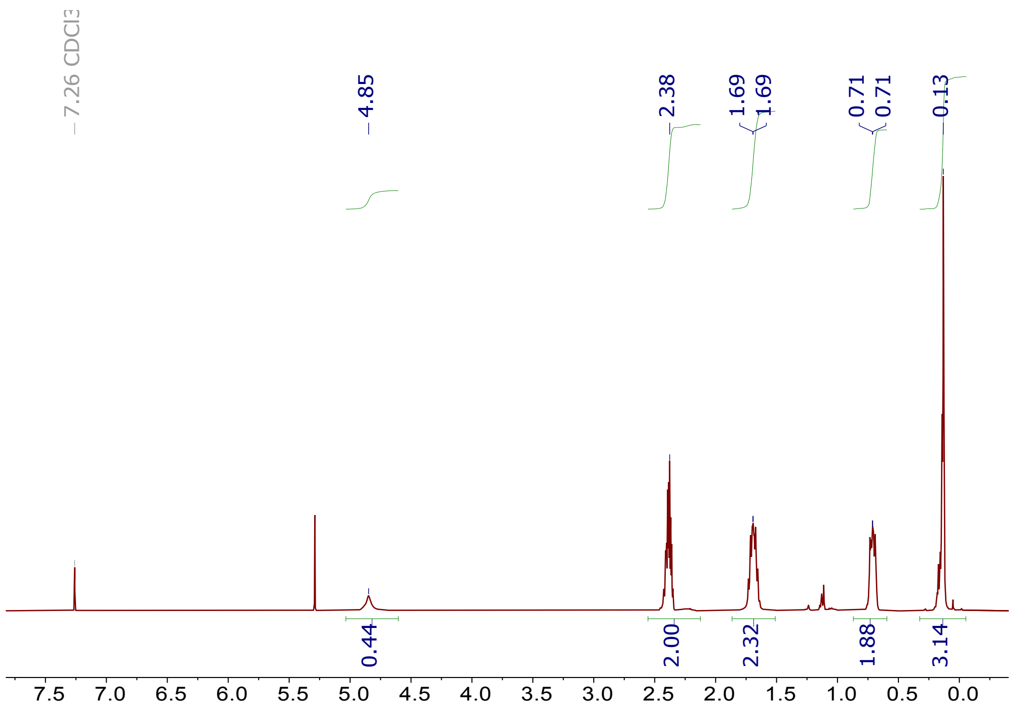

Figure S58. ^{1}H NMR spectrum in $CDCl_3$ of **P_{CN}-OH** obtained from solvent-free AROP at 40 °C with 0.1 equivalents NaOH as initiator.

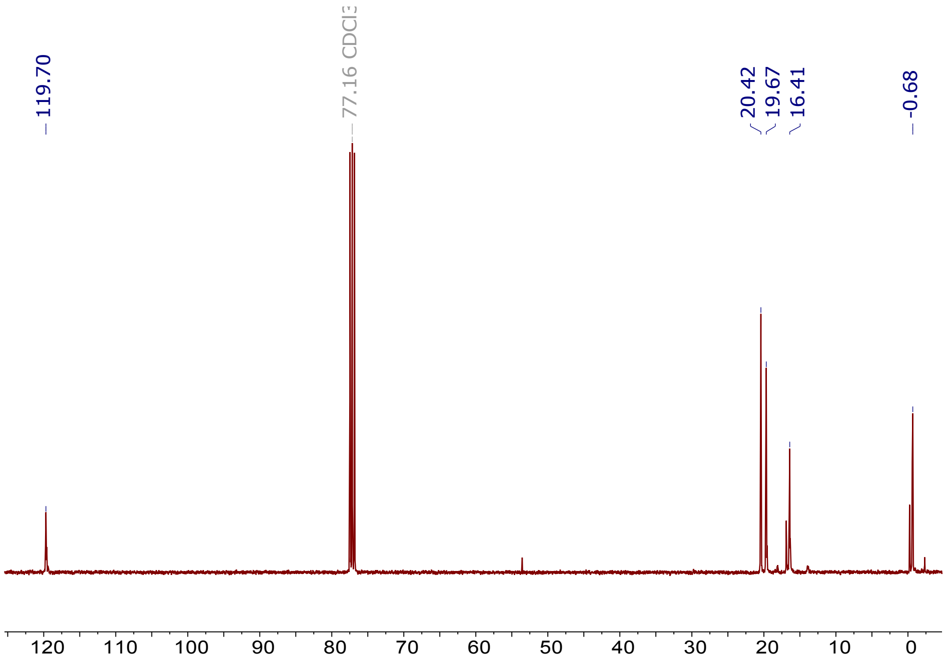

Figure S59. ^{13}C NMR spectrum in $CDCl_3$ of **P_{CN}-OH** obtained from solvent-free AROP at 40 °C with 0.1 equivalents NaOH as initiator.

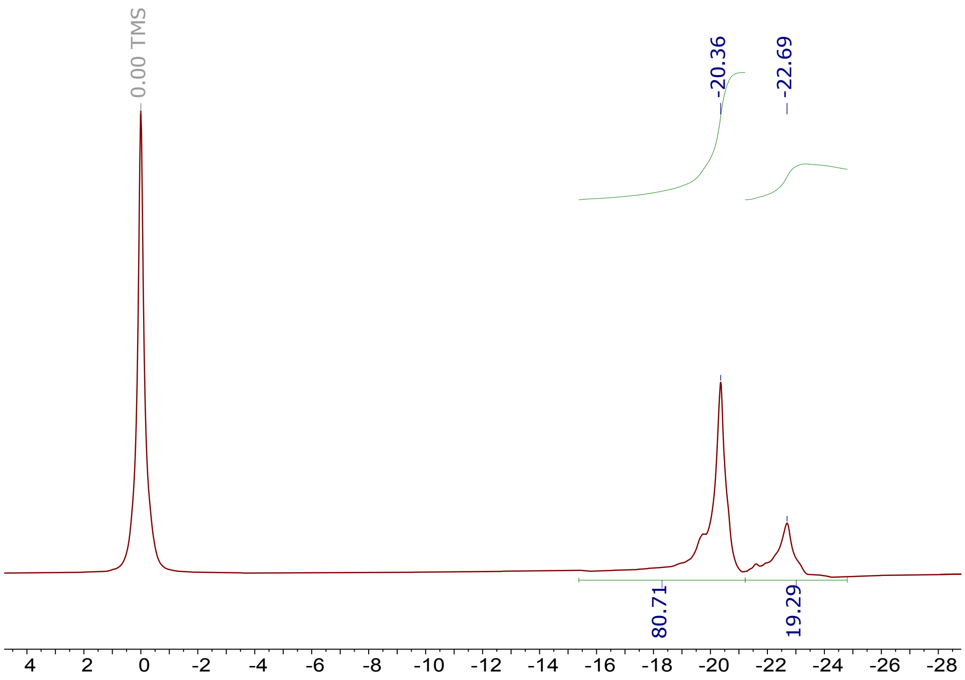

Figure S60. ^{29}Si NMR spectrum of **P_{CN}-OH** obtained from solvent-free AROP at 40 °C with 0.1 equivalents NaOH as initiator. The spectrum was recorded using $Cr(acac)_3$ as a relaxation agent and TMS as a reference.

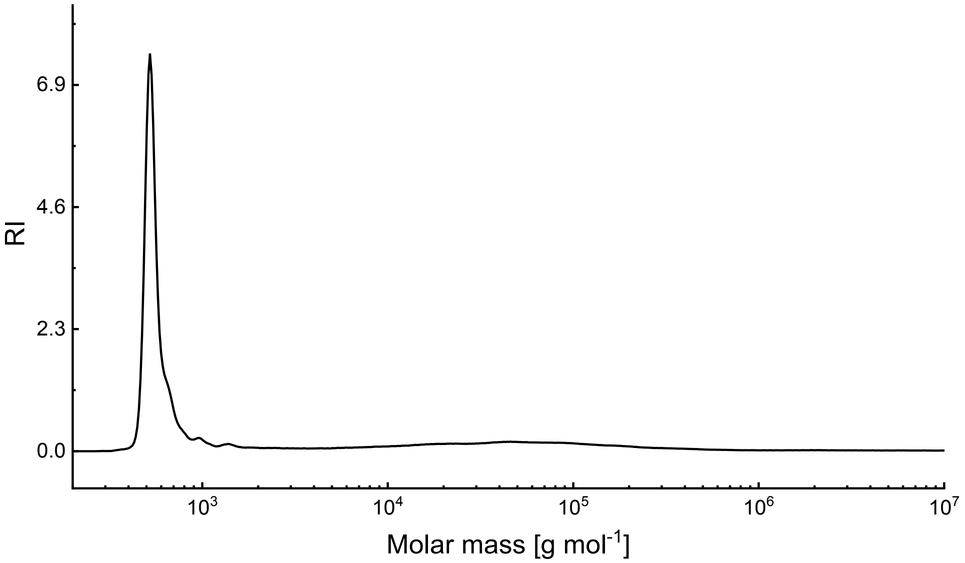

Figure S61. GPC chromatogram of **P_{CN}-OH** obtained from solvent-free AROP at 40 °C with 0.1 equivalents NaOH as initiator. The GPC was done in THF and was calibrated with PS standards.

NaOH (60 ˚C)

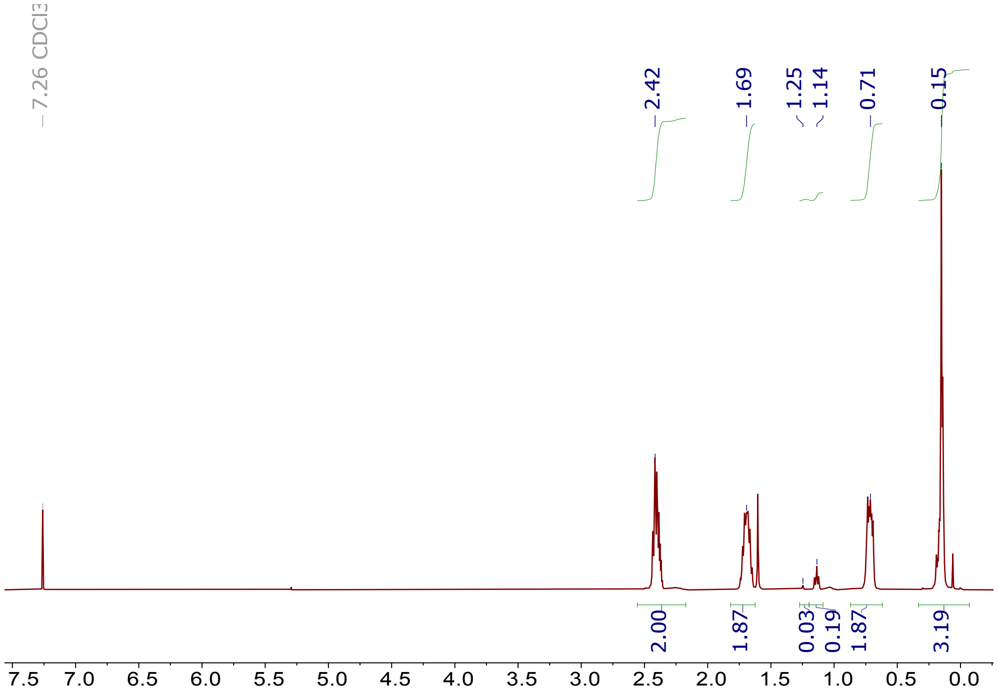

Figure S62. ^{1}H NMR spectrum in $CDCl_3$ of **P_{CN}-OH** obtained from solvent-free AROP at 60 °C with 0.1 equivalents NaOH as initiator.

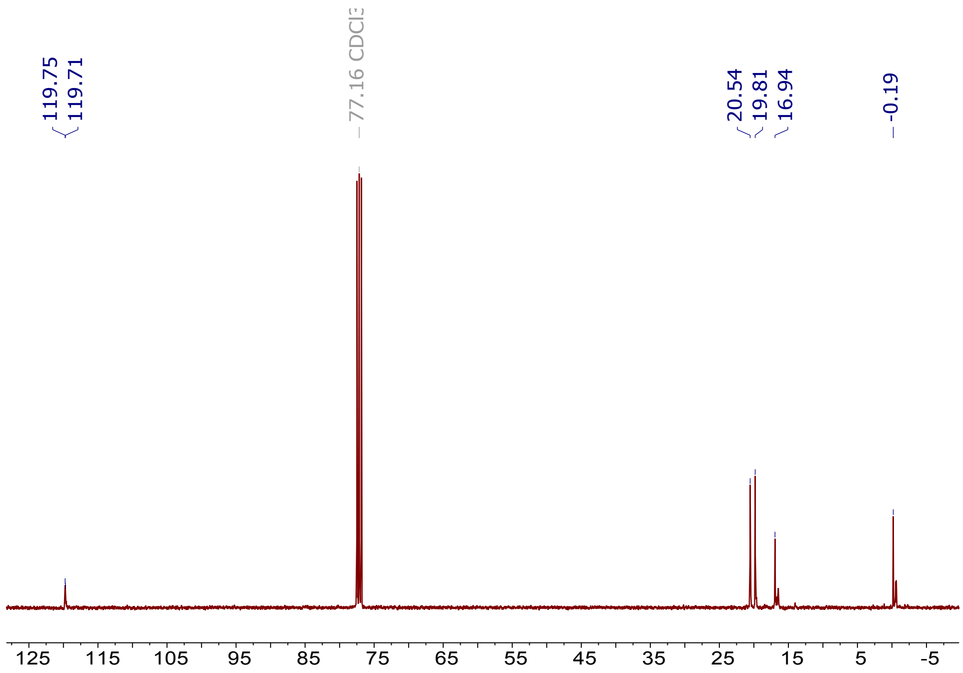

Figure S63. ^{13}C NMR spectrum in $CDCl_3$ of **P_{CN}-OH** obtained from solvent-free AROP at 60 °C with 0.1 equivalents NaOH as initiator.

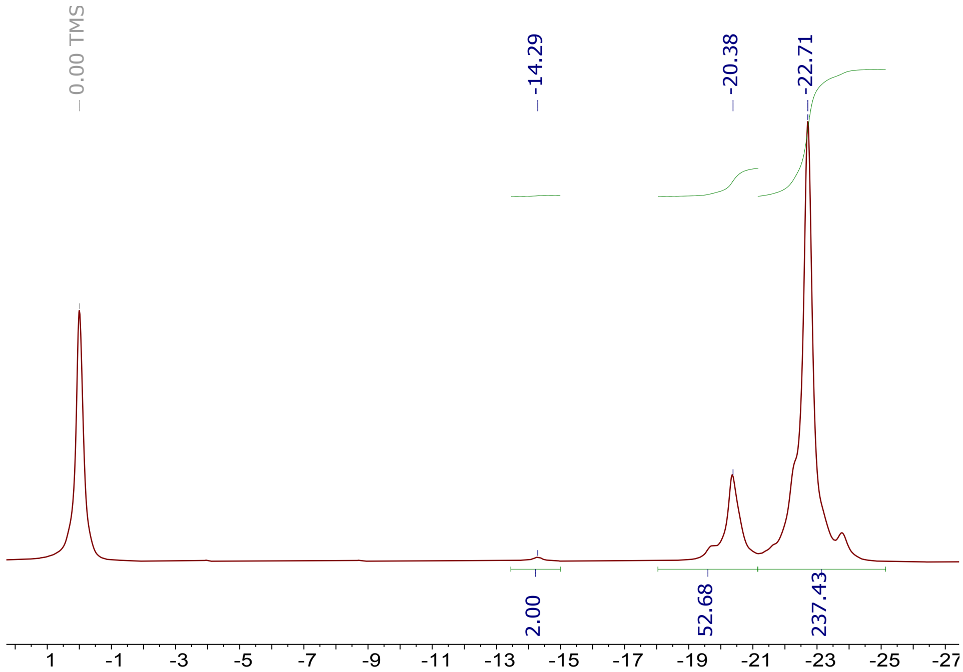

Figure S64. ^{29}Si NMR spectrum of **P_{CN}-OH** obtained from solvent-free AROP at 60 °C with 0.1 equivalents NaOH as initiator. The spectrum was recorded using $Cr(acac)_3$ as a relaxation agent and TMS as a reference.

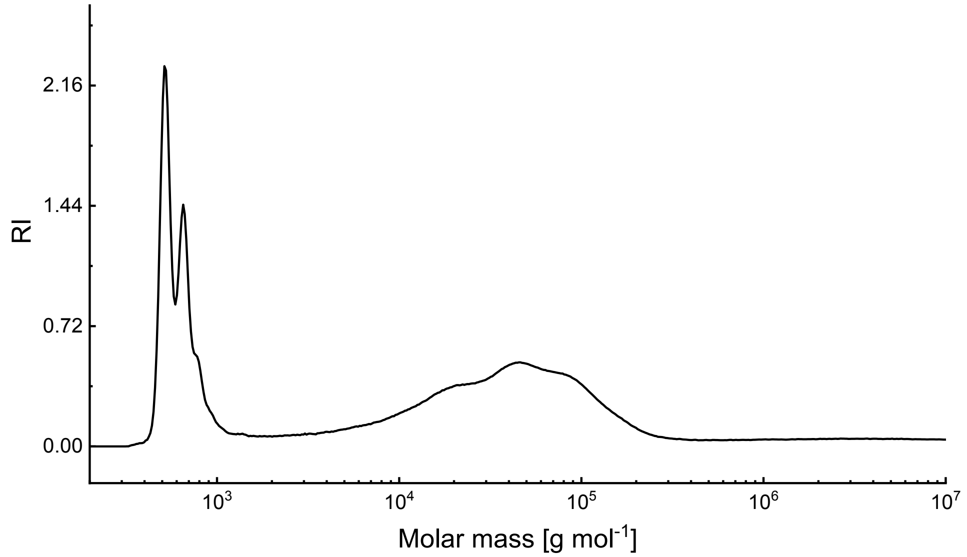

Figure S65. GPC chromatogram of **P_{CN}-OH** obtained from solvent-free AROP at 60 °C with 0.1 equivalents NaOH as initiator. The GPC was done in THF and was calibrated with PS standards.

NaOH (80 °C)

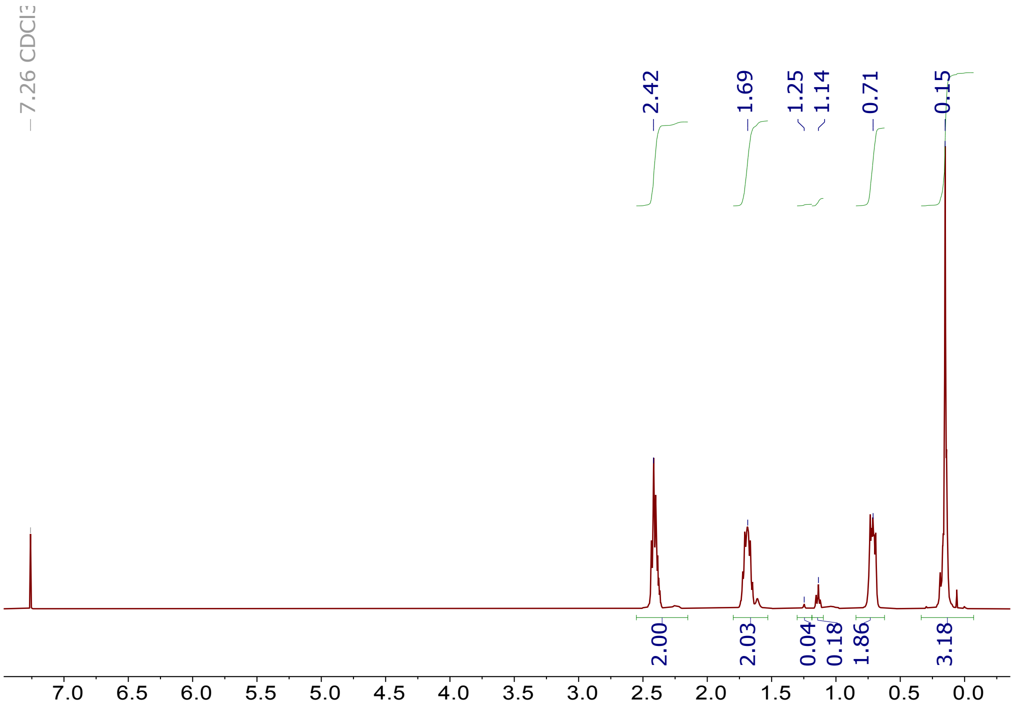

Figure S66. ^{1}H NMR spectrum in $CDCl_3$ of **P_{CN}-OH** obtained from solvent-free AROP at 80 °C with 0.1 equivalents NaOH as initiator.

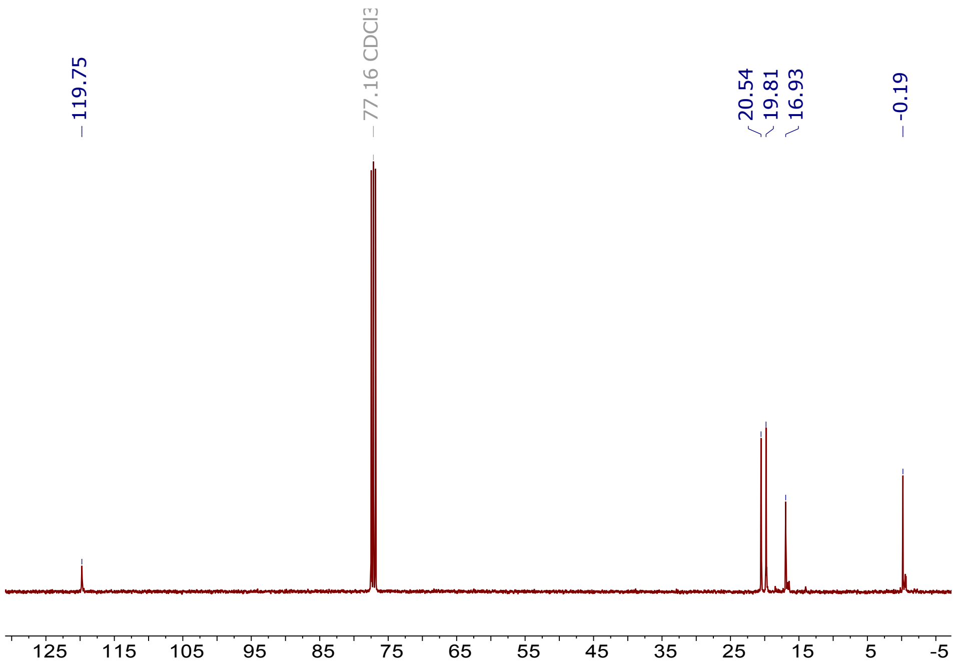

Figure S67. ^{13}C NMR spectrum in $CDCl_3$ of $\mathbf{P_{CN}}$**-OH** obtained from solvent-free AROP at 80 °C with 0.1 equivalents NaOH as initiator.

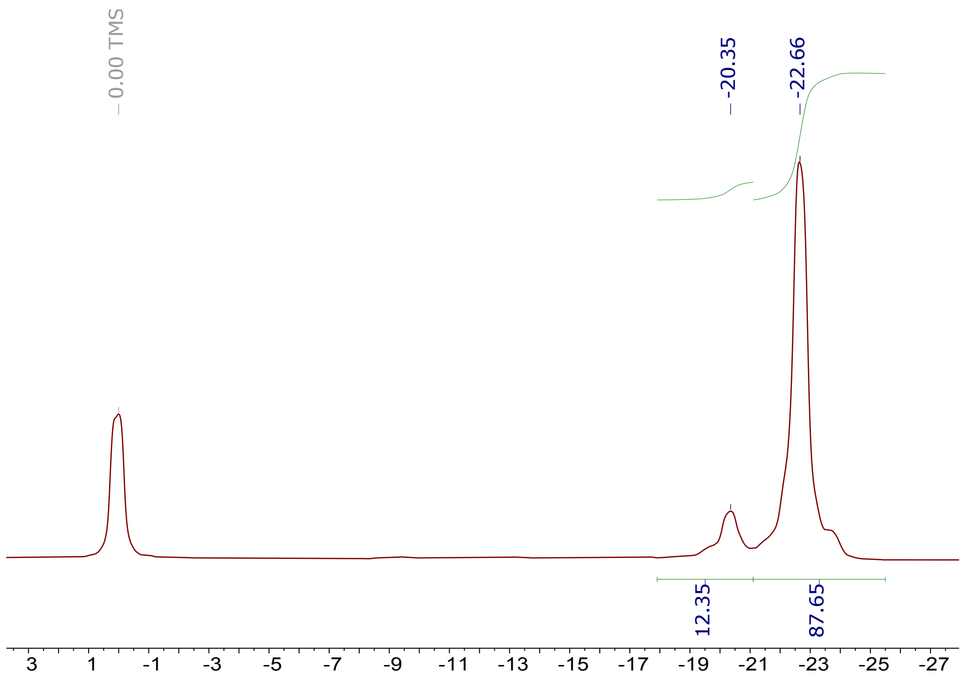

Figure S68. ^{29}Si NMR spectrum of $\mathbf{P_{CN}}$**-OH** obtained from solvent-free AROP at 80 °C with 0.1 equivalents NaOH as initiator. The spectrum was recorded using $Cr(acac)_3$ as a relaxation agent and TMS as a reference.

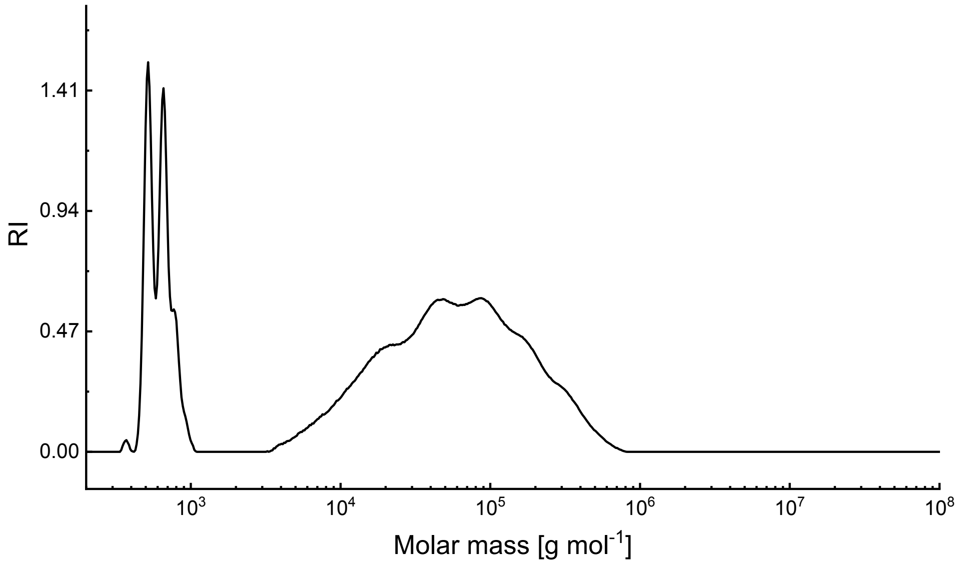

Figure S69. GPC chromatogram of **P_{CN}-OH** obtained from solvent-free AROP at 80 °C with 0.1 equivalents NaOH as initiator. The GPC was done in THF and was calibrated with PS standards.

NaOH (100 °C)

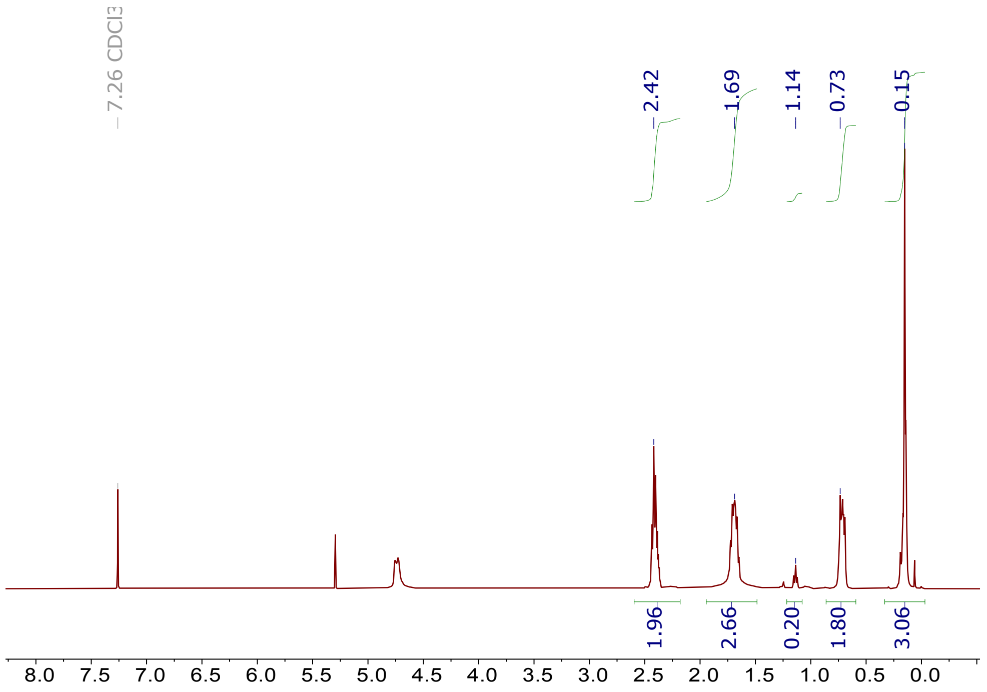

Figure S70. ^{1}H NMR spectrum in $CDCl_3$ of **P_{CN}-OH** obtained from solvent-free AROP at 100 °C with 0.1 equivalents NaOH as initiator.

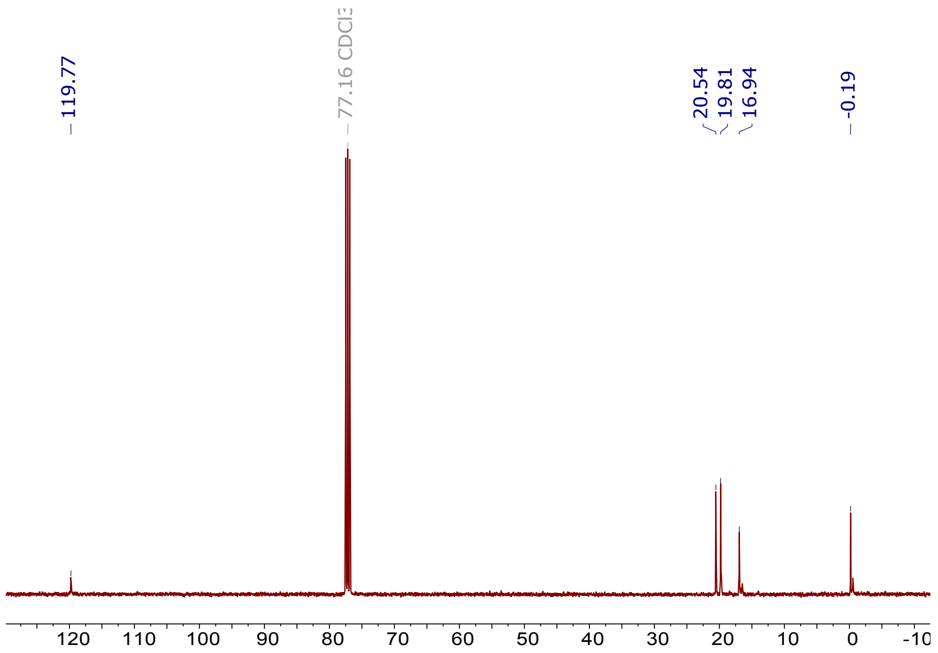

Figure S71. ^{13}C NMR spectrum in $CDCl_3$ of $\mathbf{P_{CN}}$**-OH** obtained from solvent-free AROP at 100 °C with 0.1 equivalents NaOH as initiator.

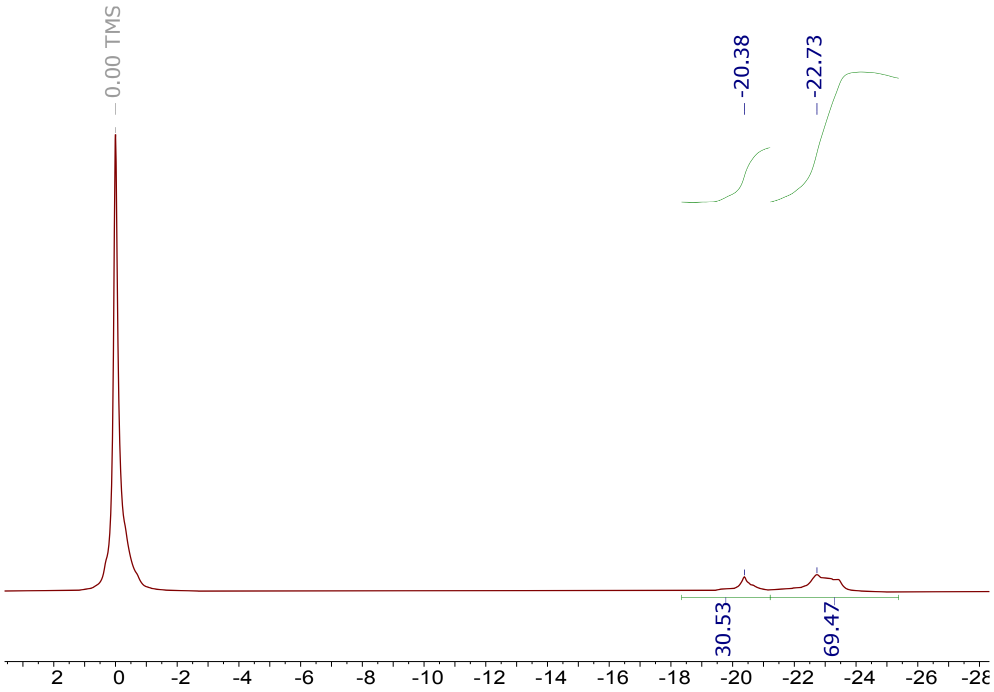

Figure S72. ^{29}Si NMR spectrum of $\mathbf{P_{CN}}$**-OH** obtained from solvent-free AROP at 100 °C with 0.1 equivalents NaOH as initiator. The spectrum was recorded using $Cr(acac)_3$ as a relaxation agent and TMS as a reference.

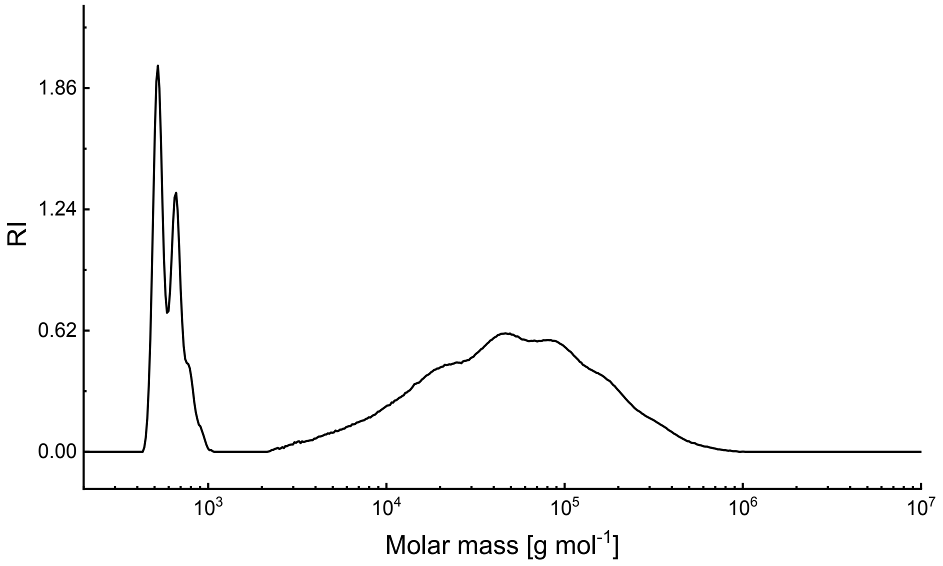

Figure S73. GPC chromatogram of $\mathbf{P_{CN}}$**-OH** obtained from solvent-free AROP at 100 °C with 0.1 equivalents NaOH as initiator. The GPC was done in THF and was calibrated with PS standards.

KOH (40 °C)

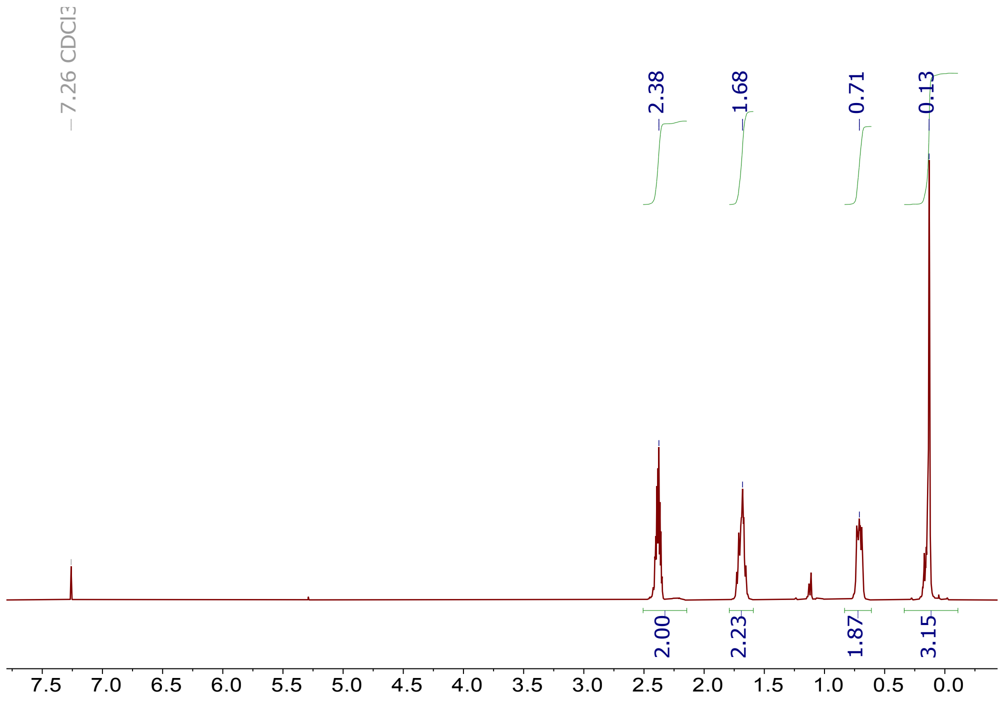

Figure S74. ^{1}H NMR spectrum in $CDCl_3$ of $\mathbf{P_{CN}}$**-OH** obtained from solvent-free AROP at 40 °C with 0.1 equivalents KOH as initiator.

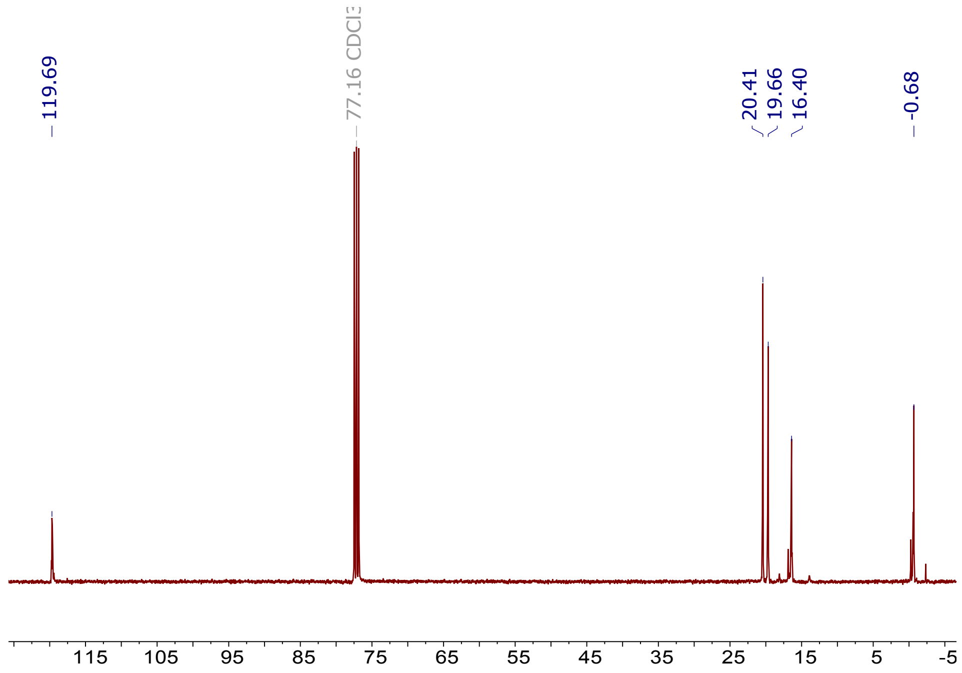

Figure S75. ^{13}C NMR spectrum in $CDCl_3$ of $\mathbf{P_{CN}}$**-OH** obtained from solvent-free AROP at 40 °C with 0.1 equivalents KOH as initiator.

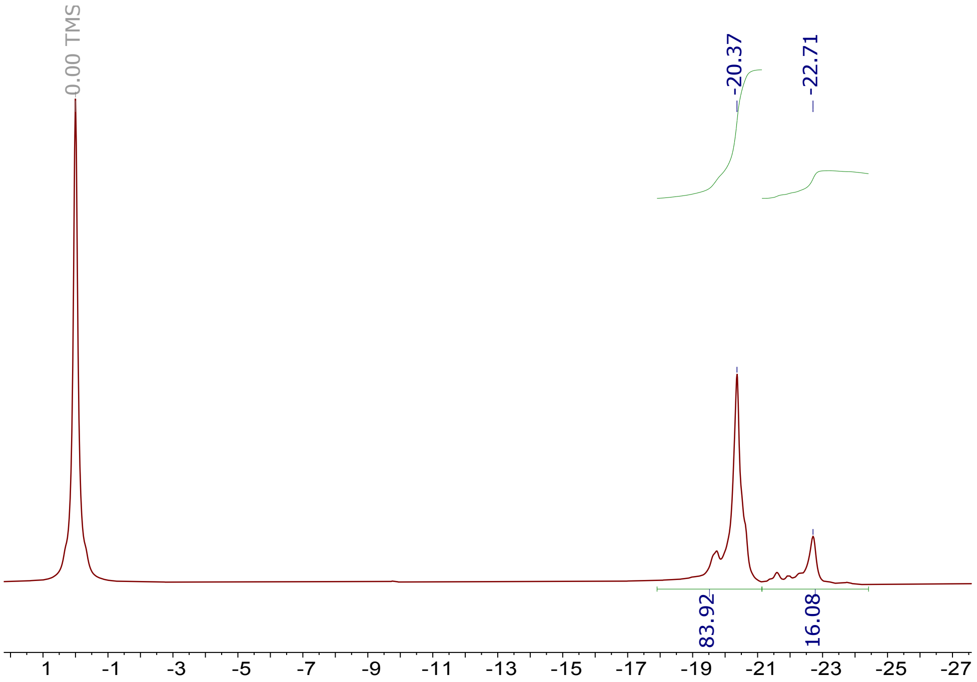

Figure S76. ^{29}Si NMR spectrum of $\mathbf{P_{CN}}$**-OH** obtained from solvent-free AROP at 40 °C with 0.1 equivalents KOH as initiator. The spectrum was recorded using $Cr(acac)_3$ as a relaxation agent and TMS as a reference.

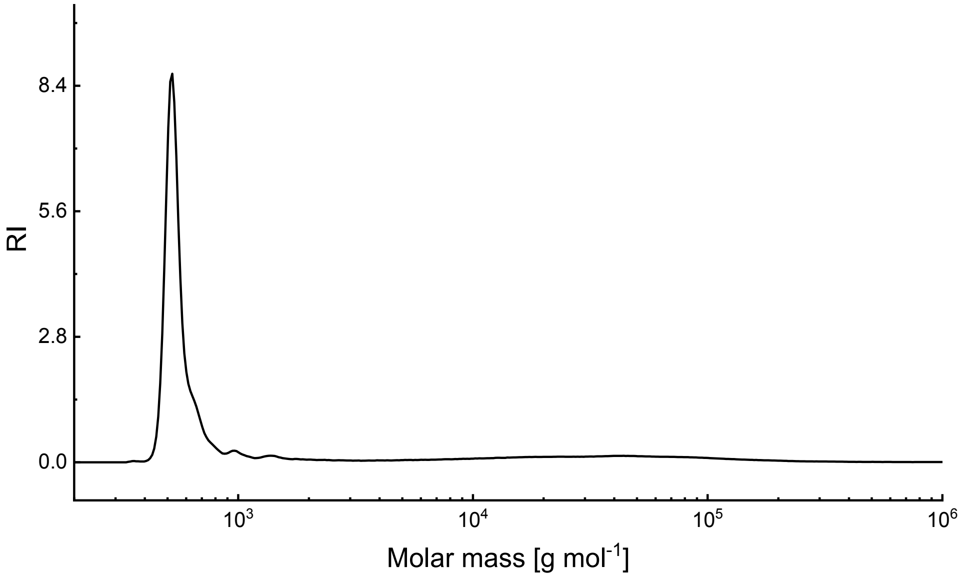

Figure S77. GPC chromatogram of $\mathbf{P_{CN}}$**-OH** obtained from solvent-free AROP at 40 °C with 0.1 equivalents KOH as initiator. The GPC was done in THF and was calibrated with PS standards.

AROP of cycles with TMAH or TBPH as initiator

TMAH (40 °C)

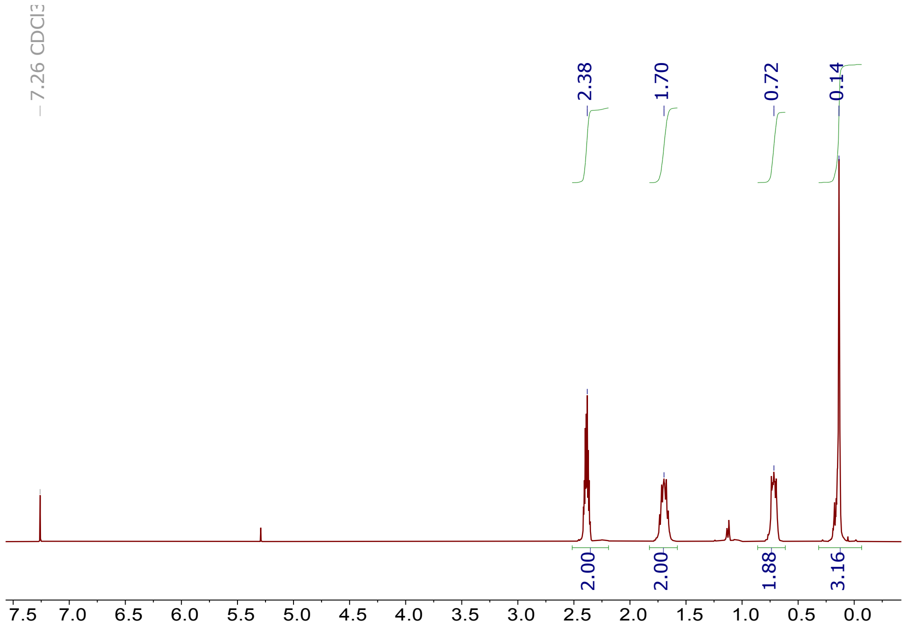

Figure S78. ^{1}H NMR spectrum in $CDCl_3$ of $\mathbf{P_{CN}}$**-OH** obtained from solvent-free AROP at 40 °C with 0.1 equivalents TMAH as initiator.

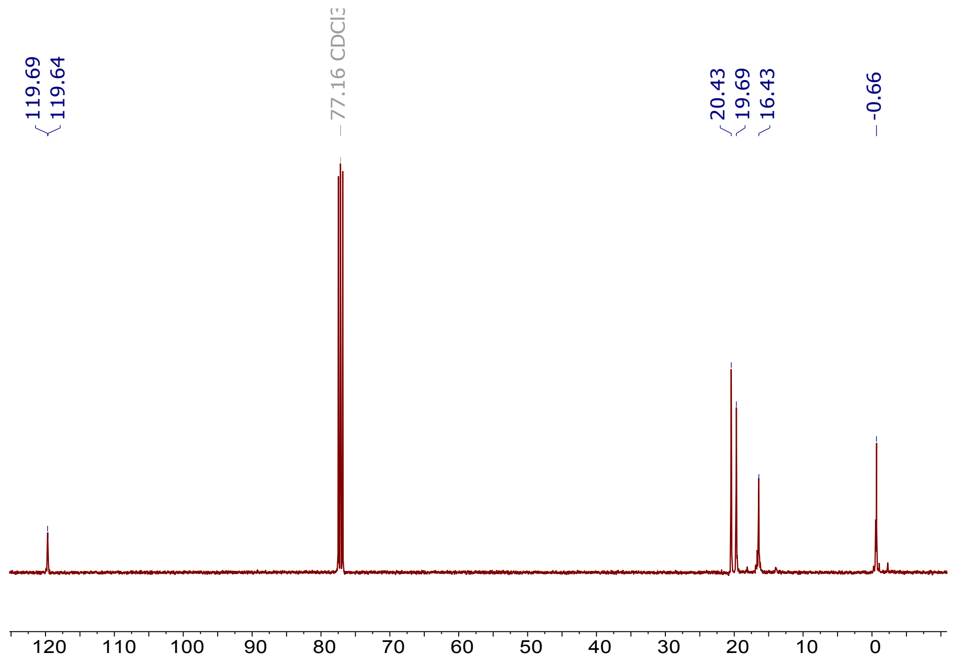

Figure S79. ^{13}C NMR spectrum in $CDCl_3$ of $\mathbf{P_{CN}}$**-OH** obtained from solvent-free AROP at 40 °C with 0.1 equivalents TMAH as initiator.

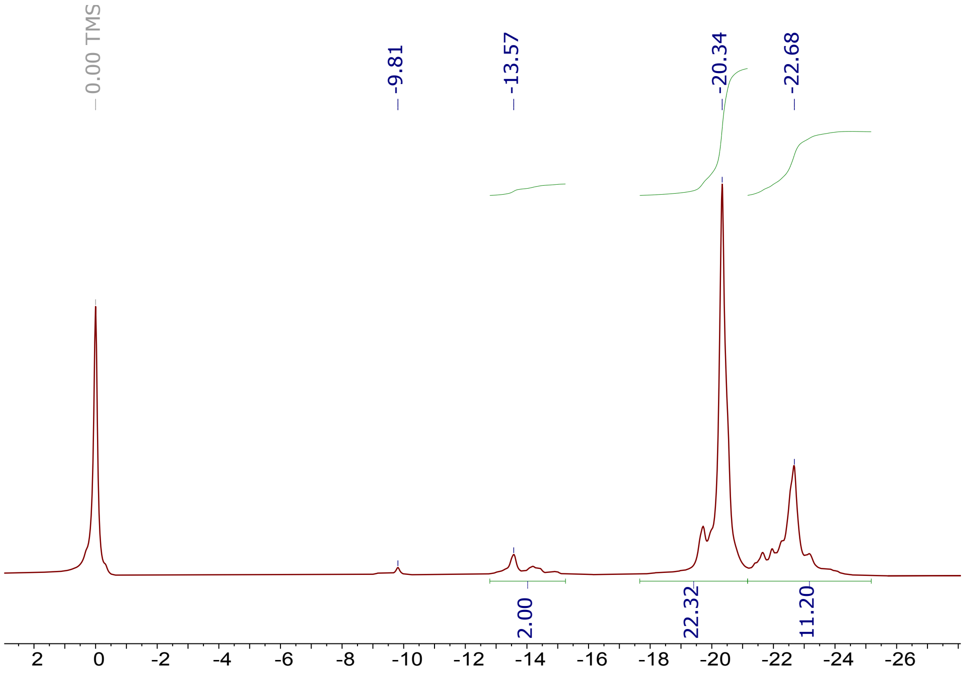

Figure S80. ^{29}Si NMR spectrum of $\mathbf{P_{CN}}$**-OH** obtained from solvent-free AROP at 40 °C with 0.1 equivalents TMAH as initiator. The spectrum was recorded using $Cr(acac)_3$ as a relaxation agent and TMS as a reference.

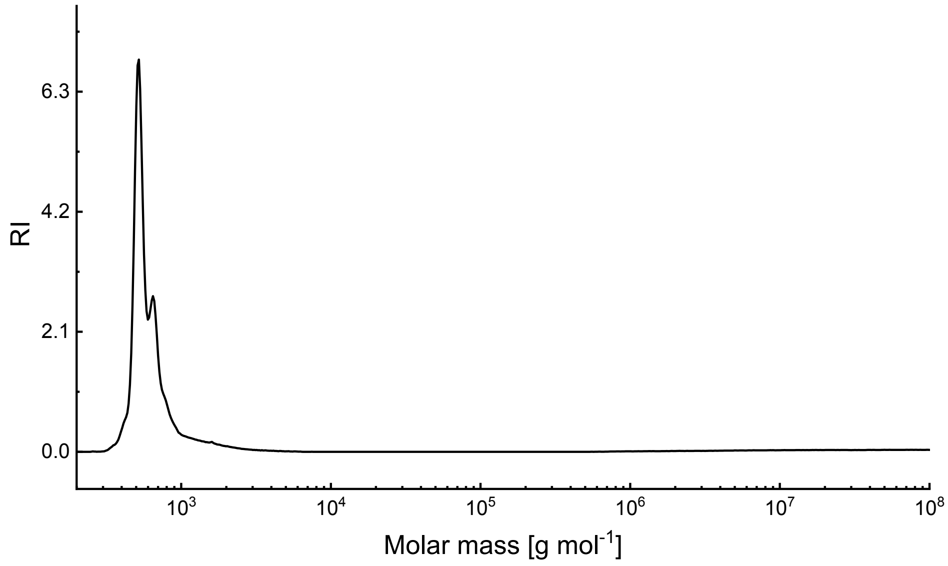

Figure S81. GPC chromatogram of **P_{CN}-OH** obtained from solvent-free AROP at 40 °C with 0.1 equivalents TMAH as initiator. The GPC was done in THF and was calibrated with PS standards.

TBPH (40 °C)

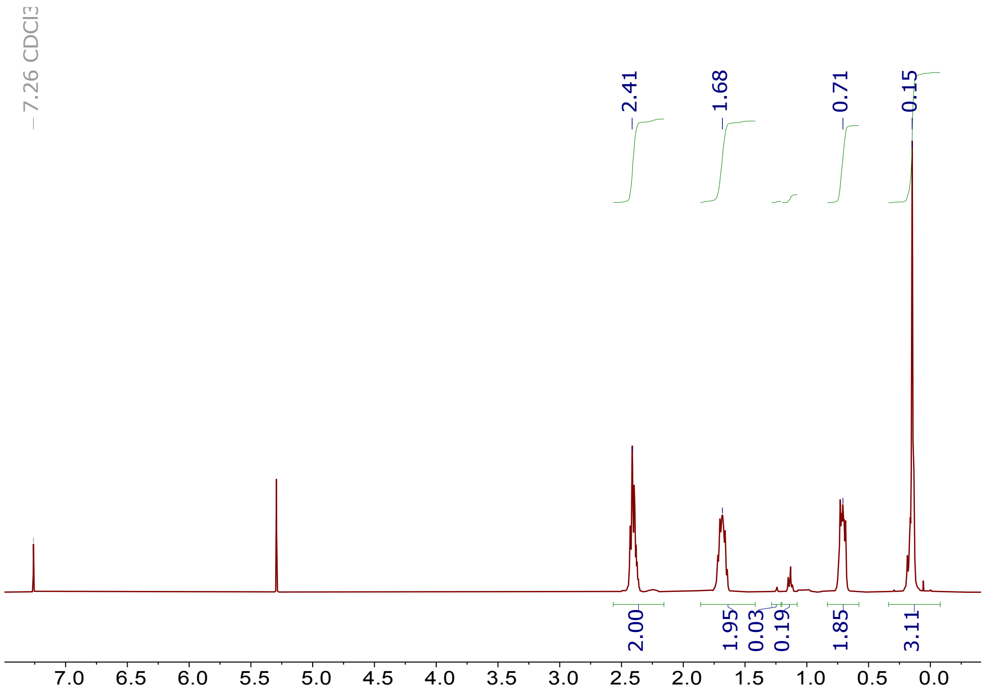

Figure S82. ^{1}H NMR spectrum in $CDCl_3$ of **P_{CN}-OH** obtained from solvent-free AROP at 40 °C with 0.1 equivalents TBPH as initiator.

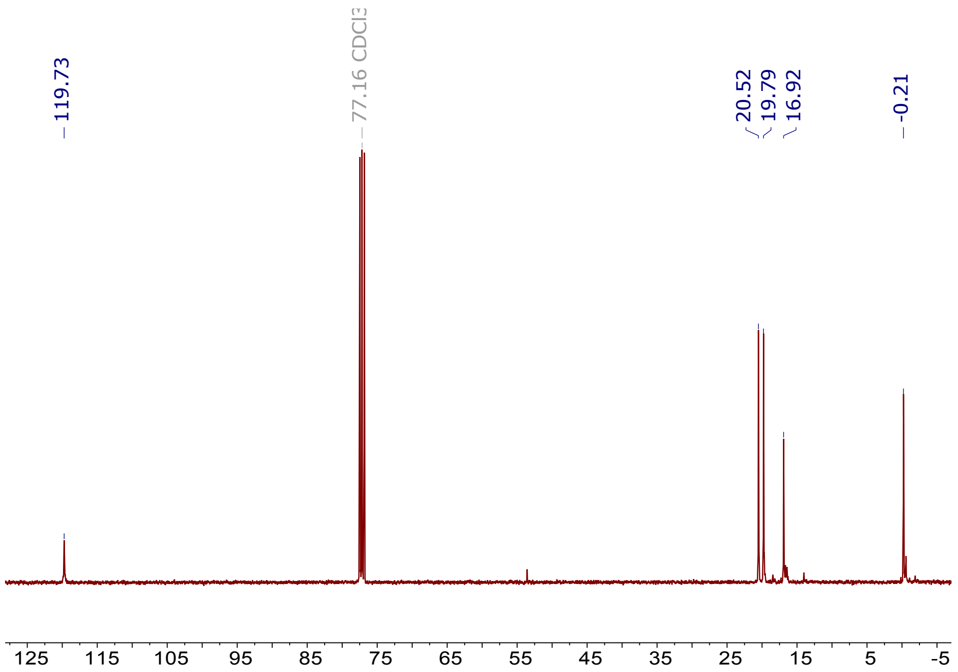

Figure S83. ^{13}C NMR spectrum in $CDCl_3$ of $\mathbf{P_{CN}}$**-OH** obtained from solvent-free AROP at 40 °C with 0.1 equivalents TBPH as initiator.

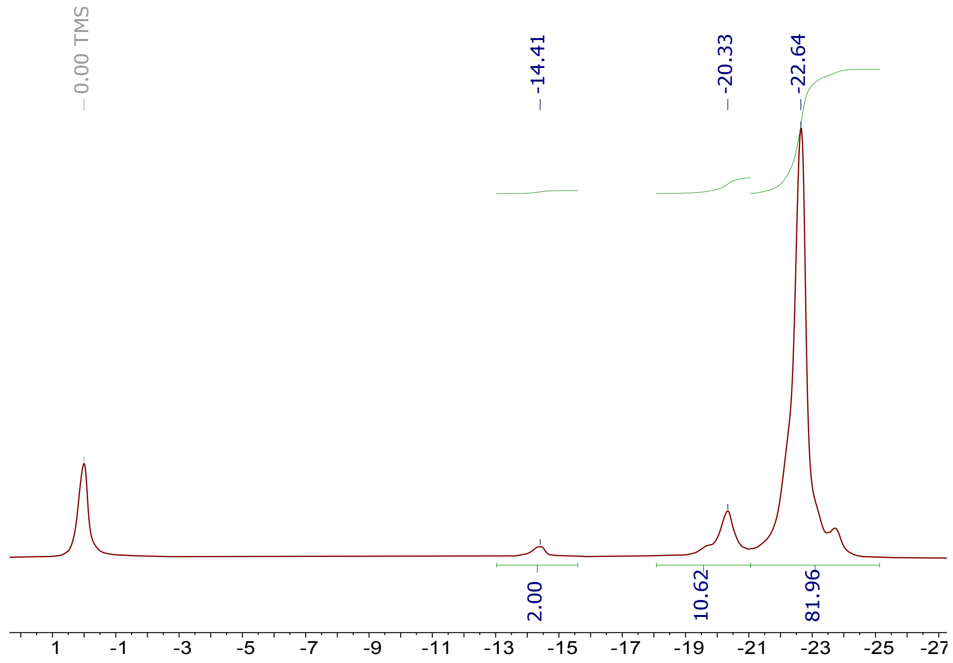

Figure S84. ^{29}Si NMR spectrum of $\mathbf{P_{CN}}$**-OH** obtained from solvent-free AROP at 40 °C with 0.1 equivalents TBPH as initiator. The spectrum was recorded using $Cr(acac)_3$ as a relaxation agent and TMS as a reference.

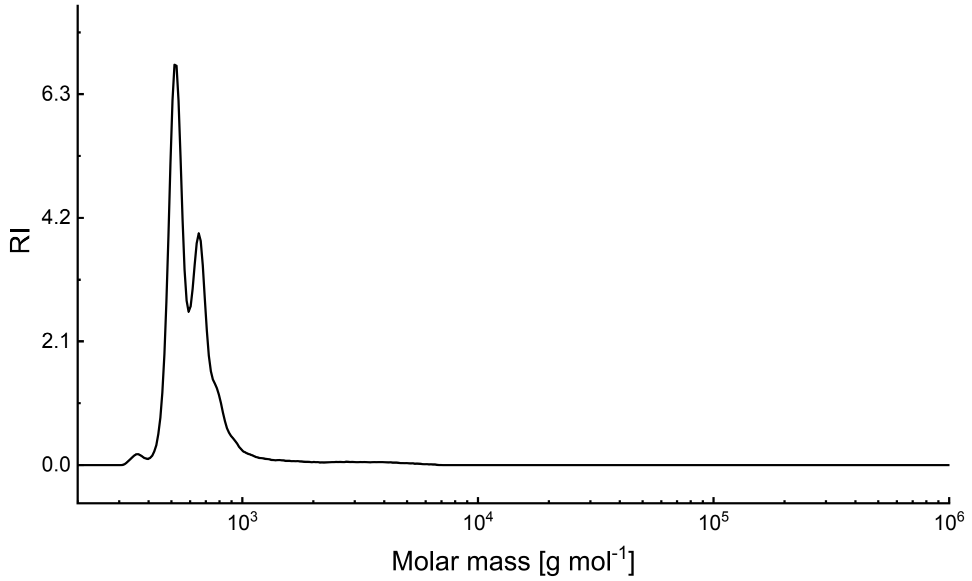

Figure S85. GPC chromatogram of $\mathbf{P_{CN}}$**-OH** obtained from solvent-free AROP at 40 °C with 0.1 equivalents TBPH as initiator. The GPC was done in THF and was calibrated with PS standards.

TBPH (60 °C)

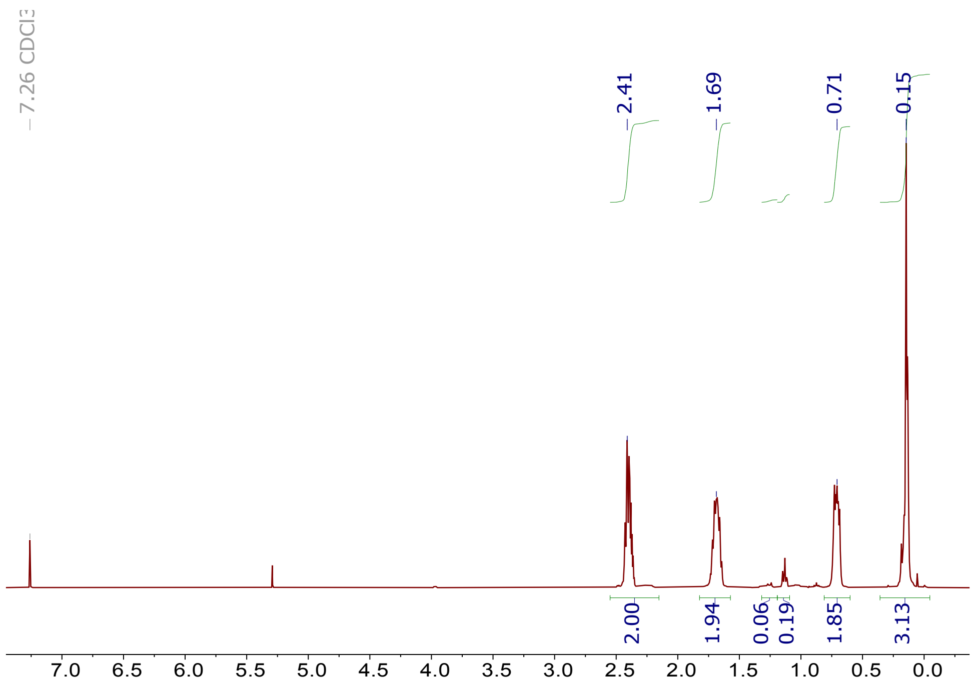

Figure S86. ^{1}H NMR spectrum in $CDCl_3$ of $\mathbf{P_{CN}}$**-OH** obtained from solvent-free AROP at 60 °C with 0.1 equivalents TBPH as initiator.

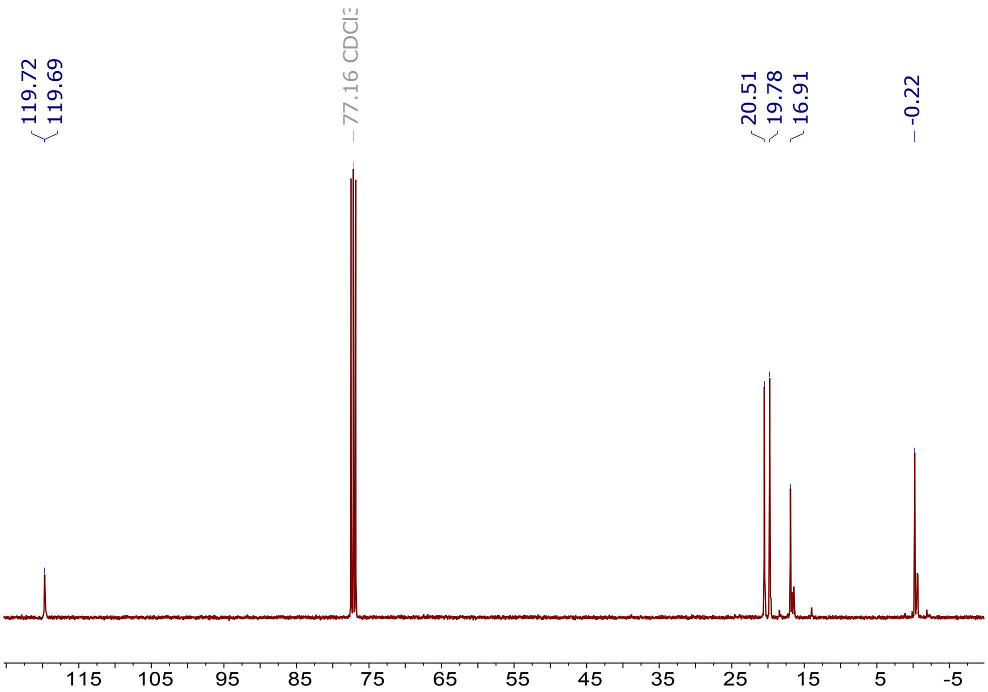

Figure S87. ^{13}C NMR spectrum in $CDCl_3$ of $\mathbf{P_{CN}}$**-OH** obtained from solvent-free AROP at 60 °C with 0.1 equivalents TBPH as initiator.

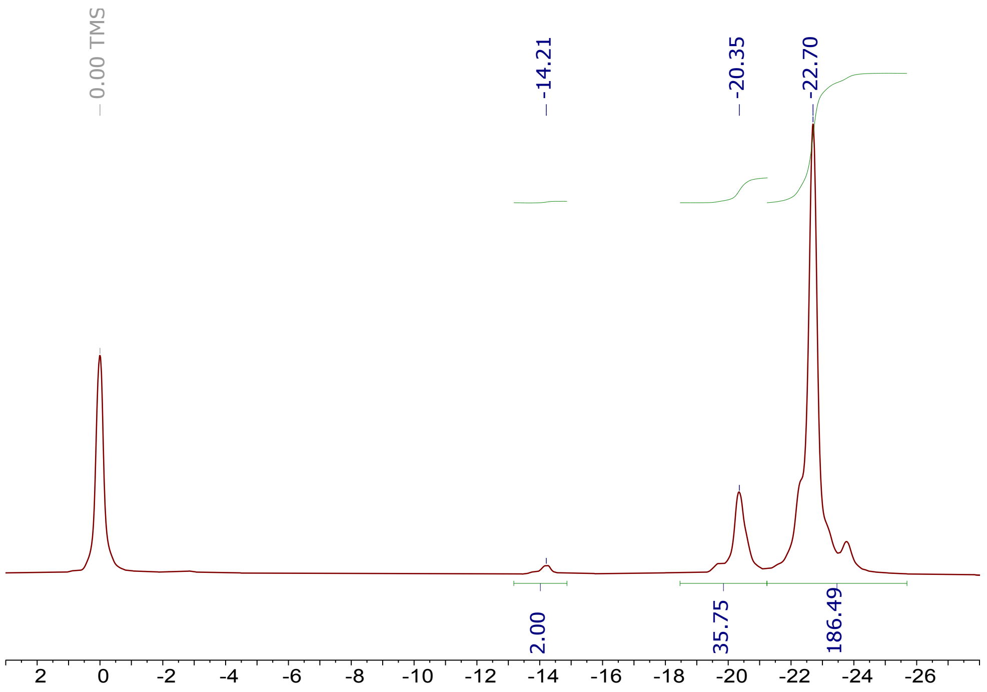

Figure S88. ^{29}Si NMR spectrum of $\mathbf{P_{CN}}$**-OH** obtained from solvent-free AROP at 60 °C with 0.1 equivalents TBPH as initiator. The spectrum was recorded using $Cr(acac)_3$ as a relaxation agent and TMS as a reference.

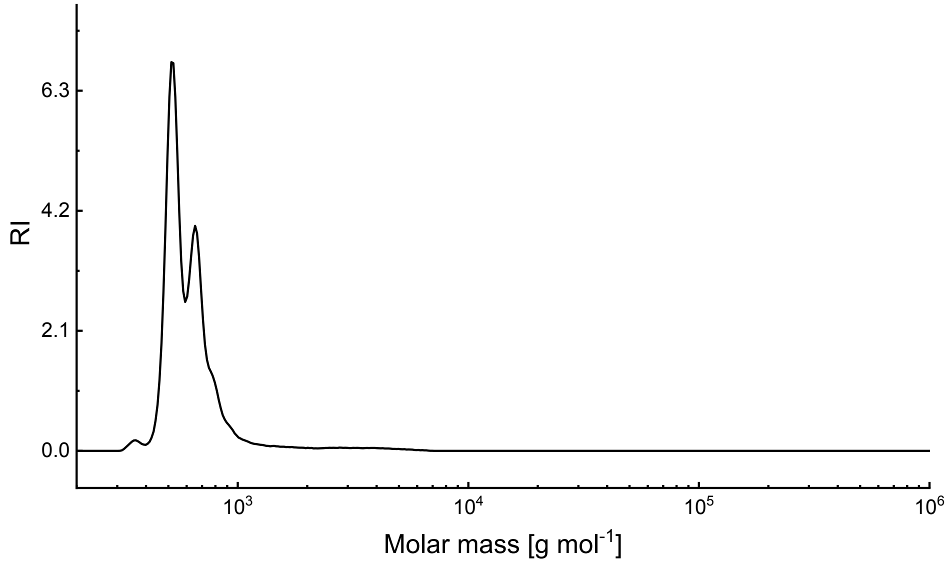

Figure S89. GPC chromatogram of **P_{CN}-OH** obtained from solvent-free AROP at 60 °C with 0.1 equivalents TBPH as initiator. The GPC was done in THF and was calibrated with PS standards.

TBPH (80 °C)

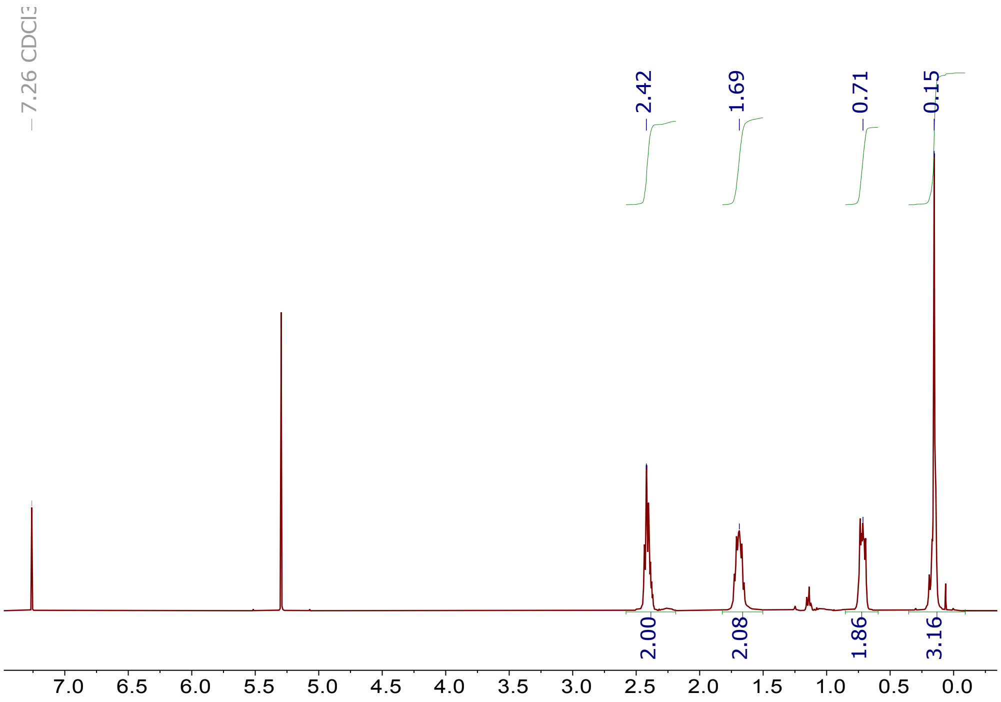

Figure S90. ^{1}H NMR spectrum in $CDCl_3$ of **P_{CN}-OH** obtained from solvent-free AROP at 80 °C with 0.1 equivalents TBPH as initiator.

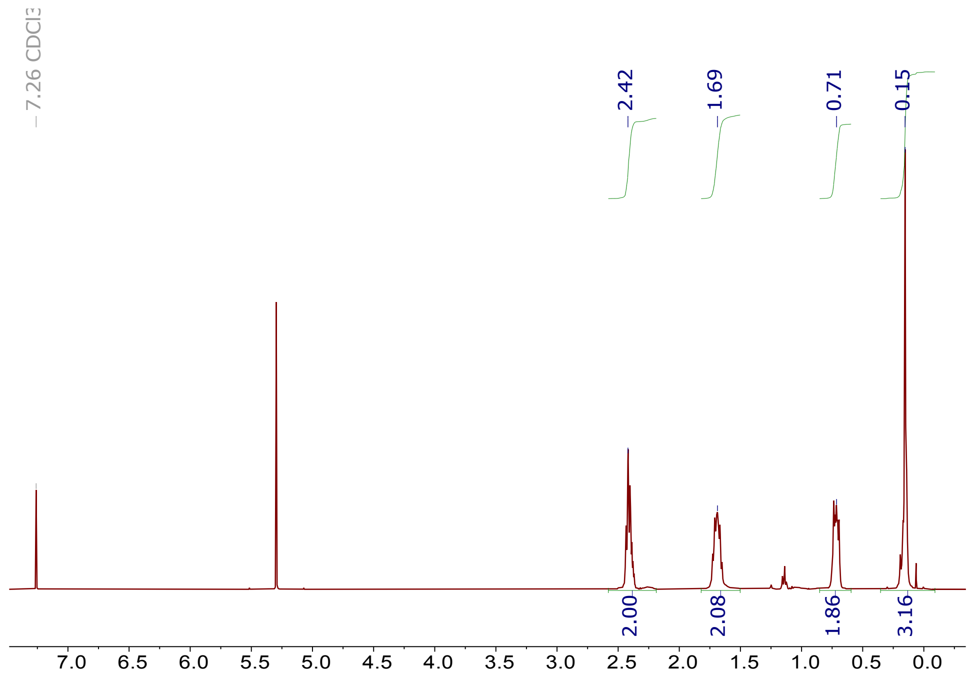

Figure S91. ^{13}C NMR spectrum in $CDCl_3$ of $\mathbf{P_{CN}}$**-OH** obtained from solvent-free AROP at 80 °C with 0.1 equivalents TBPH as initiator.

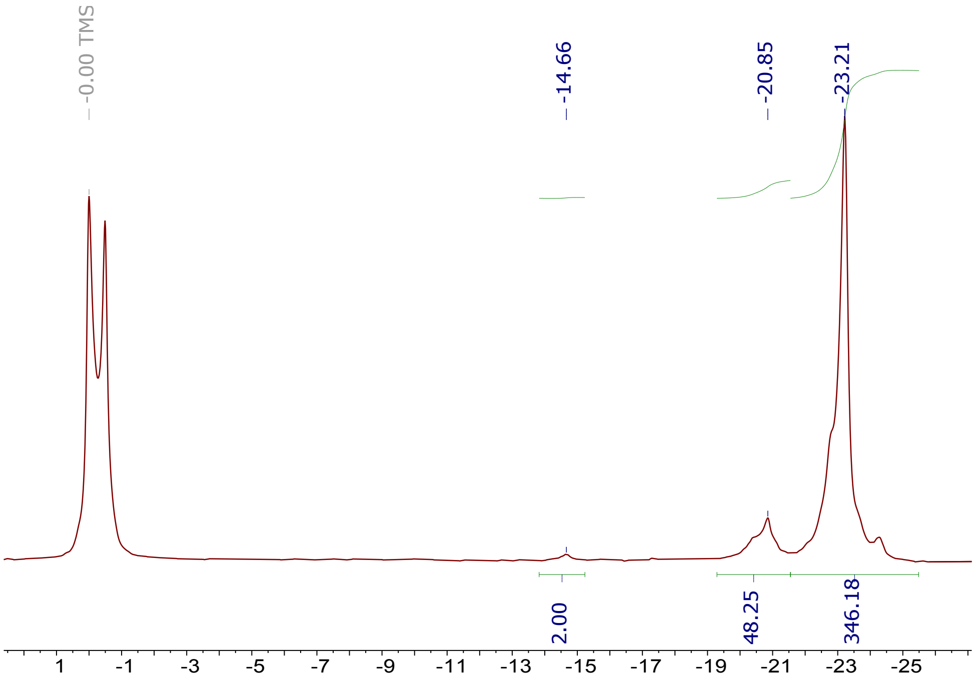

Figure S92. ^{29}Si NMR spectrum of $\mathbf{P_{CN}}$**-OH** obtained from solvent-free AROP at 80 °C with 0.1 equivalents TBPH as initiator. The spectrum was recorded using $Cr(acac)_3$ as a relaxation agent and TMS as a reference.

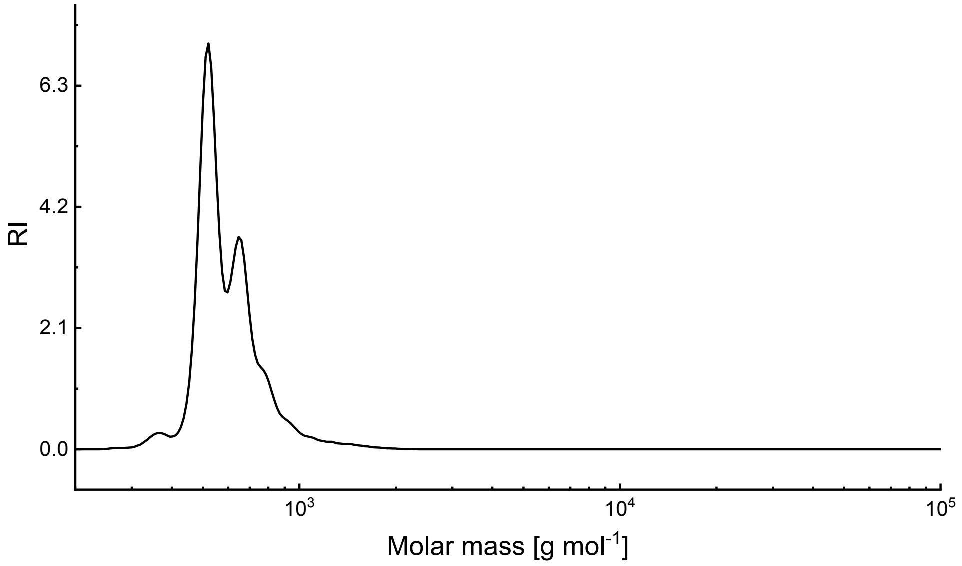

Figure S93. GPC chromatogram of **P_{CN}-OH** obtained from solvent-free AROP at 80 °C with 0.1 equivalents TBPH as initiator. The GPC was done in THF and was calibrated with PS standards.

TBPH (0.01 eq, 80 °C)

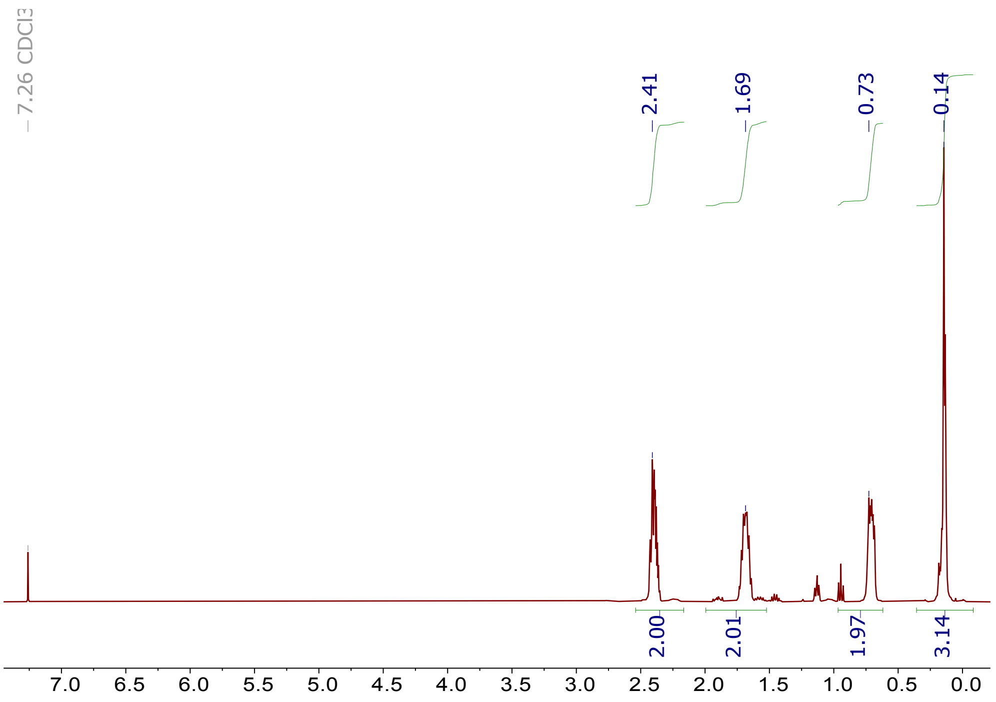

Figure S94. ^{1}H NMR spectrum in $CDCl_3$ of **P_{CN}-OH** obtained from solvent-free AROP at 80 °C with 0.01 equivalents TBPH as initiator.

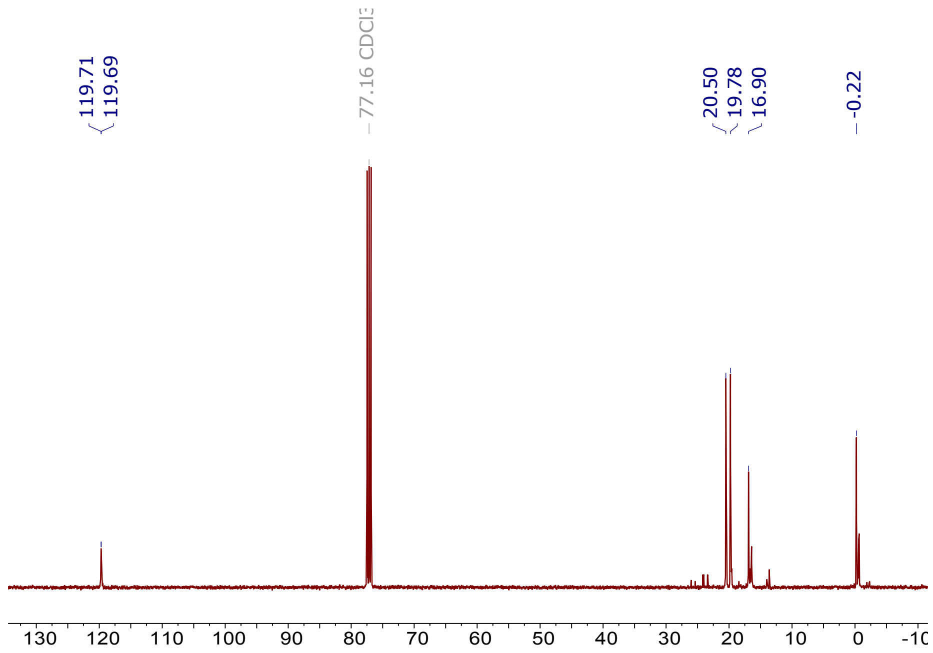

Figure S95. ^{13}C NMR spectrum in $CDCl_3$ of **P_{CN}-OH** obtained from solvent-free AROP at 80 °C with 0.01 equivalents TBPH as initiator.

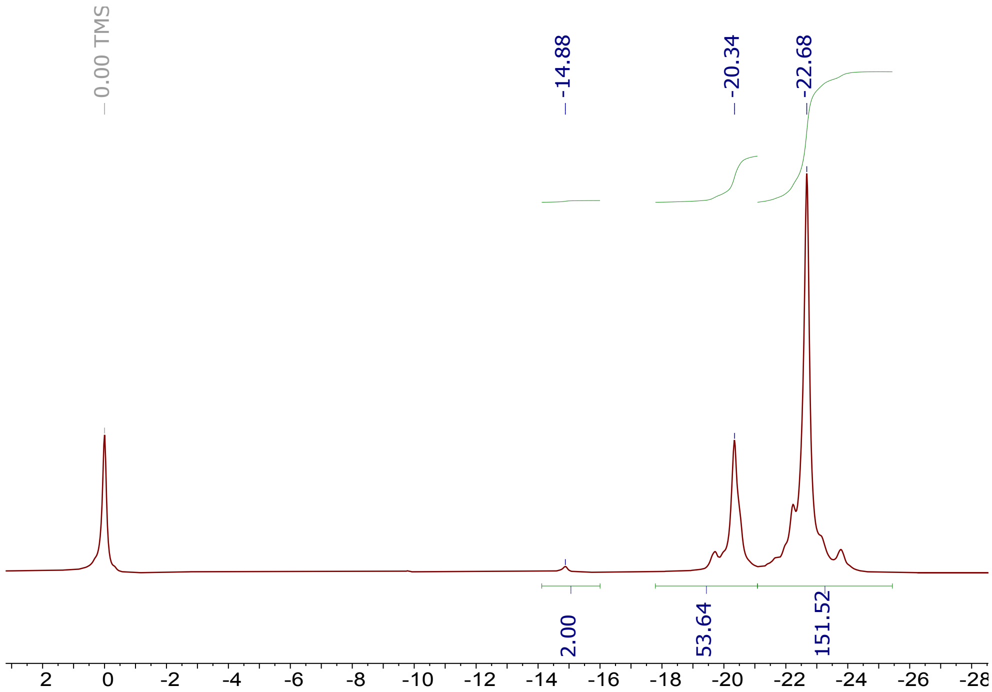

Figure S96. ^{29}Si NMR spectrum of **P_{CN}-OH** obtained from solvent-free AROP at 80 °C with 0.01 equivalents TBPH as initiator. The spectrum was recorded using $Cr(acac)_3$ as a relaxation agent and TMS as a reference.

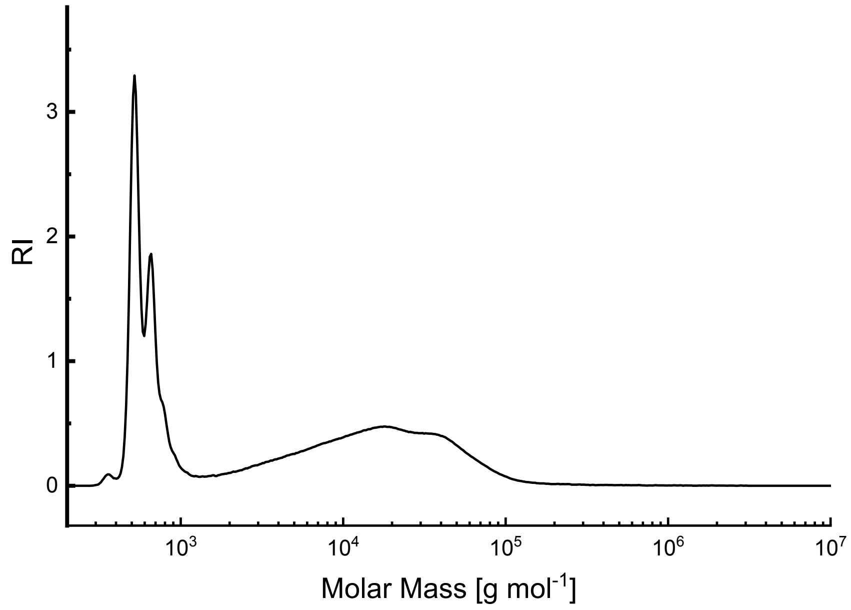

Figure S97. GPC chromatogram of $\mathbf{P_{CN}}$**-OH** obtained from solvent-free AROP at 80 °C with 0.01 equivalents TBPH as initiator. The GPC was done in THF and was calibrated with PS standards.

TBPH (0.001 eq, 80 °C)

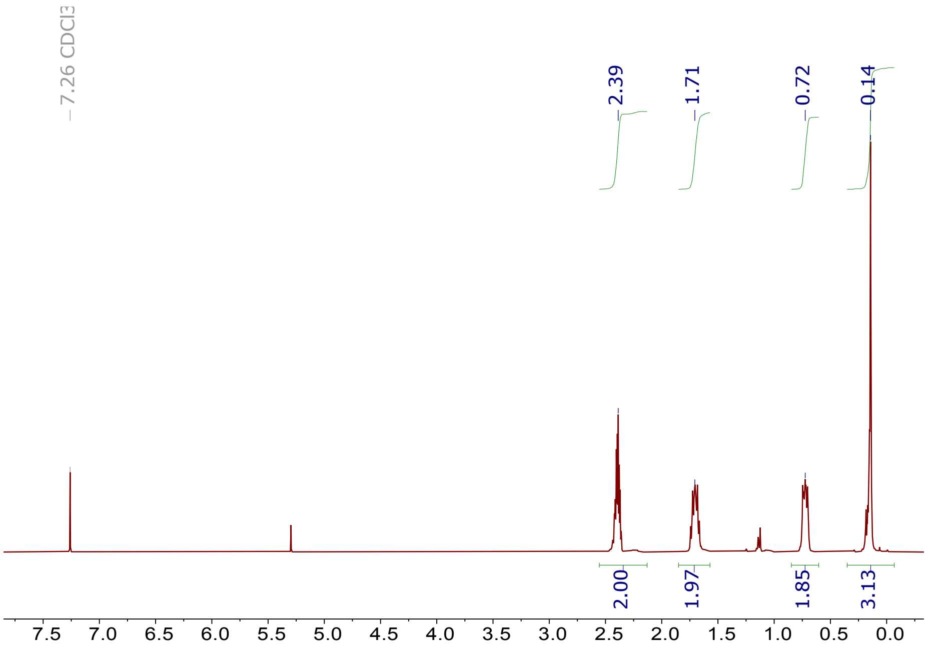

Figure S98. ^{1}H NMR spectrum in $CDCl_3$ of $\mathbf{P_{CN}}$**-OH** obtained from solvent-free AROP at 80 °C with 0.001 equivalents TBPH as initiator.

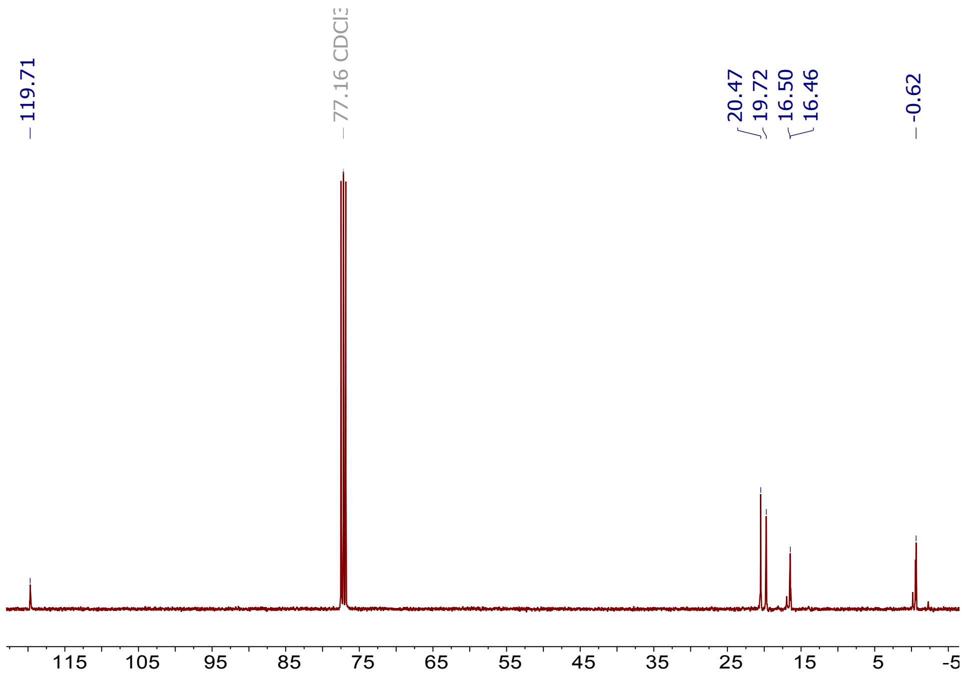

Figure S99. ^{13}C NMR spectrum in $CDCl_3$ of **P_{CN}-OH** obtained from solvent-free AROP at 80 °C with 0.001 equivalents TBPH as initiator.

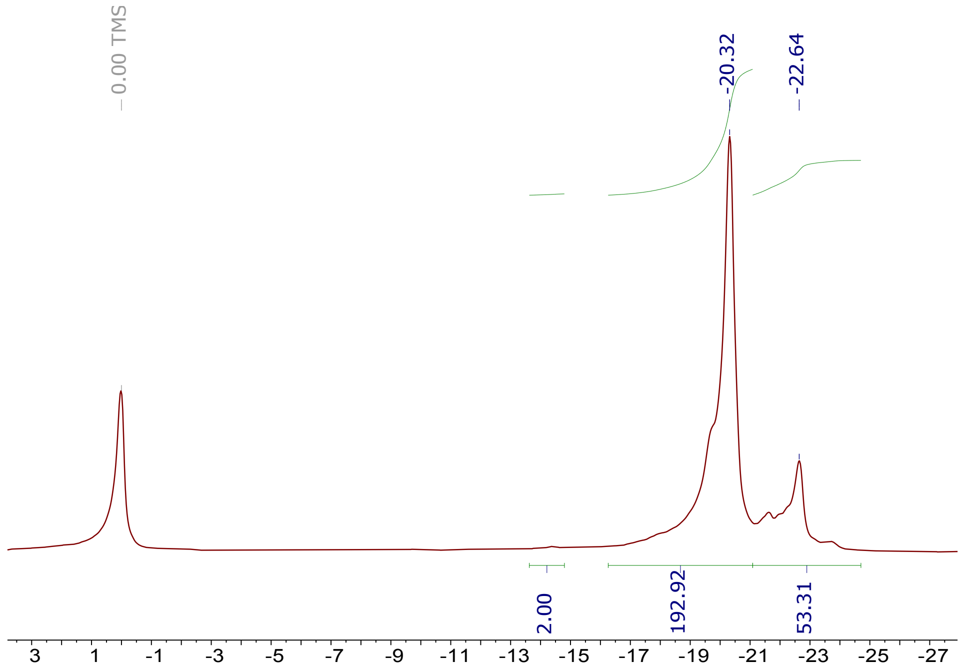

Figure S100. ^{29}Si NMR spectrum of **P_{CN}-OH** obtained from solvent-free AROP at 80 °C with 0.001 equivalents TBPH as initiator. The spectrum was recorded using $Cr(acac)_3$ as a relaxation agent and TMS as a reference.

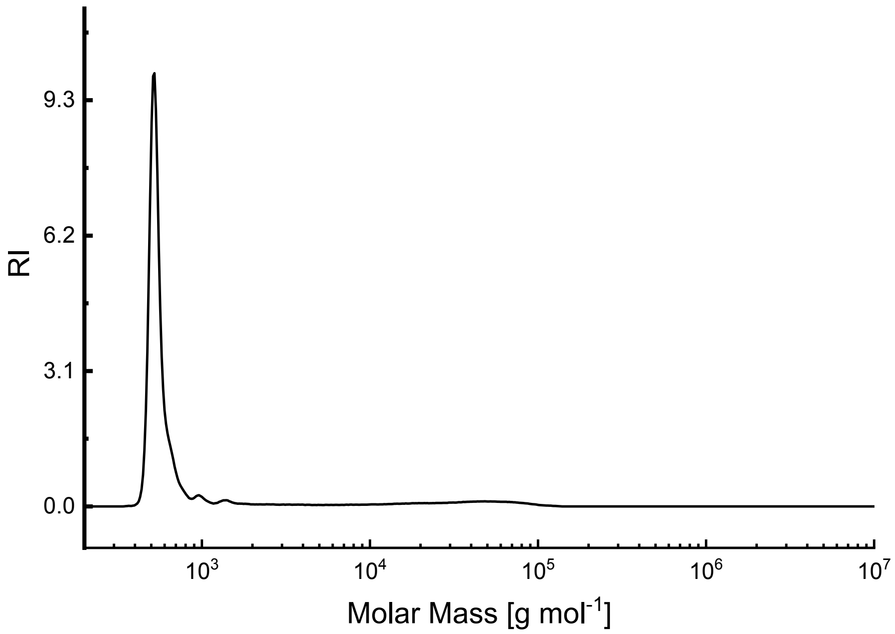

Figure S101. GPC chromatogram of **P_{CN}-OH** obtained from solvent-free AROP at 80 °C with 0.001 equivalents TBPH as initiator. The GPC was done in THF and was calibrated with PS standards.

TBPH (100 °C)

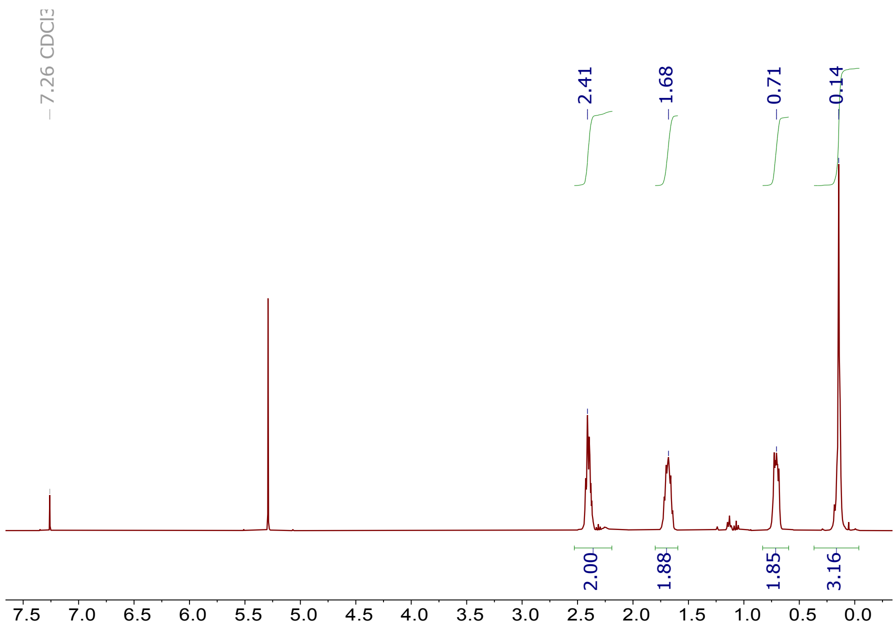

Figure S102. ^{1}H NMR spectrum in $CDCl_3$ of **P_{CN}-OH** obtained from solvent-free AROP at 100 °C with 0.1 equivalents TBPH as initiator.

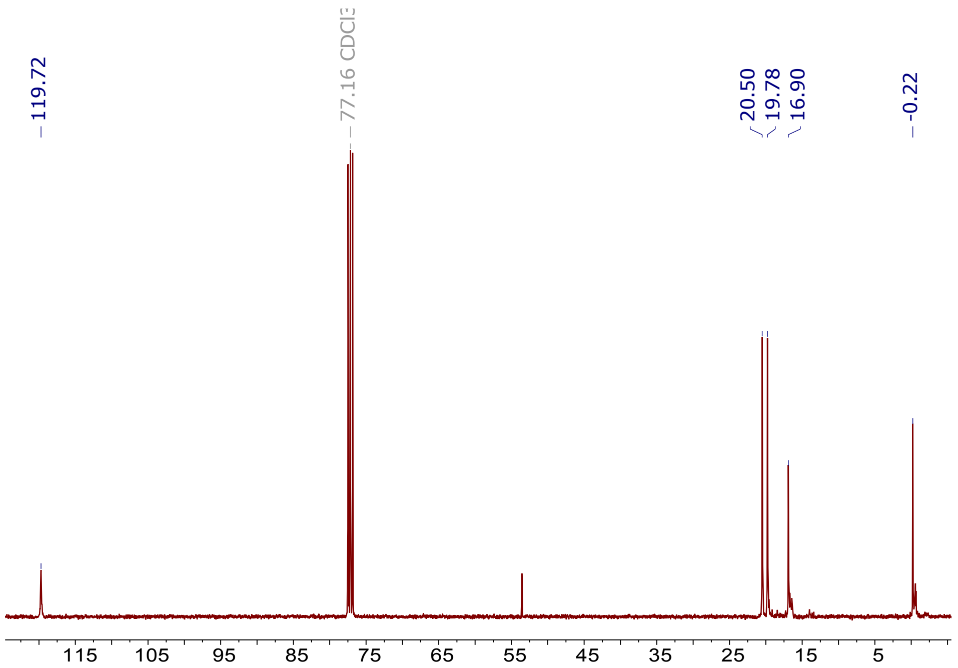

Figure S103. ^{13}C NMR spectrum in $CDCl_3$ of $\mathbf{P_{CN}}$**-OH** obtained from solvent-free AROP at 100 °C with 0.1 equivalents TBPH as initiator.

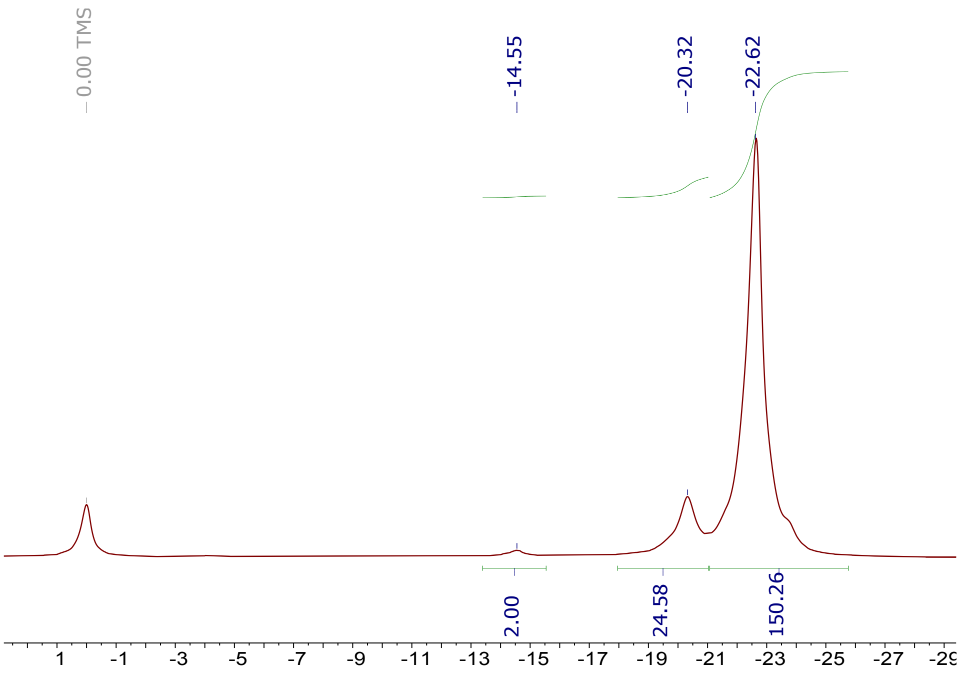

Figure S104. ^{29}Si NMR spectrum of $\mathbf{P_{CN}}$**-OH** obtained from solvent-free AROP at 100 °C with 0.1 equivalents TBPH as initiator. The spectrum was recorded using $Cr(acac)_3$ as a relaxation agent and TMS as a reference.

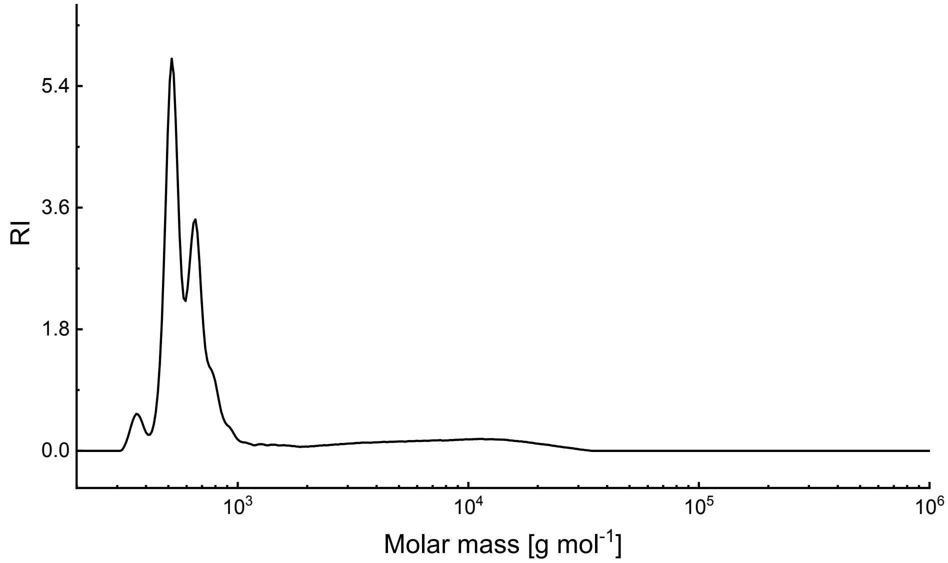

Figure S105. GPC chromatogram of $\mathbf{P_{CN}}$**-OH** obtained from solvent-free AROP at 100 °C with 0.1 equivalents TBPH as initiator. The GPC was done in THF and was calibrated with PS standards.

Post-polymerization end-functionalization with cyclic silazane

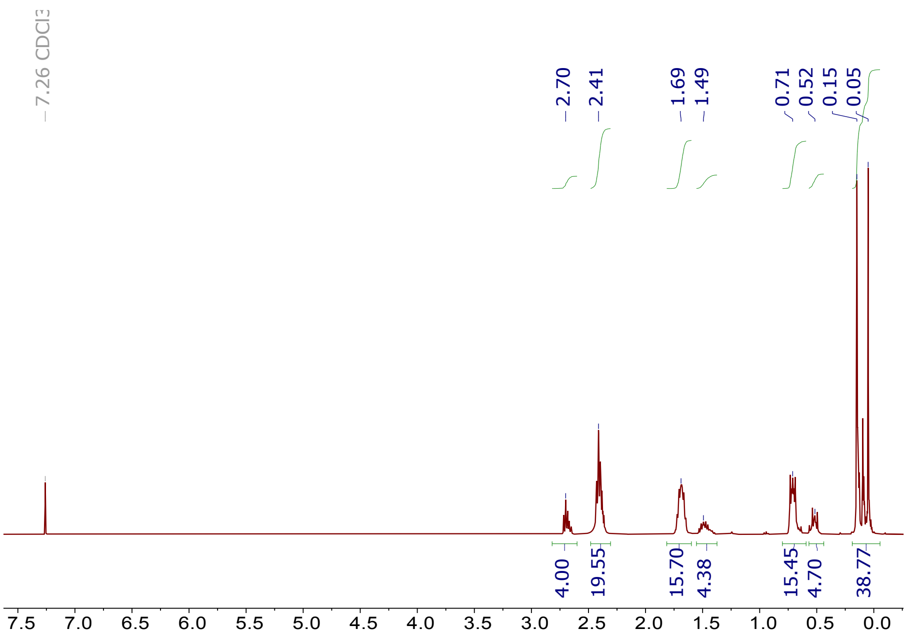

Figure S106. ^{1}H NMR spectrum of polymer ($\mathbf{P_{CN}}$**-NH$_2$**) obtained from end-functionalization of short chains in $CDCl_3$.

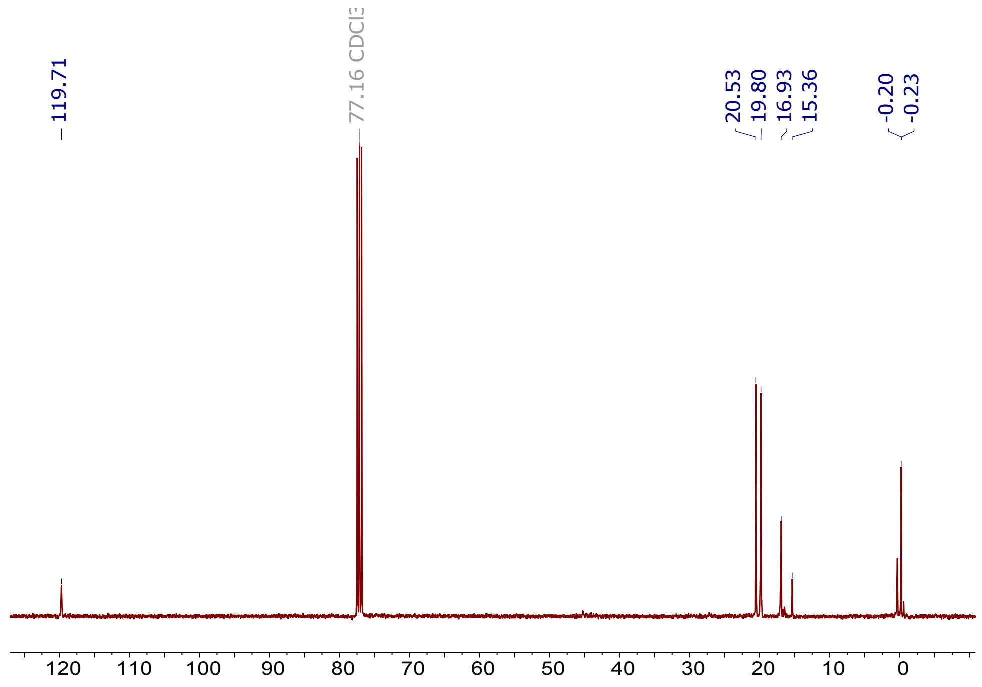

Figure S107. ^{13}C NMR spectrum of polymer (**P_{CN}-NH_2**) obtained from end-functionalization of a mixture of chains and cycles in $CDCl_3$.

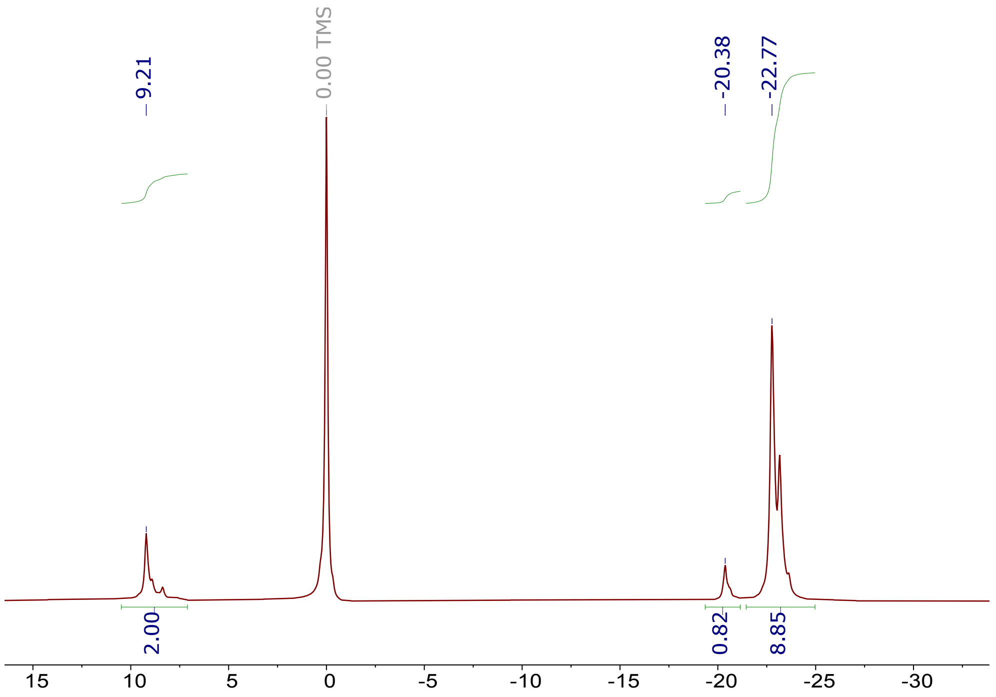

Figure S108. ^{29}Si NMR spectrum of polymer (**P_{CN}-NH_2**) obtained from end-functionalization of a mixture of chains and cycles was recorded using $Cr(acac)_3$ as a relaxation agent and TMS as a reference.

Post-polymerization end-functionalization with vinylchlorosilane

Endfunctionalization of **P_{CN}-OH** with 18 RU's:

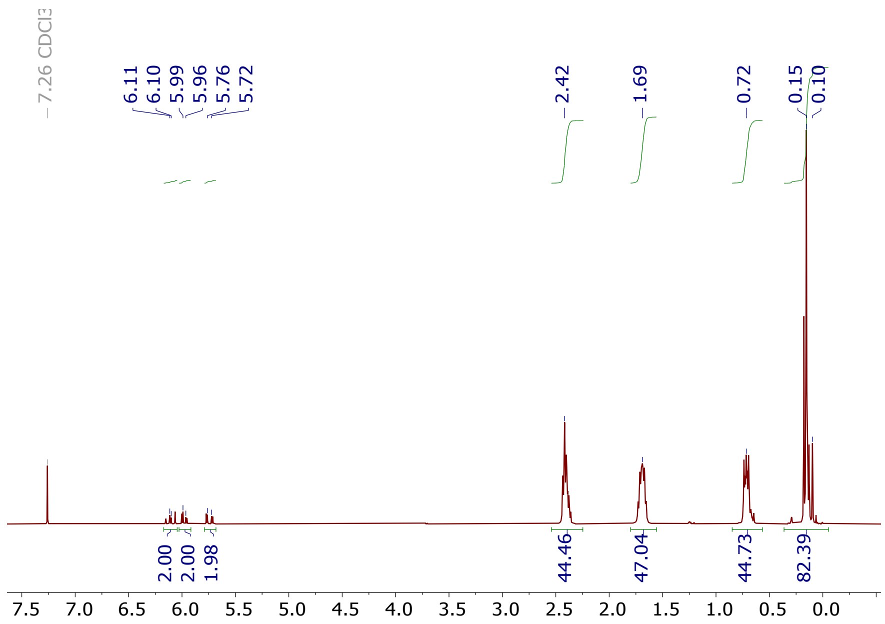

Figure S109. ^{1}H NMR spectrum of polymer (**P_{CN}-V**) obtained from end-functionalization of short chains in $CDCl_3$.

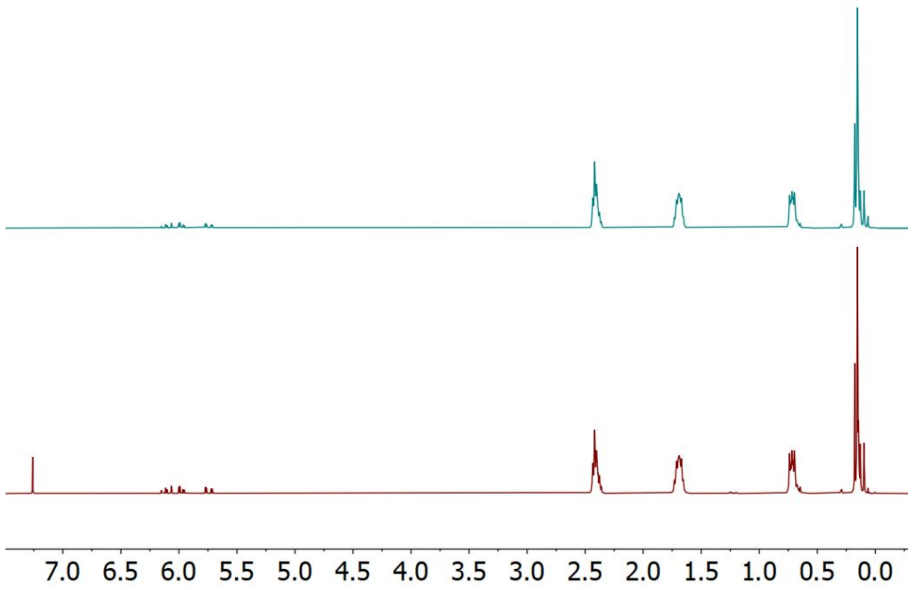

Figure S110. Diffusion-edited NMR of (**P_{CN}-V**) obtained from end-functionalization of short chains (top) and 1H NMR of (**P_{CN}-V**) stacked (bottom).

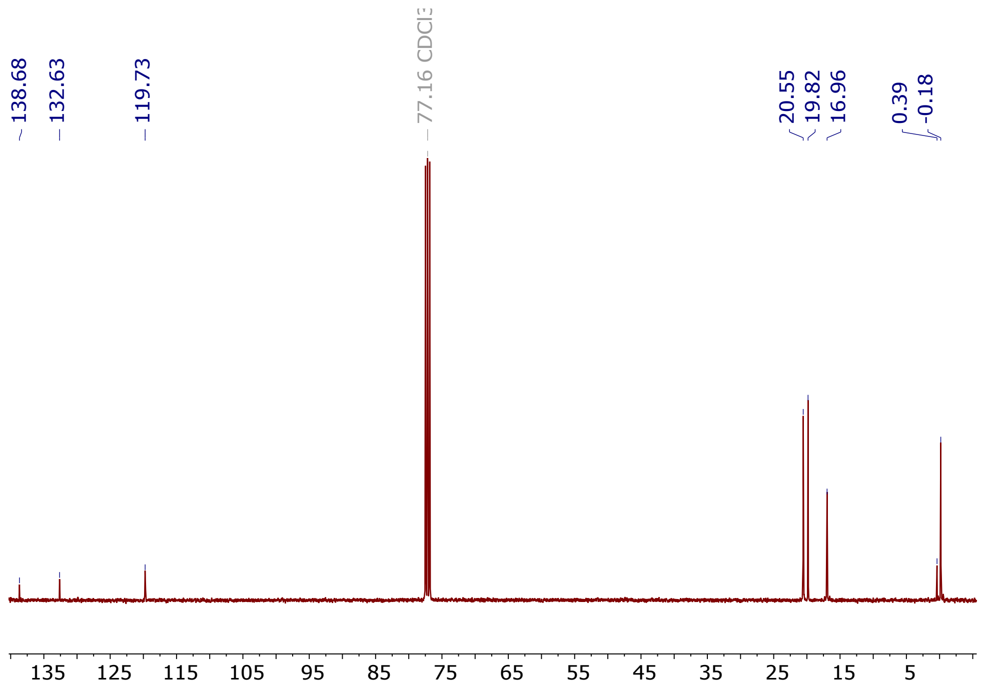

Figure S111. ^{13}C NMR spectrum of polymer (**P$_{CN}$-V**) obtained from end-functionalization of short chains in $CDCl_3$.

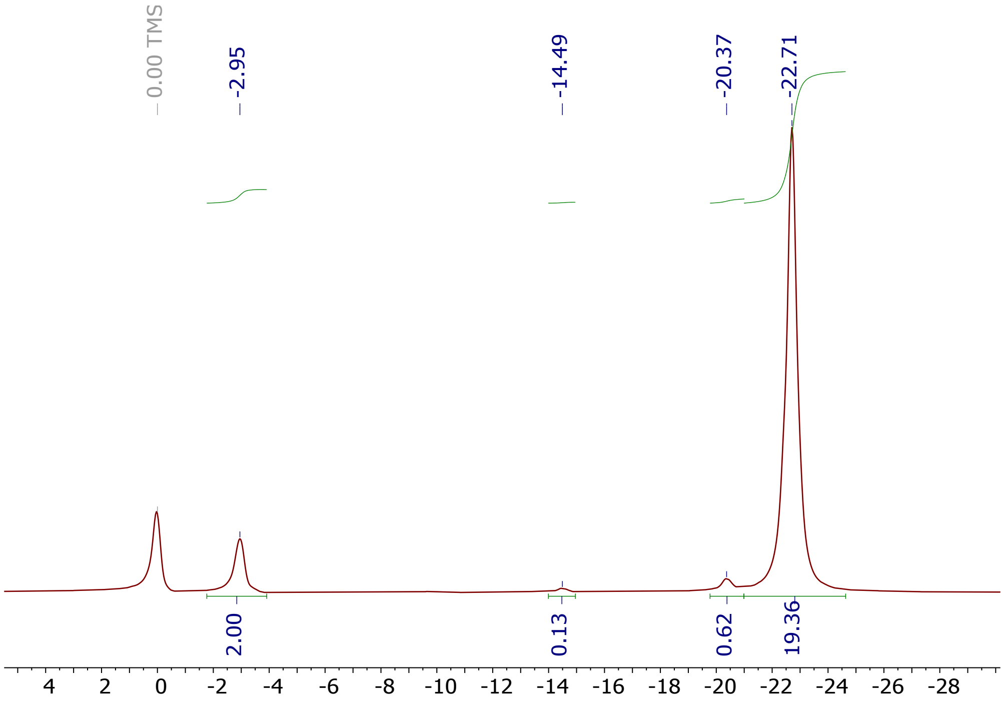

Figure S112. ^{29}Si NMR spectrum of polymer (**P$_{CN}$-V**) obtained from end-functionalization of short chains The spectrum was recorded using $Cr(acac)_3$ as a relaxation agent and TMS as a reference.

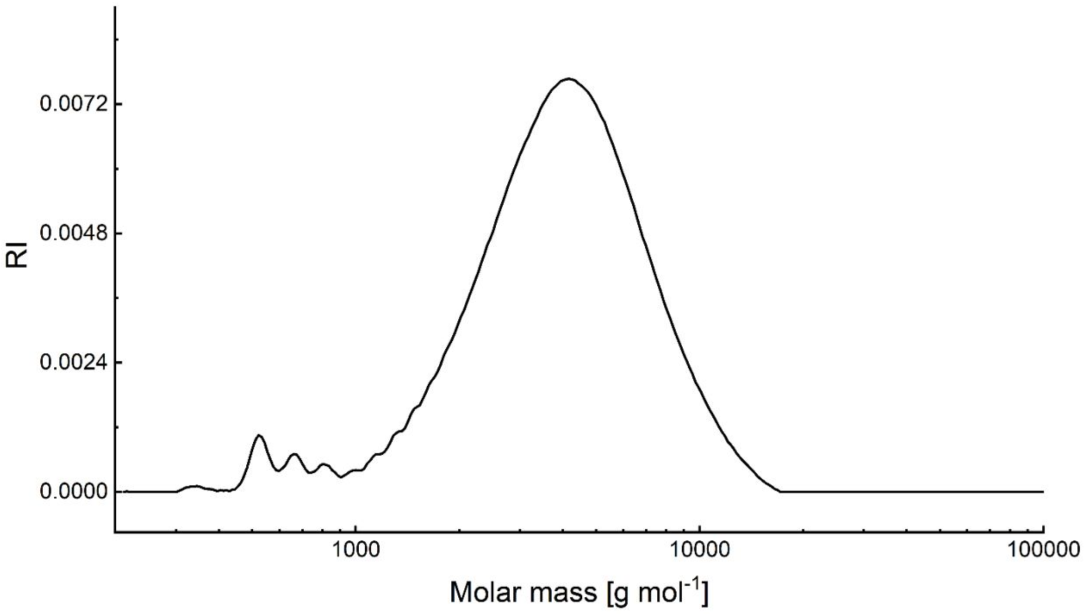

Figure S113. GPC chromatogram of polymer ($\mathbf{P_{CN}}$-**V**) obtained from end-functionalization of short chains. The GPC was recorded in THF and was calibrated with PS standards.

Endfunctionalization of P_{CN}-OH with 37 RU's

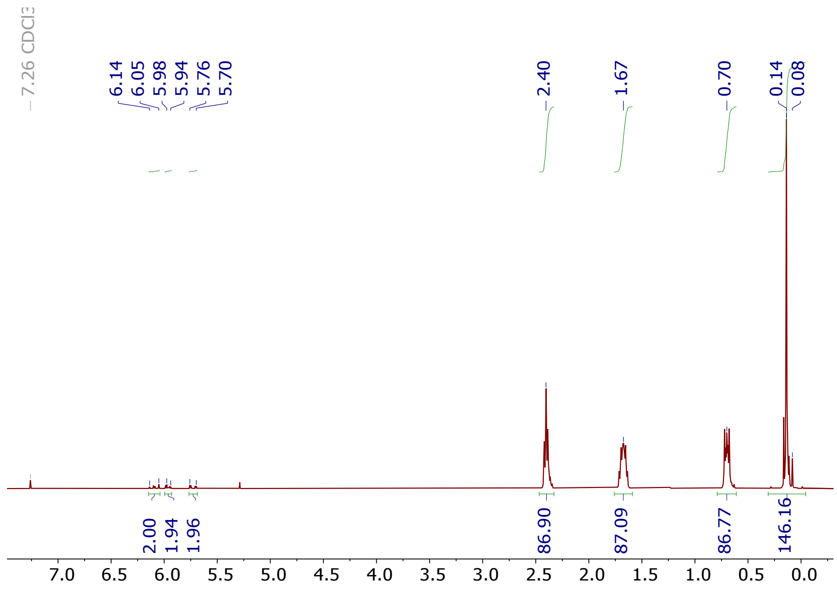

Figure S114. ^{1}H NMR spectrum of polymer ($\mathbf{P_{CN}}$-**V**) obtained from end-functionalization of long chains in $CDCl_3$.

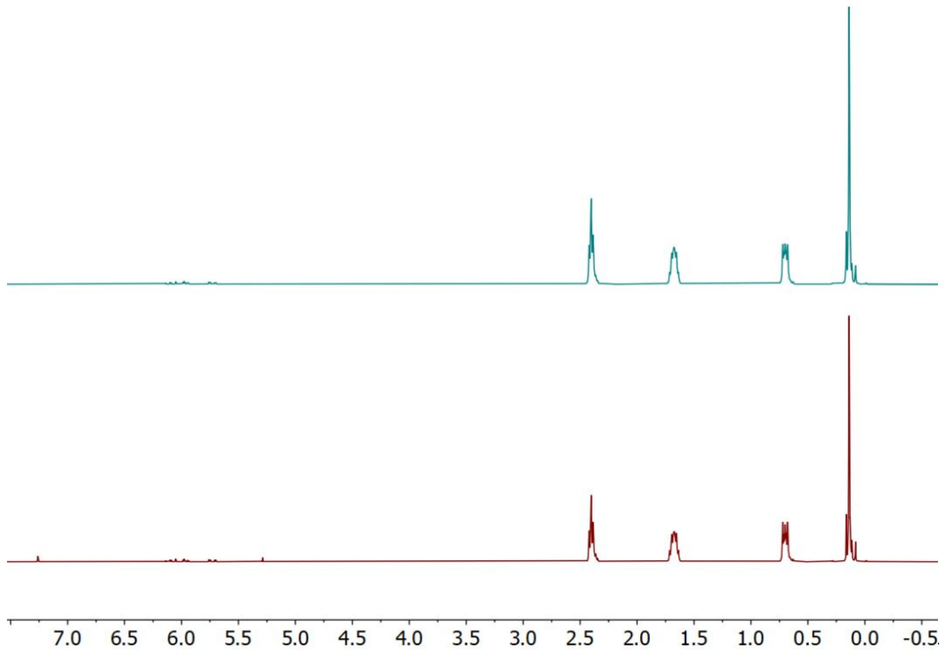

Figure S115. Diffusion-edited NMR of ($\mathbf{P_{CN}}$-**V**) obtained from end-functionalization of long chains (top) and 1H NMR of ($\mathbf{P_{CN}}$-**V**) stacked (bottom).

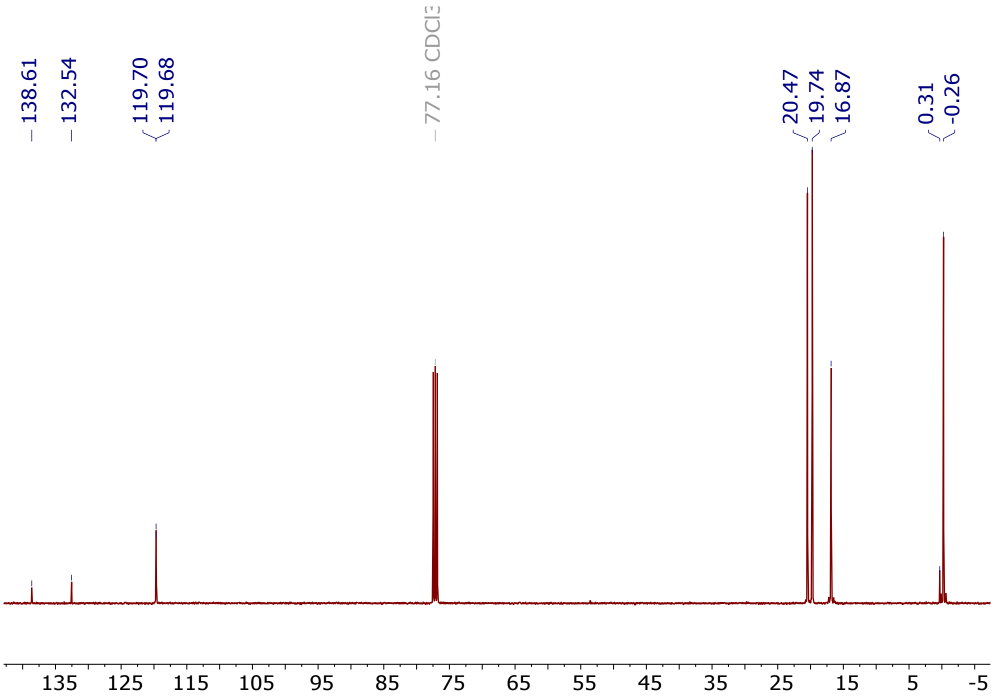

Figure S116. ^{13}C NMR spectrum of polymer ($\mathbf{P_{CN}}$-**V**) obtained from end-functionalization of long chains in $CDCl_3$.

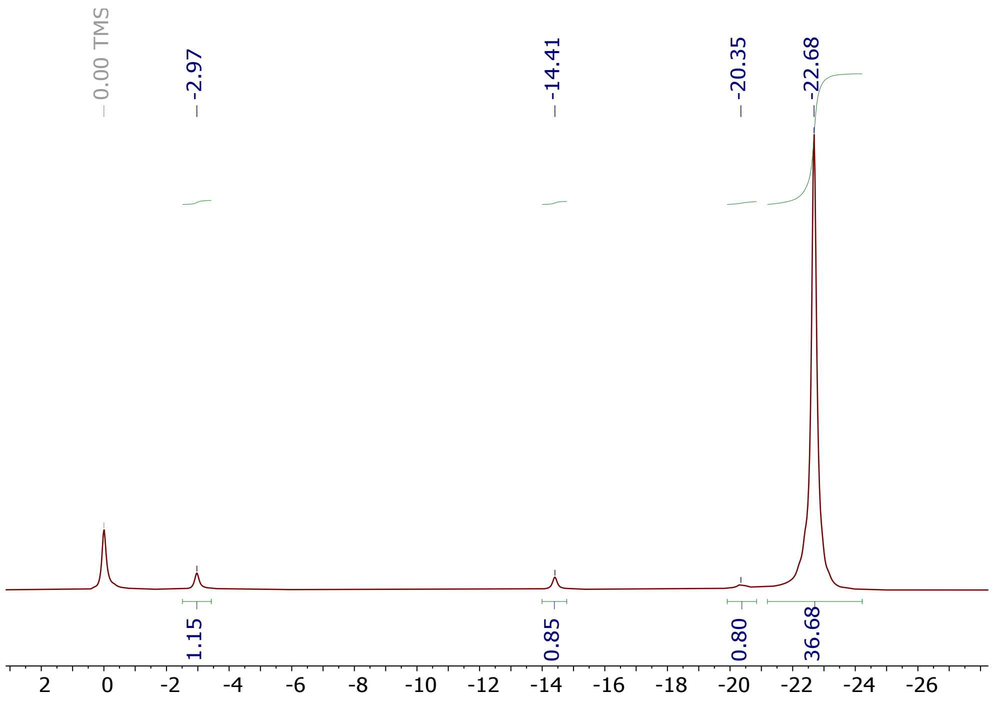

Figure S117. ^{29}Si NMR spectrum of polymer (**P_{CN}-V**) obtained from end-functionalization of long chains. The spectrum was recorded using $Cr(acac)_3$ as a relaxation agent and TMS as a reference.

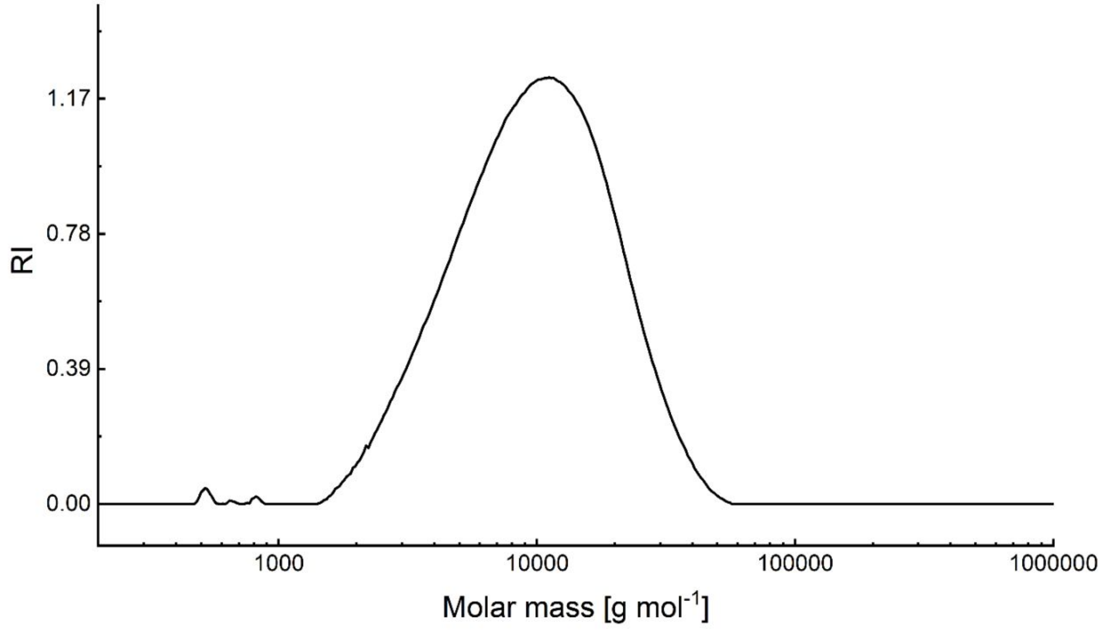

Figure S118. GPC chromatogram of polymer (P_{CN}-V) obtained from end-functionalization of long chains. The GPC was recorded in THF and was calibrated with PS standards.
